# Supplementary material for: Trained immunity of intestinal tuft cells during infancy enhances host defense against enteroviral infections in mice
Source: EMBO Mol Med. 2024 Sep 11;16(10):2516–38. doi: 10.1038/s44321-024-00128-9 (PMC11479266; doi:10.1038/s44321-024-00128-9)
Supplement: Supplementary file 1 — Appendix [file 44321_2024_128_MOESM1_ESM.pdf]

Appendix

Table of Contents

Appendix Table S1...2

Appendix Table S1: The exact p values.

P values were provided as * *P* < 0.05, ** *P* < 0.01, *** *P* < 0.001. *ns*, not significant.

	P value	P value summary
Figure 1B	***	*P<0.001*
	***	*P<0.001*
	***	*P<0.001*
Figure 1C	*	*P=0.0347*
Figure 1E	***	*P<0.001*
	**	*P=0.0023*
	***	*P<0.001*
	ns	*P>0.05*
Figure 1H	**	*P=0.0026*

	P value	*P value*
Figure 2A	**	*P=0.001*
Figure 2B	***	*P=0.005*
Figure 2I	ns	*P>0.05*
	***	*P<0.001*
Figure 2J	ns	*P>0.05*
	*	*P=0.0113*
Figure 2K	ns	*P>0.05*
	***	*p=0.0002*
Figure 2N	*	*P=0.0232*
	ns	*P>0.05*
	*	*P=0.0119*

	P value	*P value*
Figure 3B	**	*P=0.0018*
Figure 3C	*	*P=0.0419*
Figure 3D	ns	*P>0.05*
Figure 3E	ns	*P>0.05*
Figure 3H	***	*P<0.001*
Figure 3I	***	*P=0.0008*
	***	*P<0.001*
	***	*P<0.001*
	**	*P=0.0065*
Figure 3J	**	*P=0.0076*
Figure 3M	***	*P<0.001*
	***	*P<0.001*
	ns	*P>0.05*
Figure 3N	*	*P=0.0107*
	**	*P=0.0017*
	ns	*P>0.05*

Figure 3O	**	*P=0.0052*
	***	*P<0.001*
	ns	*P>0.05*

	P value	*P value*
Figure 4A	*	*P=0.0115*
Figure 4B	***	*P<0.001*
	**	*P=0.0013*
Figure 4D	***	*P<0.001*
Figure 4E	ns	*P>0.05*
	*	*P=0.0377*
Figure 4G	**	*P=0.0065*
Figure 4H	**	*P=0.0015*
	**	*P=0.0093*
	*	*P=0.0138*
Figure 4I	*	*P=0.0102*
	**	*P=0.0053*
	**	*P=0.0015*
Figure 4J	ns	*P>0.05*
	ns	*P>0.05*
	***	*P<0.001*
Figure 4K	**	*P=0.0053*
	***	*P<0.001*
	***	*P<0.001*
Figure 4L	**	*P=0.0011*
	*	*P=0.0158*
	**	*P=0.0031*

	P value	*P value*
Figure 5B	***	*P<0.001*
Figure 5C	***	*P<0.001*
Figure 5D	***	*P<0.001*
Figure 5E	**	*P=0.0091*

	P value	*P value*
Figure 6B	**	*P=0.0013*
Figure 6C	**	*P=0.0023*
Figure 6D	***	*P<0.001*
Figure 6E	**	*P=0.0071*
Figure 6F	***	*P<0.001*
Figure 6G	*	*P=0.0101*
Figure 6H	*	*P=0.0448*
Figure 6I	***	*P<0.001*
Figure 6J	**	*P=0.0035*

Figure 6K	**	*P=0.0019*
Figure 6L	**	*P=0.0092*
Figure 6M	***	*P<0.001*
	ns	*P>0.05*
Figure 6N	**	*P=0.0011*
	ns	*P>0.05*
Figure 6O	**	*P=0.0053*
	ns	*P>0.05*

	P value	*P value*
Figure 7B	ns	*P>0.05*
	ns	*P>0.05*
	*	*P=0.0273*
	*	*P=0.0197*
Figure 7C	**	*P=0.0075*
Figure 7D	*	*P=0.0212*
Figure 7E	*	*P=0.0175*
Figure 7F	*	*P=0.0354*
Figure 7G	*	*P=0.0365*
Figure 7H	***	*P=0.0007*

	P value	*P value*
Figure 8B	**	*P=0.004*
	**	*P=0.0073*
	***	*P=0.0008*
	ns	*P>0.05*
Figrue 8C	***	*P<0.001*
	**	*P=0.0097*
	***	*P<0.001*
	ns	*P>0.05*

	P value	*P value*
Figure EV2C	***	*P<0.001*
	***	*P<0.001*
	**	*P=0.0033*
