# Supplementary figures and images for: Trained immunity of intestinal tuft cells during infancy enhances host defense against enteroviral infections in mice
Source: EMBO Mol Med. 2024 Sep 11;16(10):2516–38. doi: 10.1038/s44321-024-00128-9 (PMC11479266; doi:10.1038/s44321-024-00128-9)

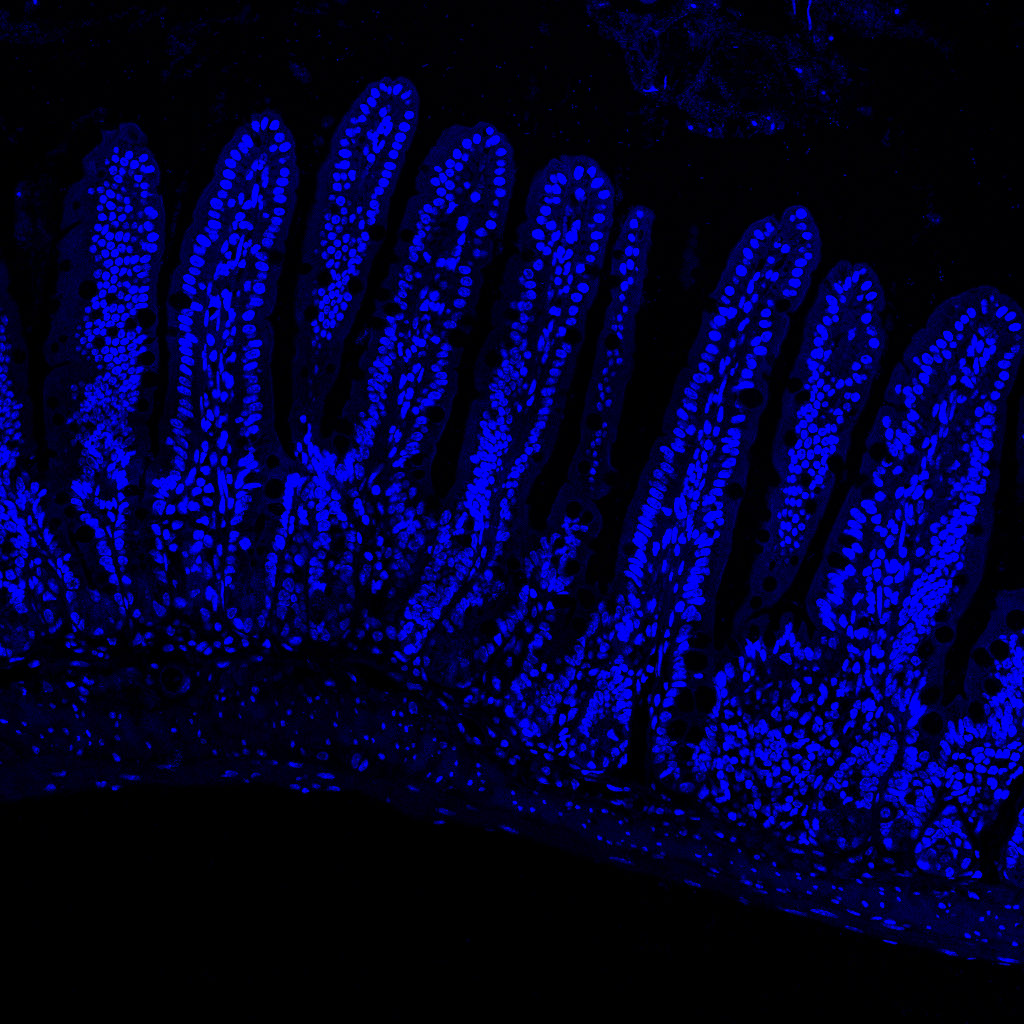

Supplement: Supplementary file 3 — Source data Fig. 1 [file 44321_2024_128_MOESM3_ESM.zip › EMM-2023-19008-V2-figure 1/EMM-2023-19008-V2-figure 1/figure 1D IF/EV71-Infected/14dpi/EV71 14dpi DCALK1 594-20x1-3_0001.tif.╥╤╙├frames/14dpi DCALK1 594-20x1-3_0001_C001T001.tif]

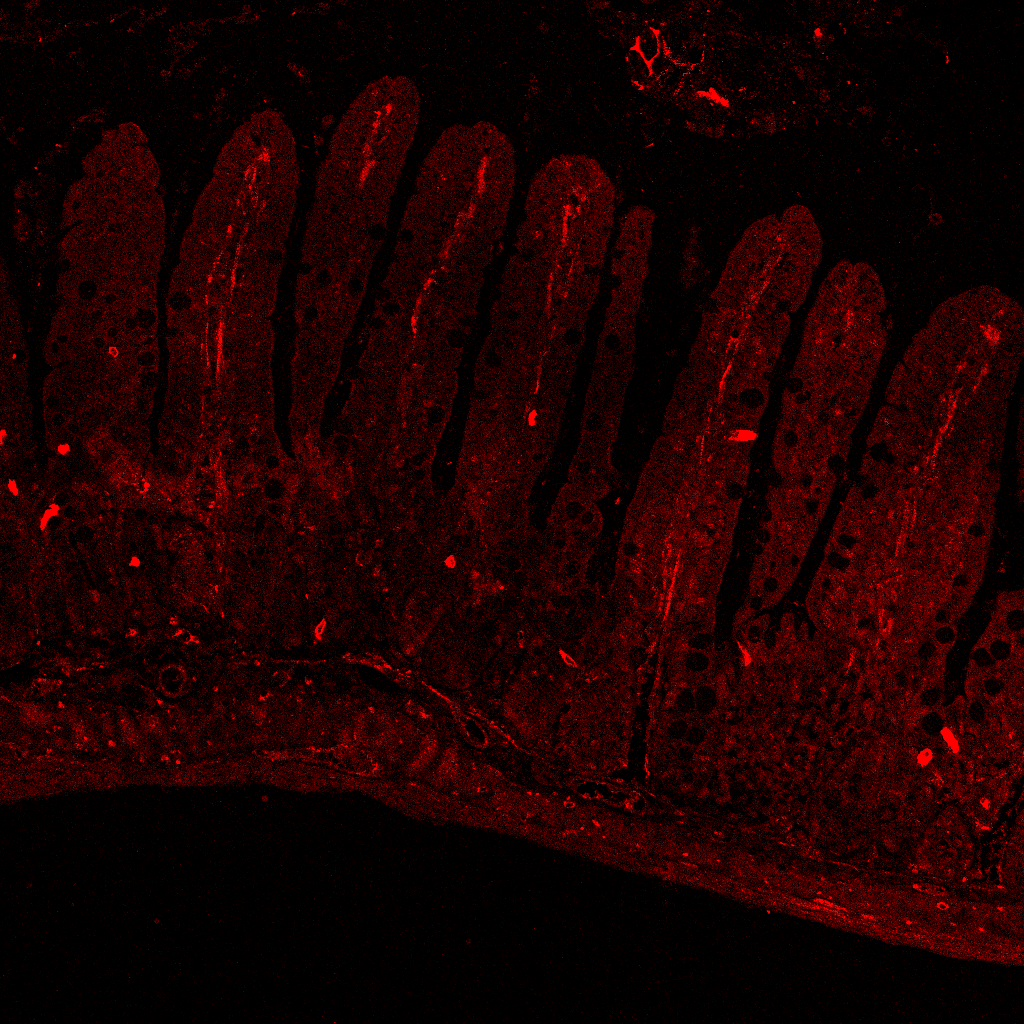

Supplement: Supplementary file 3 — Source data Fig. 1 [file 44321_2024_128_MOESM3_ESM.zip › EMM-2023-19008-V2-figure 1/EMM-2023-19008-V2-figure 1/figure 1D IF/EV71-Infected/14dpi/EV71 14dpi DCALK1 594-20x1-3_0001.tif.╥╤╙├frames/14dpi DCALK1 594-20x1-3_0001_C002T001.tif]

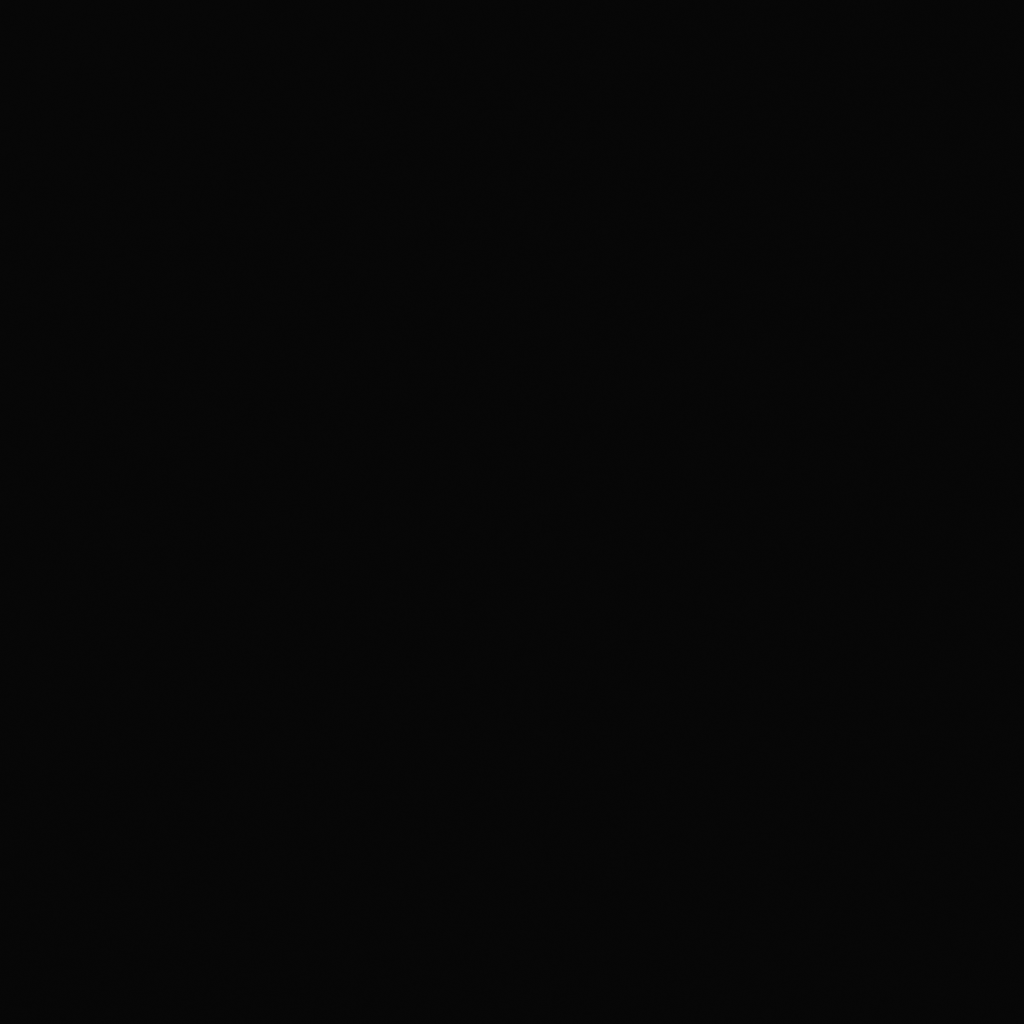

Supplement: Supplementary file 3 — Source data Fig. 1 [file 44321_2024_128_MOESM3_ESM.zip › EMM-2023-19008-V2-figure 1/EMM-2023-19008-V2-figure 1/figure 1D IF/EV71-Infected/14dpi/EV71 14dpi DCALK1 594-20x1-3_0001.tif.╥╤╙├frames/14dpi DCALK1 594-20x1-3_0001_C003T001.tif]

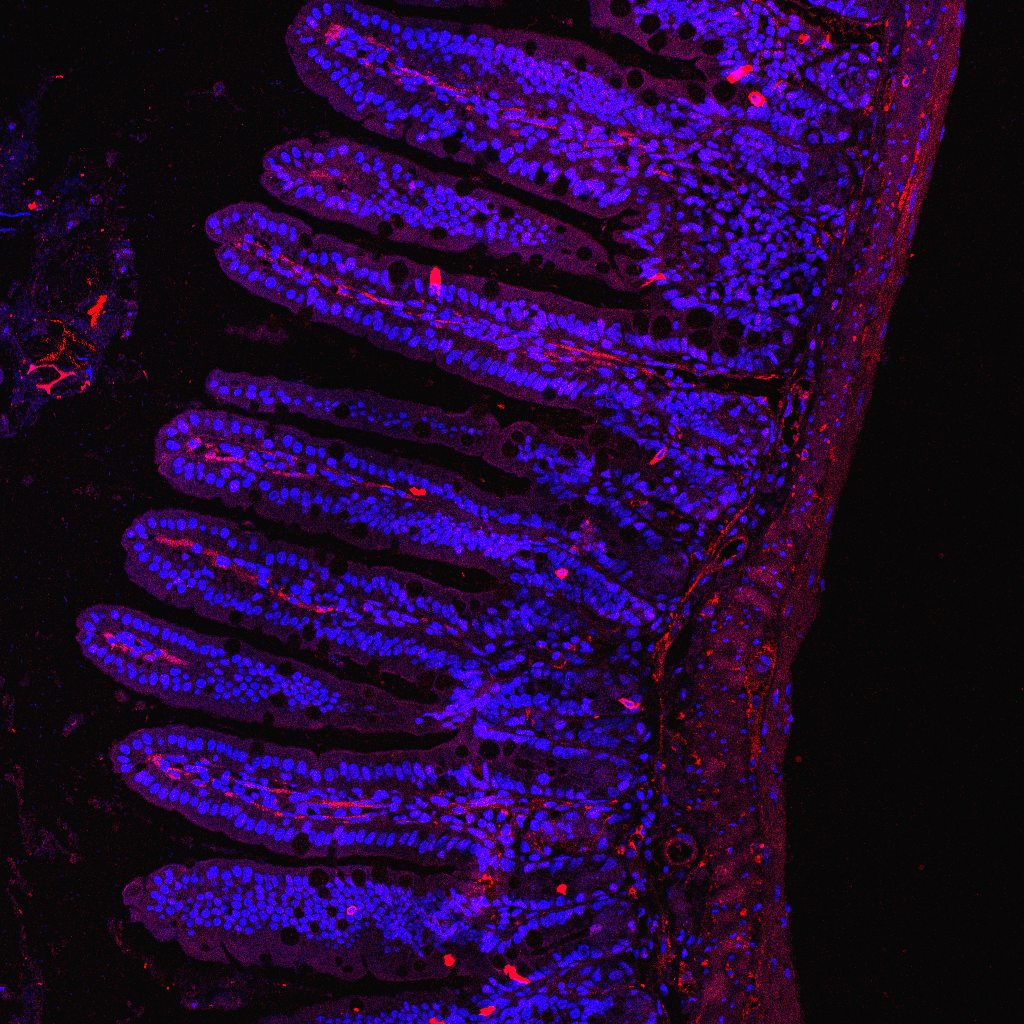

Supplement: Supplementary file 3 — Source data Fig. 1 [file 44321_2024_128_MOESM3_ESM.zip › EMM-2023-19008-V2-figure 1/EMM-2023-19008-V2-figure 1/figure 1D IF/EV71-Infected/14dpi/EV71 14dpi DCALK1 594-20x1-3_0001.tif.╥╤╙├frames/14dpi DCALK1 594-20x1-3_0001_T001.tif]

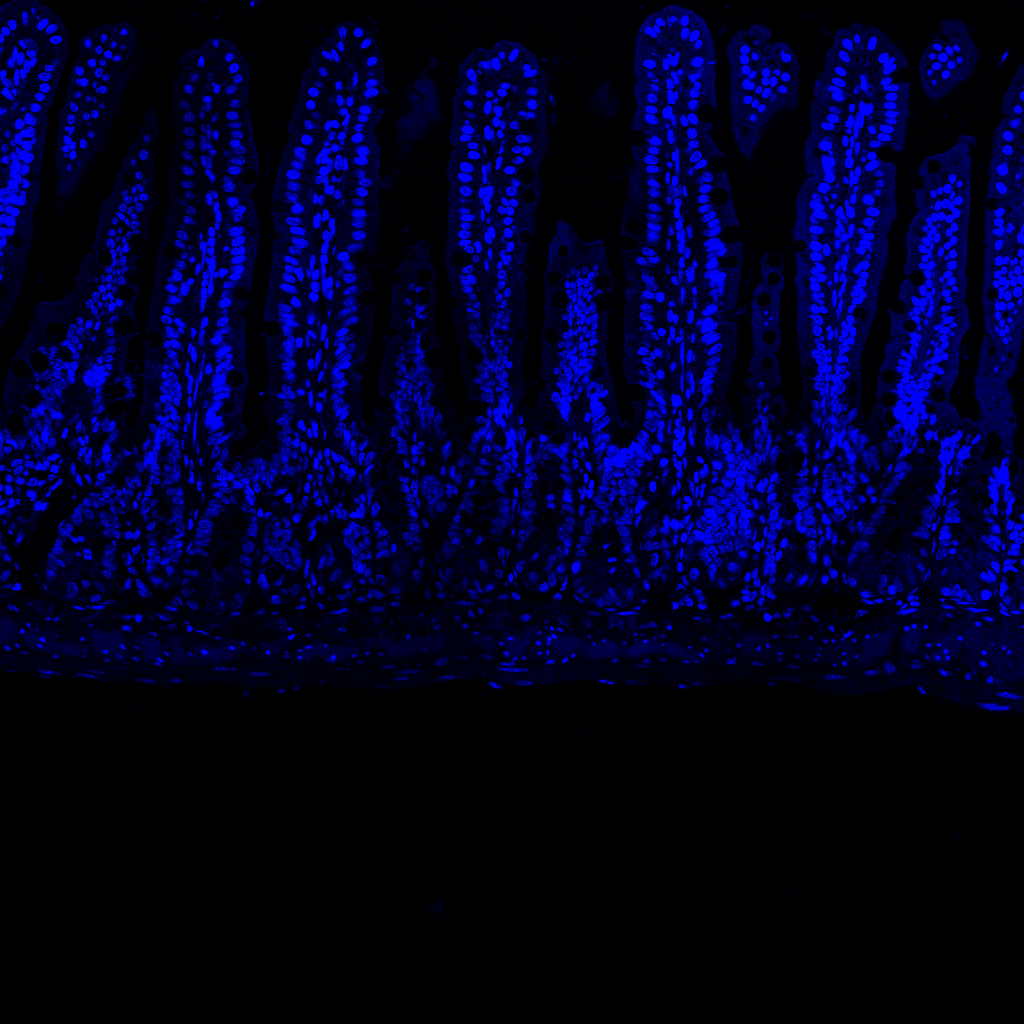

Supplement: Supplementary file 3 — Source data Fig. 1 [file 44321_2024_128_MOESM3_ESM.zip › EMM-2023-19008-V2-figure 1/EMM-2023-19008-V2-figure 1/figure 1D IF/EV71-Infected/3dpi/3dpi DCALK1 594-20x1-1_0001_C001T001.tif]

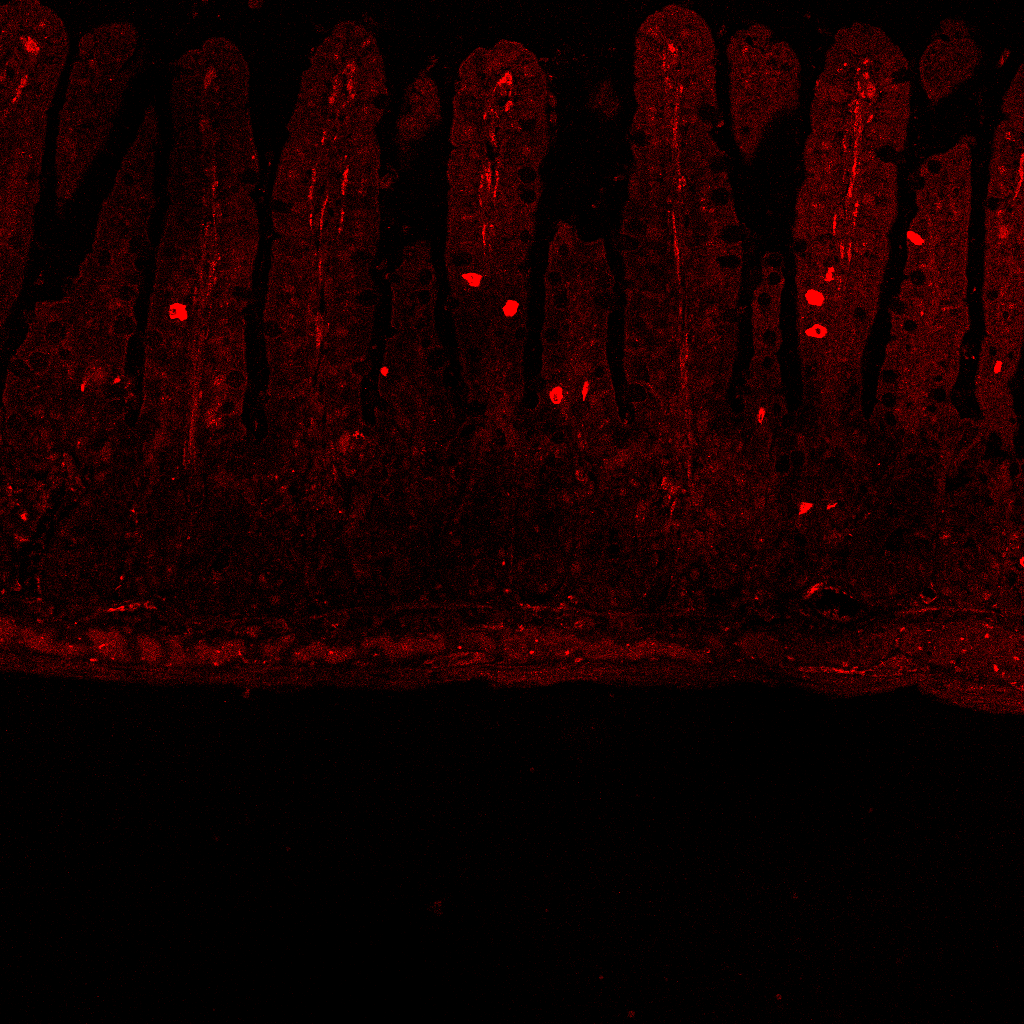

Supplement: Supplementary file 3 — Source data Fig. 1 [file 44321_2024_128_MOESM3_ESM.zip › EMM-2023-19008-V2-figure 1/EMM-2023-19008-V2-figure 1/figure 1D IF/EV71-Infected/3dpi/3dpi DCALK1 594-20x1-1_0001_C002T001.tif]

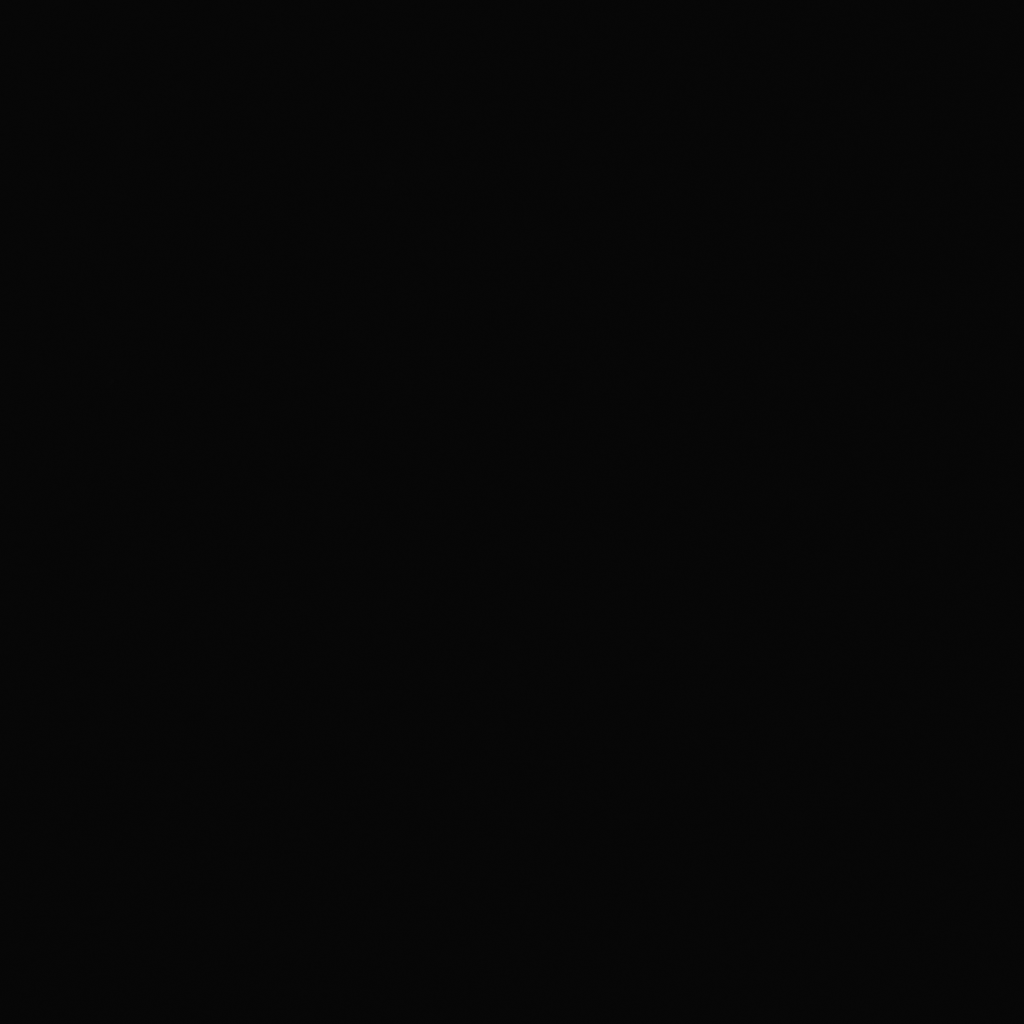

Supplement: Supplementary file 3 — Source data Fig. 1 [file 44321_2024_128_MOESM3_ESM.zip › EMM-2023-19008-V2-figure 1/EMM-2023-19008-V2-figure 1/figure 1D IF/EV71-Infected/3dpi/3dpi DCALK1 594-20x1-1_0001_C003T001.tif]

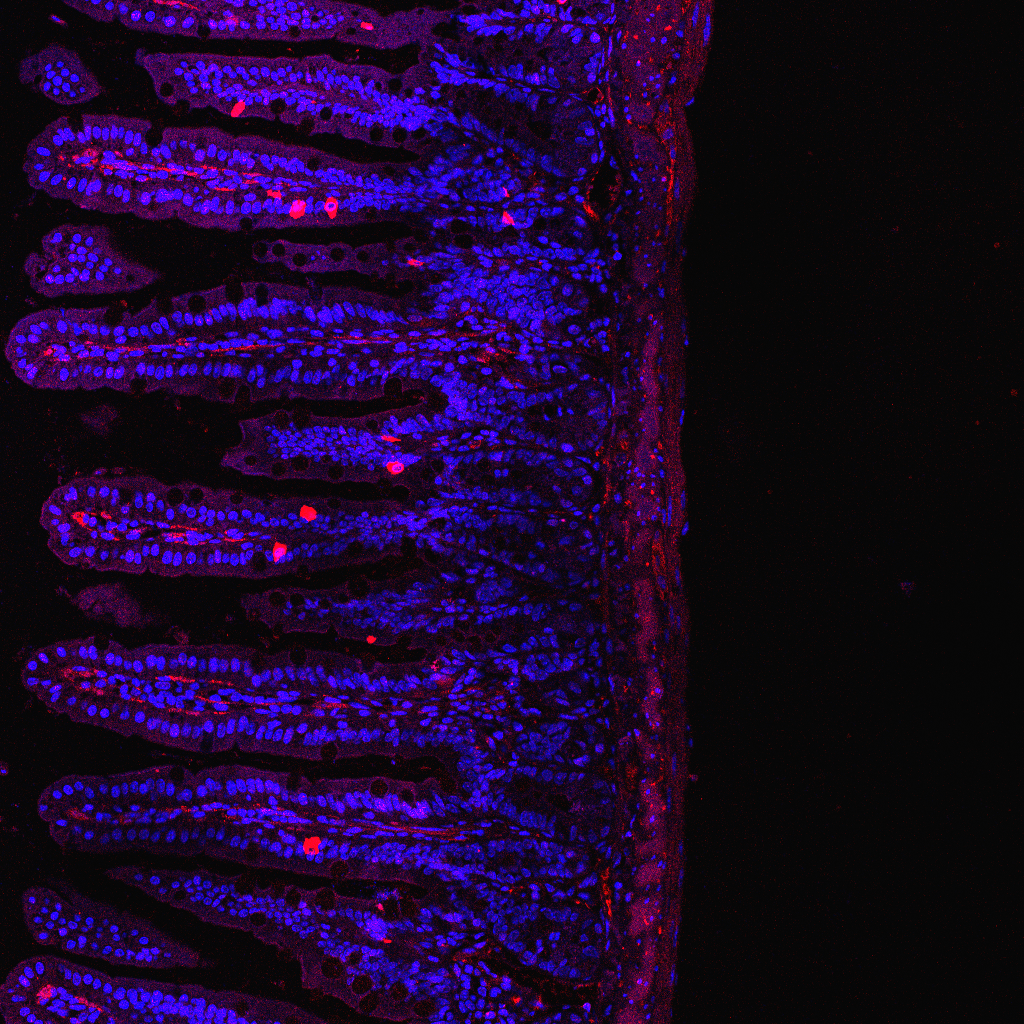

Supplement: Supplementary file 3 — Source data Fig. 1 [file 44321_2024_128_MOESM3_ESM.zip › EMM-2023-19008-V2-figure 1/EMM-2023-19008-V2-figure 1/figure 1D IF/EV71-Infected/3dpi/3dpi DCALK1 594-20x1-1_0001_T001.tif]

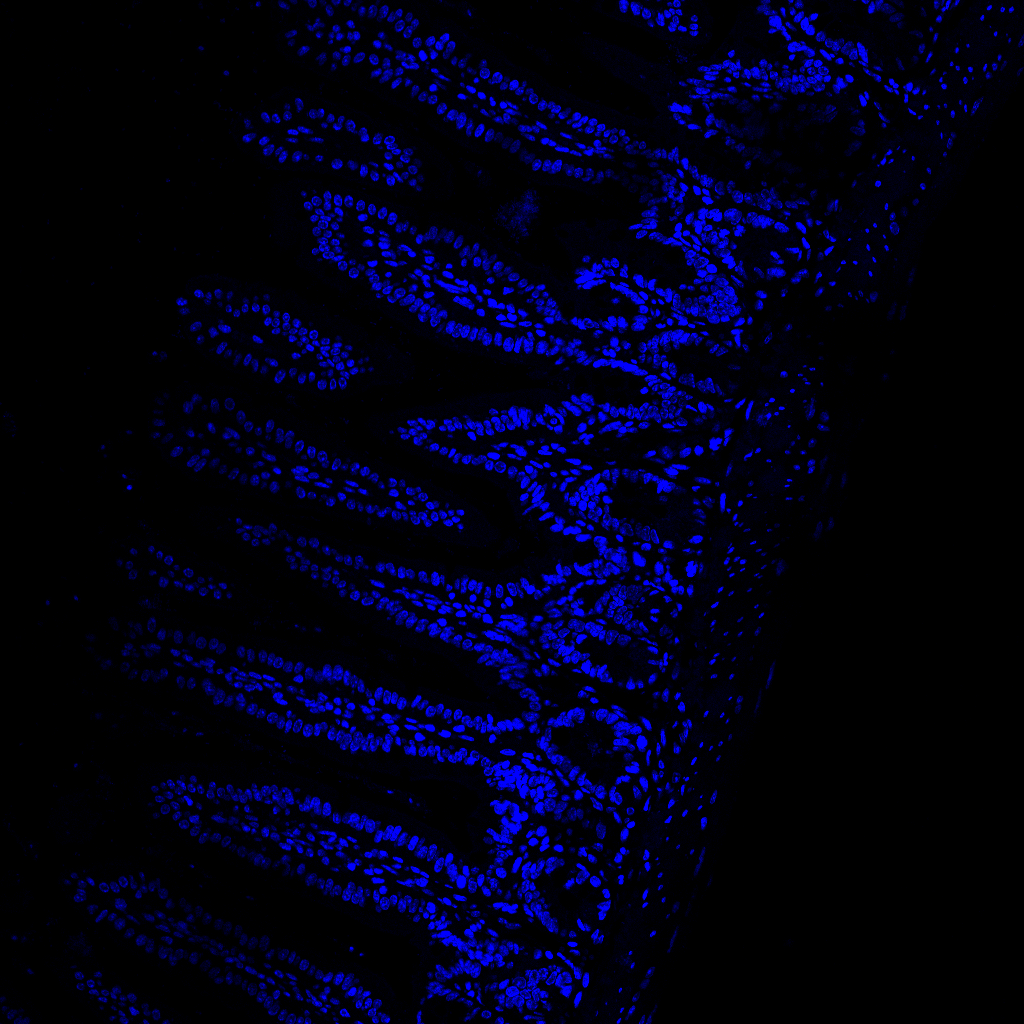

Supplement: Supplementary file 3 — Source data Fig. 1 [file 44321_2024_128_MOESM3_ESM.zip › EMM-2023-19008-V2-figure 1/EMM-2023-19008-V2-figure 1/figure 1D IF/EV71-Infected/5dpi/EV71 5dpi DCALK1 594-20x1-1.tif.╥╤╙├frames/5dpi DCALK1 594-20x1-1_C001T001.tif]

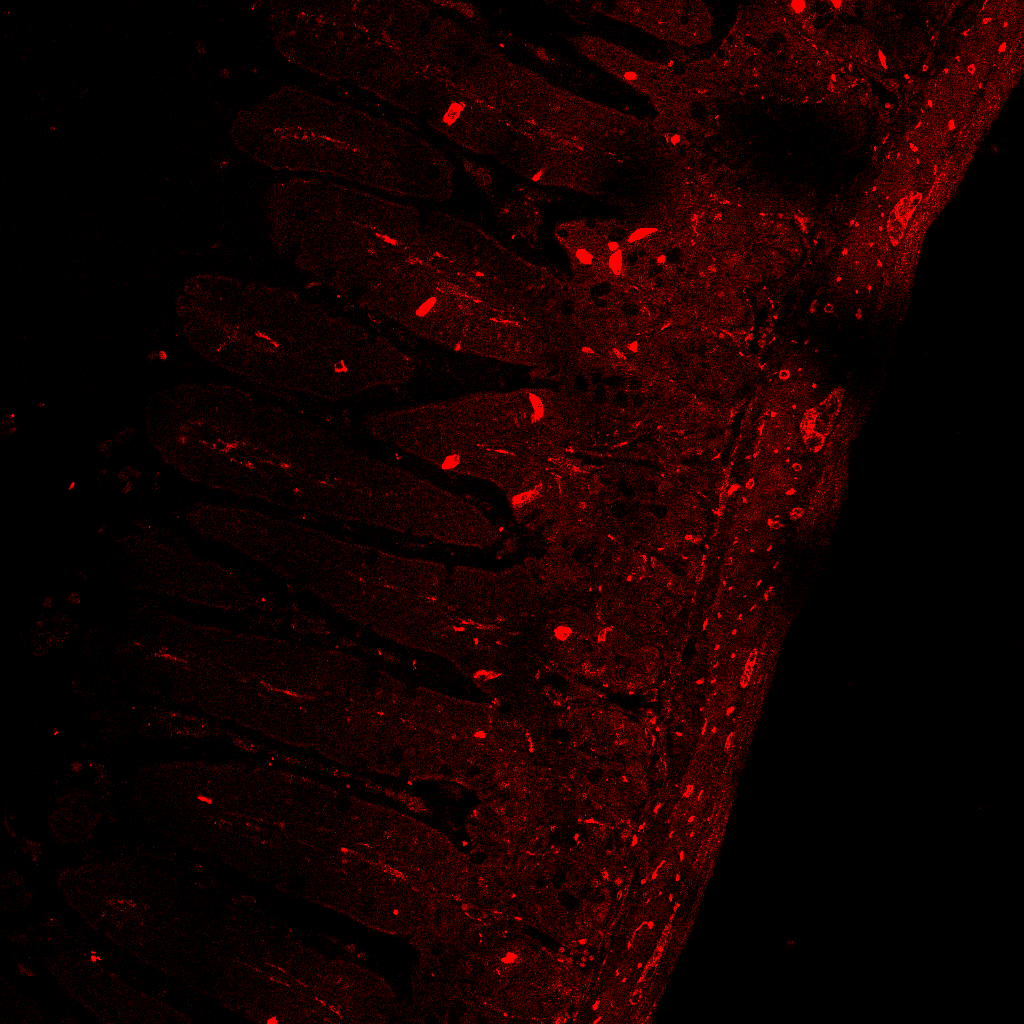

Supplement: Supplementary file 3 — Source data Fig. 1 [file 44321_2024_128_MOESM3_ESM.zip › EMM-2023-19008-V2-figure 1/EMM-2023-19008-V2-figure 1/figure 1D IF/EV71-Infected/5dpi/EV71 5dpi DCALK1 594-20x1-1.tif.╥╤╙├frames/5dpi DCALK1 594-20x1-1_C002T001.tif]

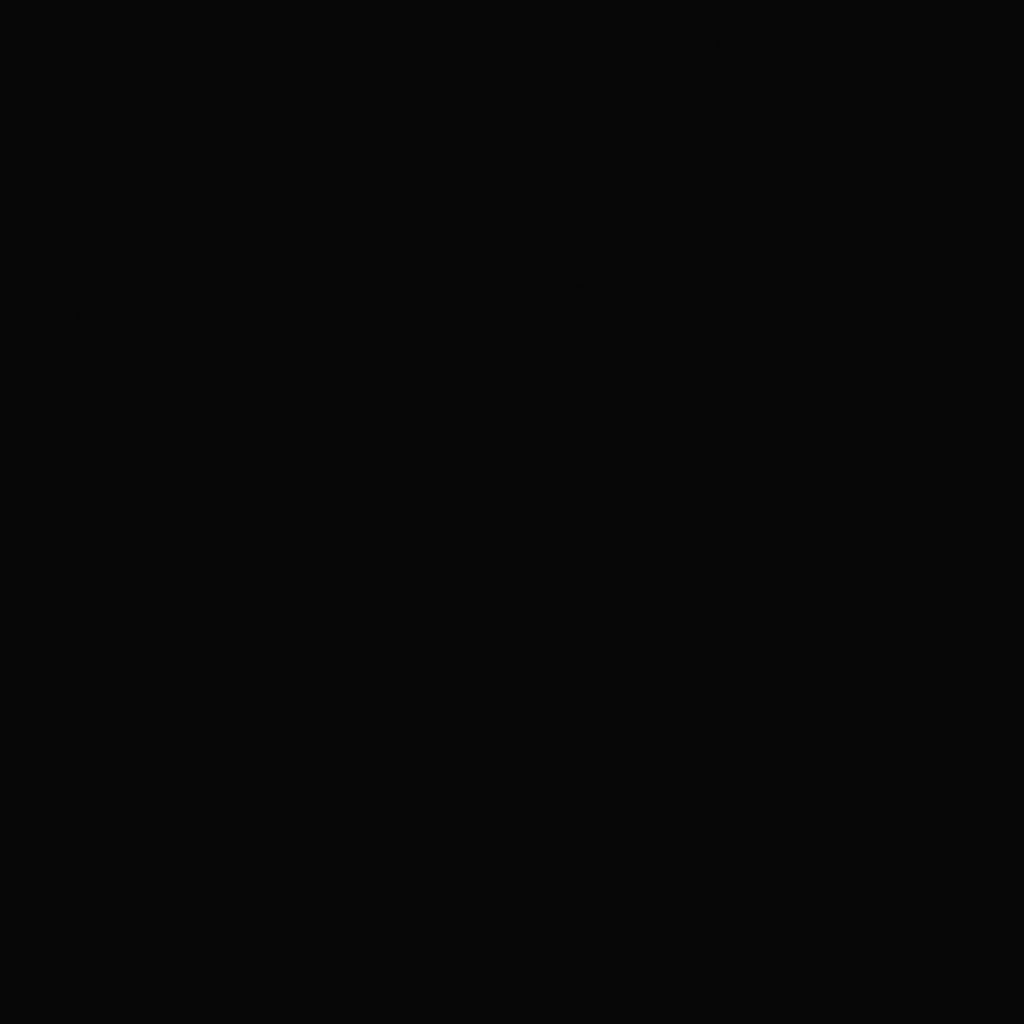

Supplement: Supplementary file 3 — Source data Fig. 1 [file 44321_2024_128_MOESM3_ESM.zip › EMM-2023-19008-V2-figure 1/EMM-2023-19008-V2-figure 1/figure 1D IF/EV71-Infected/5dpi/EV71 5dpi DCALK1 594-20x1-1.tif.╥╤╙├frames/5dpi DCALK1 594-20x1-1_C003T001.tif]

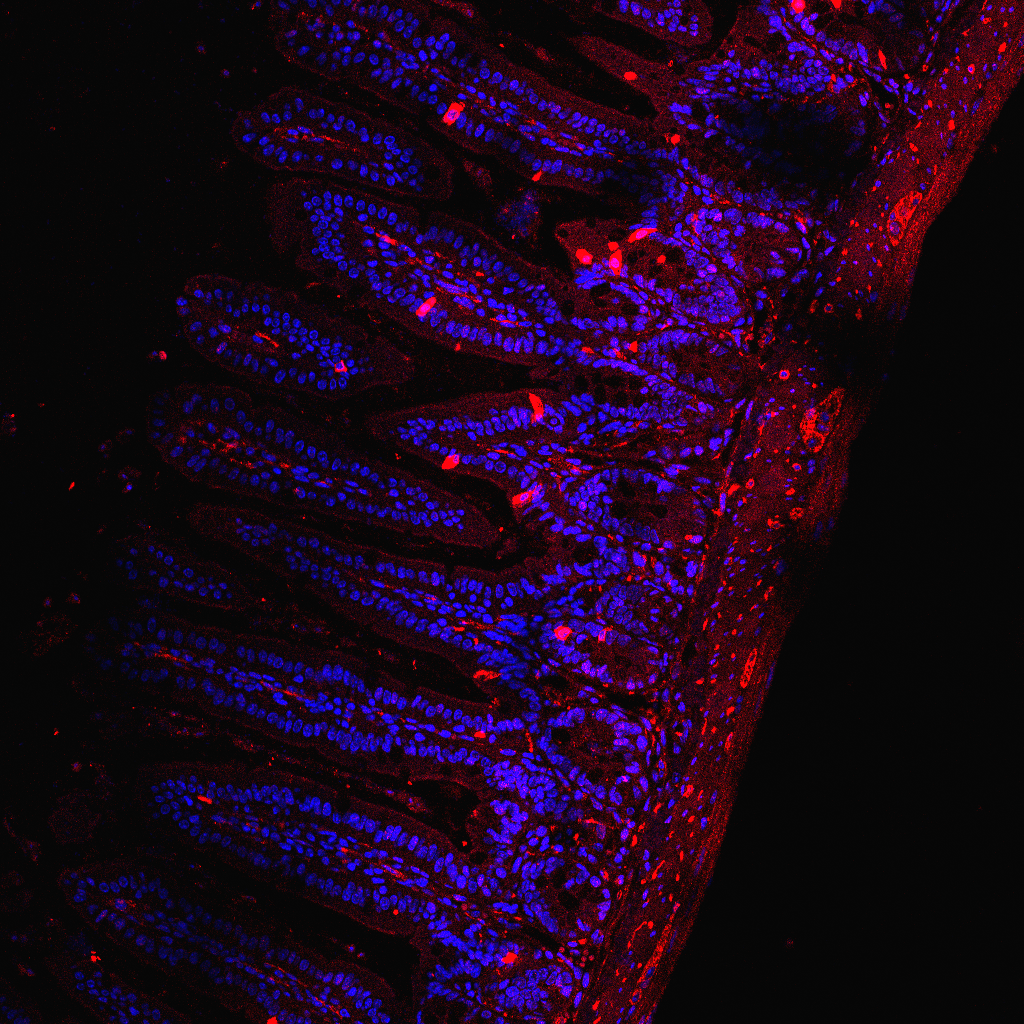

Supplement: Supplementary file 3 — Source data Fig. 1 [file 44321_2024_128_MOESM3_ESM.zip › EMM-2023-19008-V2-figure 1/EMM-2023-19008-V2-figure 1/figure 1D IF/EV71-Infected/5dpi/EV71 5dpi DCALK1 594-20x1-1.tif.╥╤╙├frames/5dpi DCALK1 594-20x1-1_T001.tif]

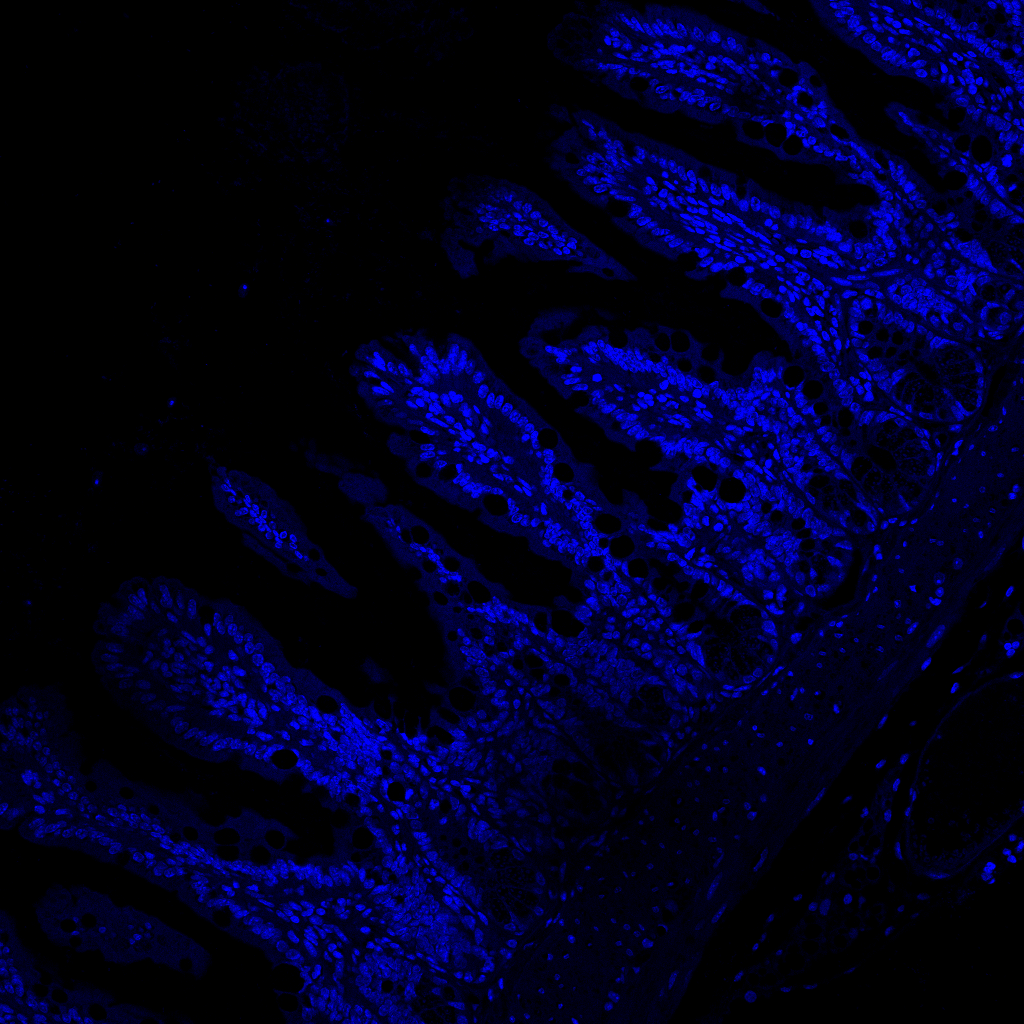

Supplement: Supplementary file 3 — Source data Fig. 1 [file 44321_2024_128_MOESM3_ESM.zip › EMM-2023-19008-V2-figure 1/EMM-2023-19008-V2-figure 1/figure 1D IF/EV71-Infected/7dpi/7dpi DCALK1 594-20x1-2.tif. ╥╤╙├frames/7dpi DCALK1 594-20x1-2_C001T001.tif]

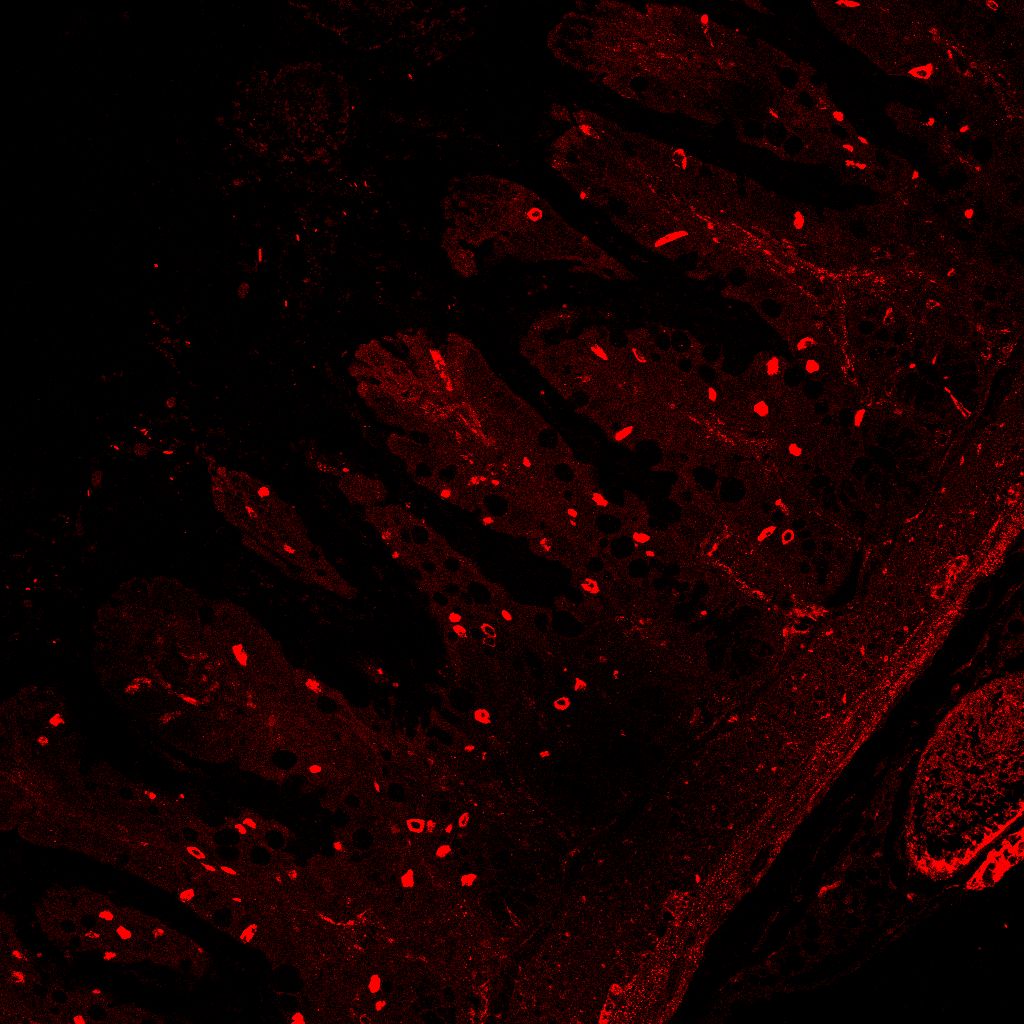

Supplement: Supplementary file 3 — Source data Fig. 1 [file 44321_2024_128_MOESM3_ESM.zip › EMM-2023-19008-V2-figure 1/EMM-2023-19008-V2-figure 1/figure 1D IF/EV71-Infected/7dpi/7dpi DCALK1 594-20x1-2.tif. ╥╤╙├frames/7dpi DCALK1 594-20x1-2_C002T001.tif]

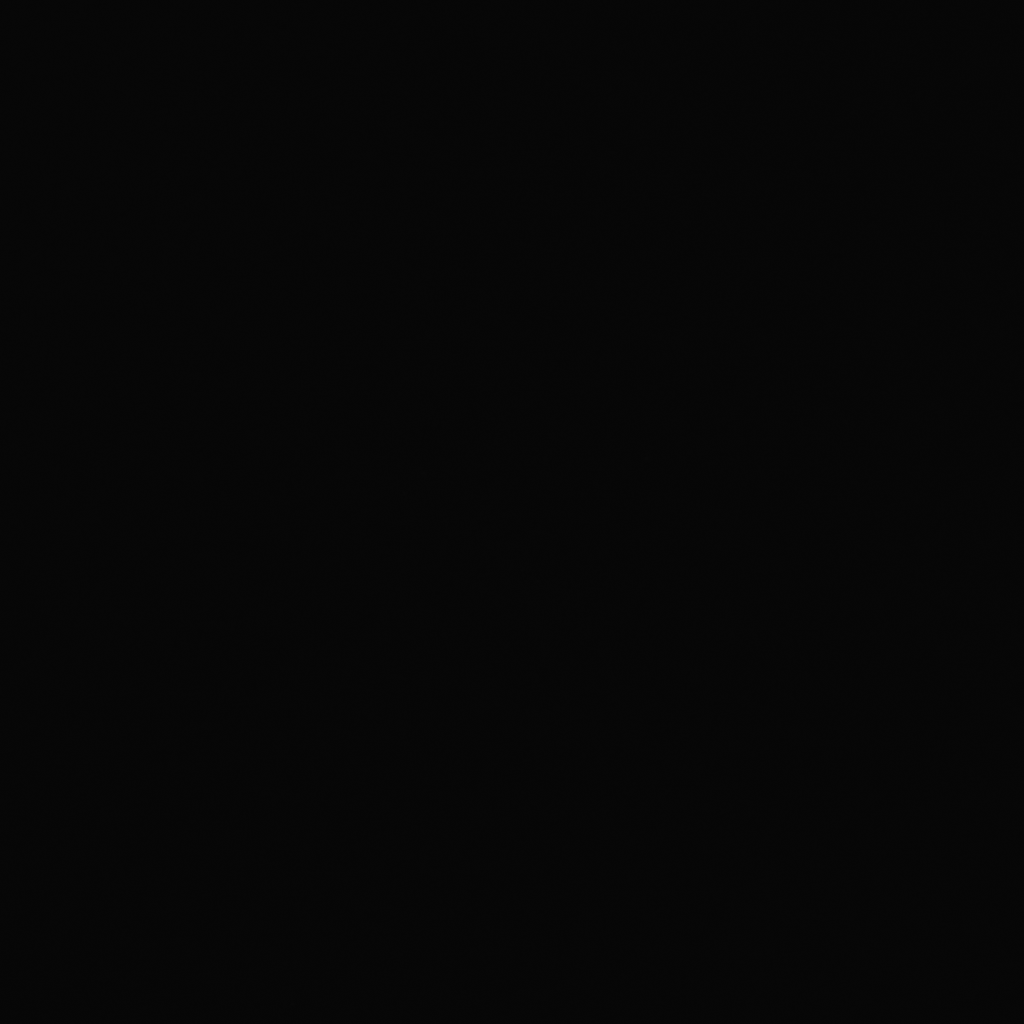

Supplement: Supplementary file 3 — Source data Fig. 1 [file 44321_2024_128_MOESM3_ESM.zip › EMM-2023-19008-V2-figure 1/EMM-2023-19008-V2-figure 1/figure 1D IF/EV71-Infected/7dpi/7dpi DCALK1 594-20x1-2.tif. ╥╤╙├frames/7dpi DCALK1 594-20x1-2_C003T001.tif]

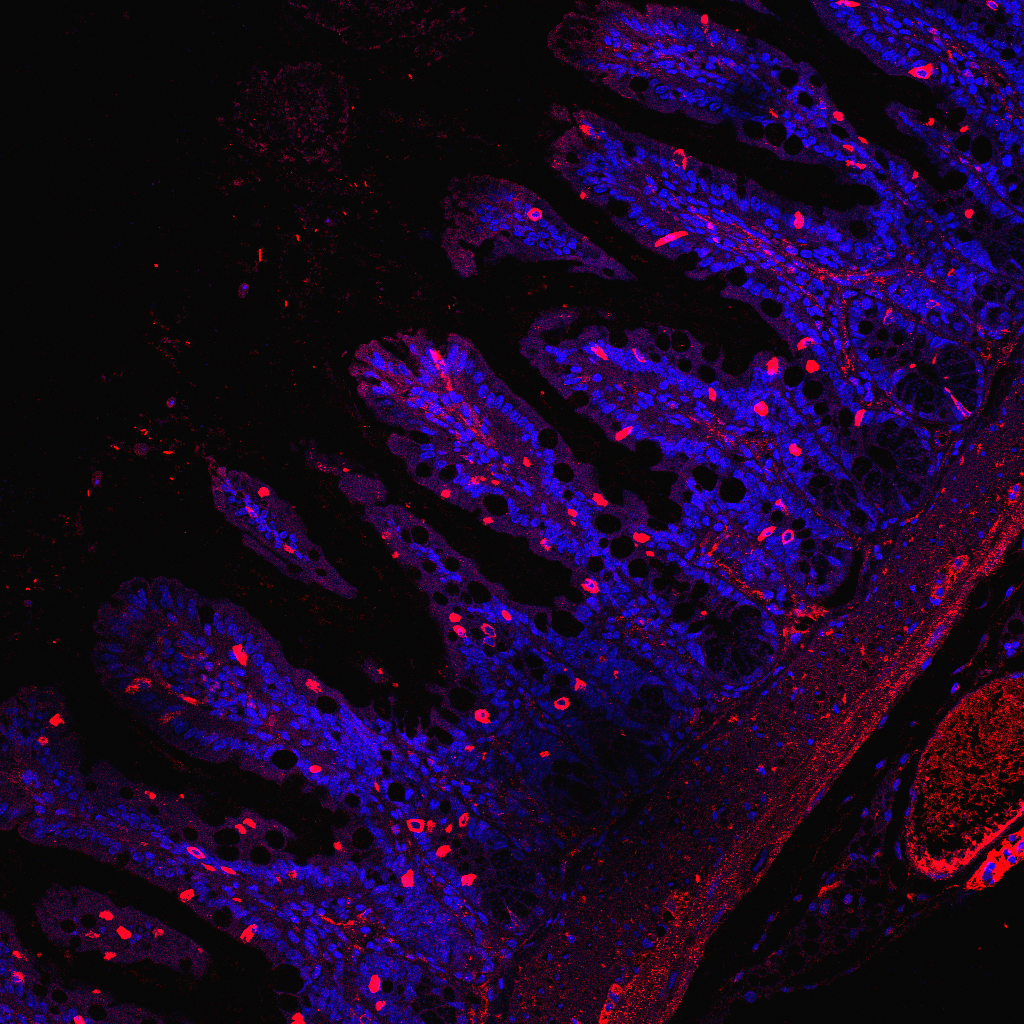

Supplement: Supplementary file 3 — Source data Fig. 1 [file 44321_2024_128_MOESM3_ESM.zip › EMM-2023-19008-V2-figure 1/EMM-2023-19008-V2-figure 1/figure 1D IF/EV71-Infected/7dpi/7dpi DCALK1 594-20x1-2.tif. ╥╤╙├frames/7dpi DCALK1 594-20x1-2_T001.tif]

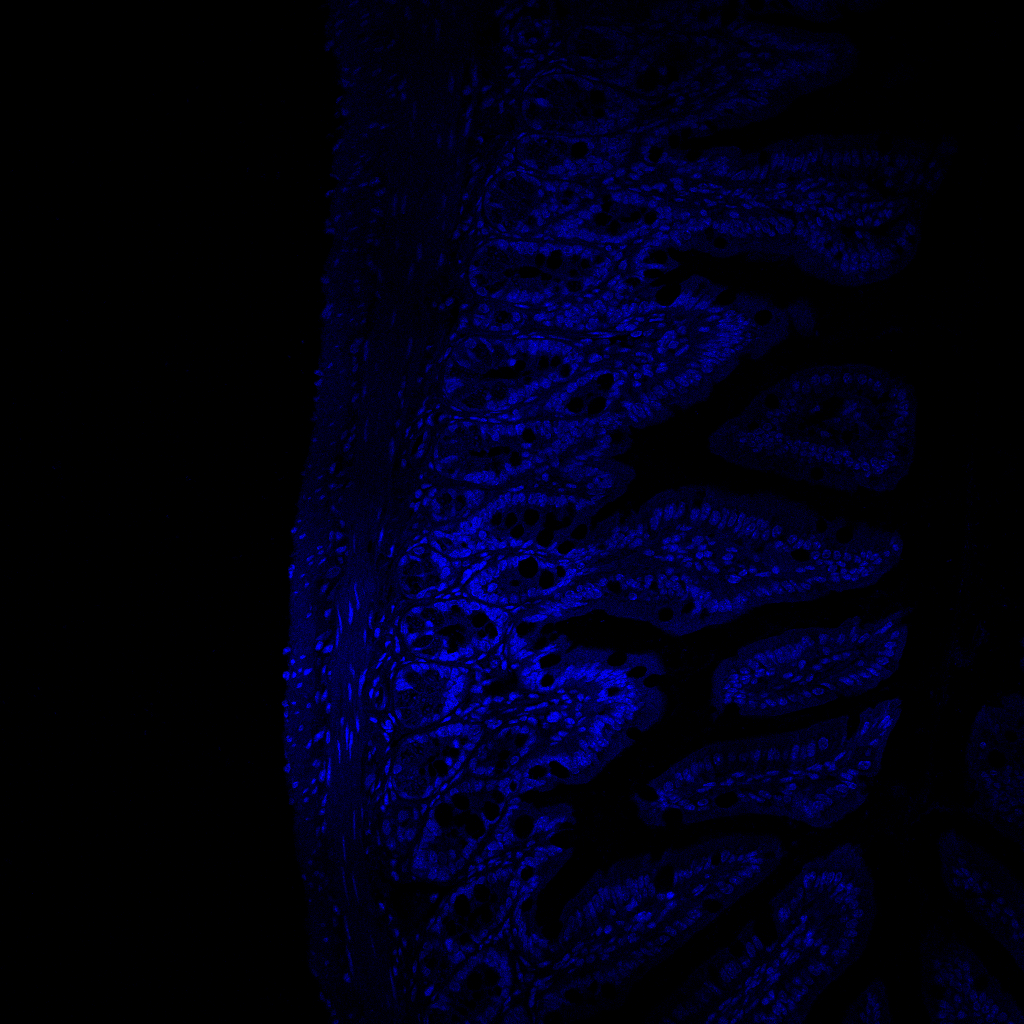

Supplement: Supplementary file 3 — Source data Fig. 1 [file 44321_2024_128_MOESM3_ESM.zip › EMM-2023-19008-V2-figure 1/EMM-2023-19008-V2-figure 1/figure 1D IF/PBS/14dpi/PBS 14dpi DCALK1 594-20x1-1.tif.╥╤╙├frames/PBS 14dpi DCALK1 594-20x1-1_C001T001.tif]

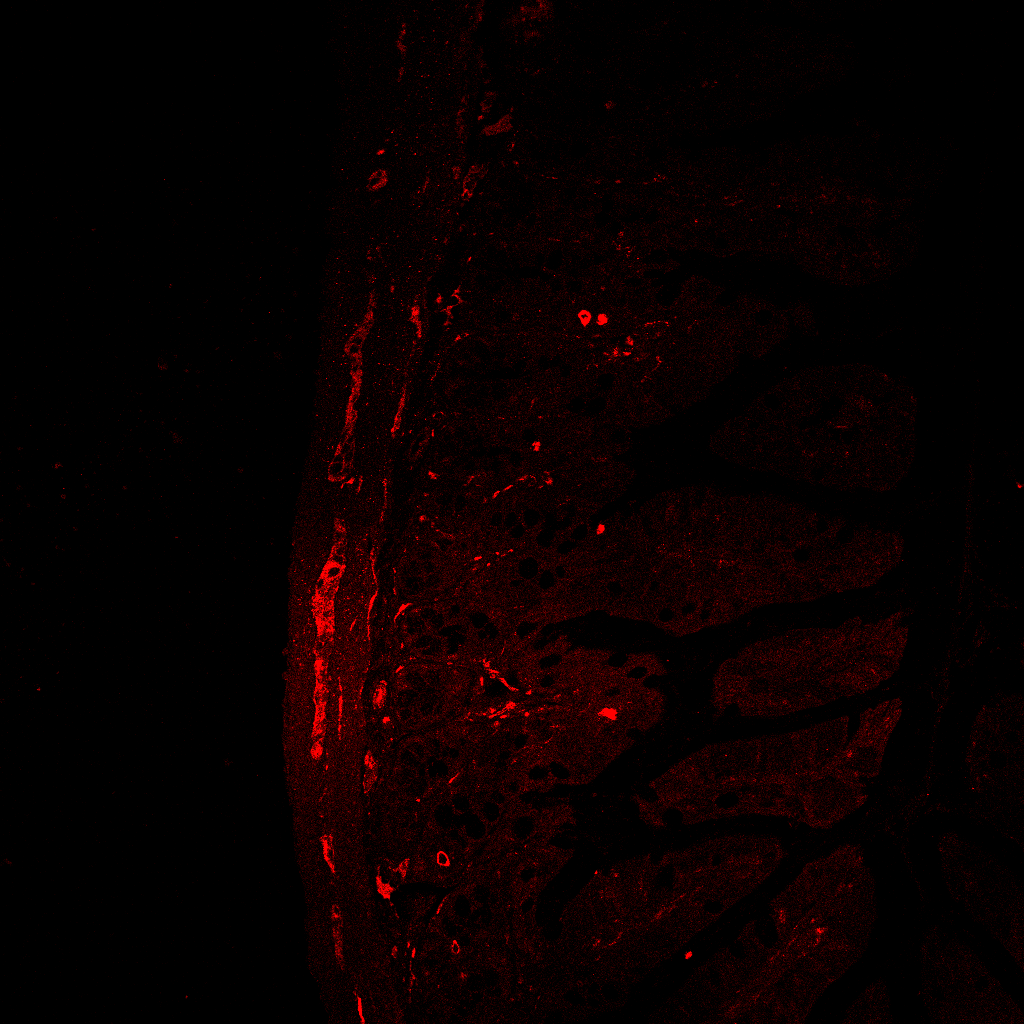

Supplement: Supplementary file 3 — Source data Fig. 1 [file 44321_2024_128_MOESM3_ESM.zip › EMM-2023-19008-V2-figure 1/EMM-2023-19008-V2-figure 1/figure 1D IF/PBS/14dpi/PBS 14dpi DCALK1 594-20x1-1.tif.╥╤╙├frames/PBS 14dpi DCALK1 594-20x1-1_C002T001.tif]

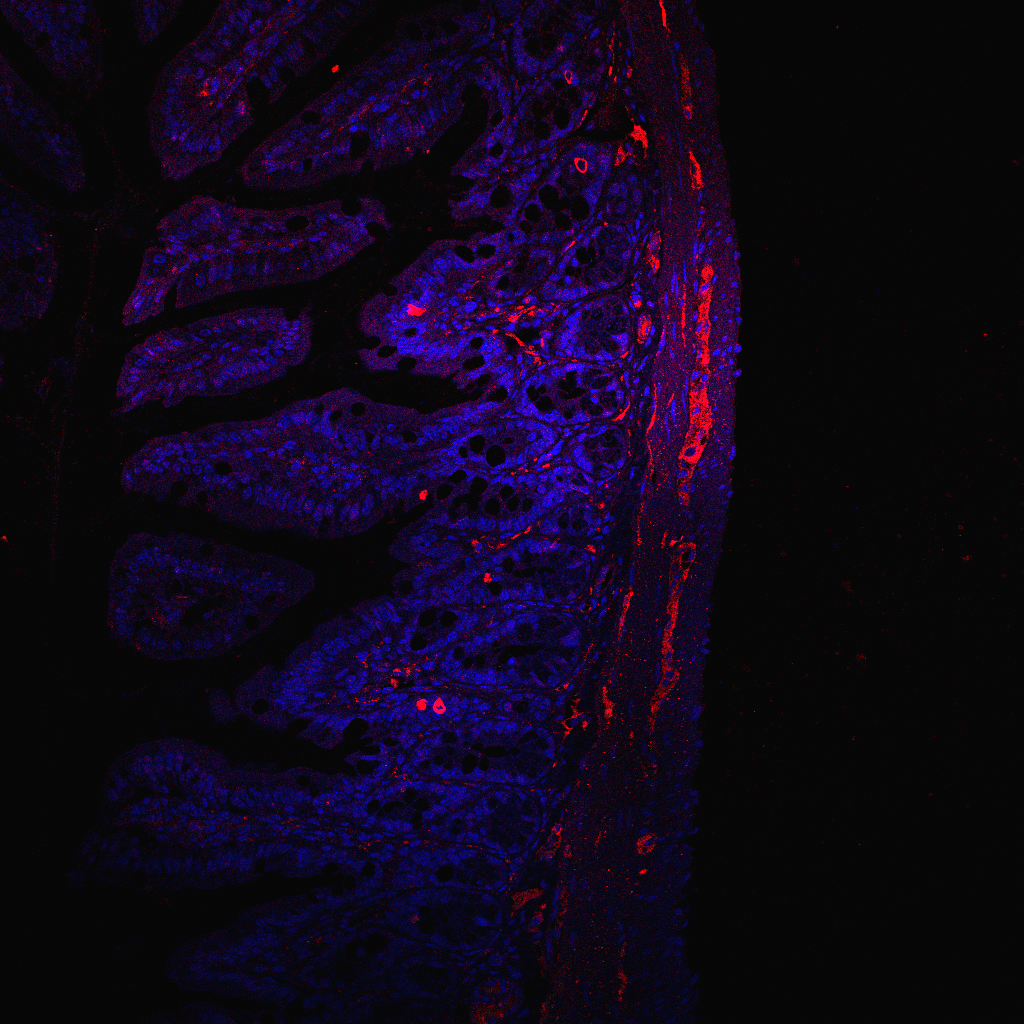

Supplement: Supplementary file 3 — Source data Fig. 1 [file 44321_2024_128_MOESM3_ESM.zip › EMM-2023-19008-V2-figure 1/EMM-2023-19008-V2-figure 1/figure 1D IF/PBS/14dpi/PBS 14dpi DCALK1 594-20x1-1.tif.╥╤╙├frames/PBS 14dpi DCALK1 594-20x1-1_T001.tif]

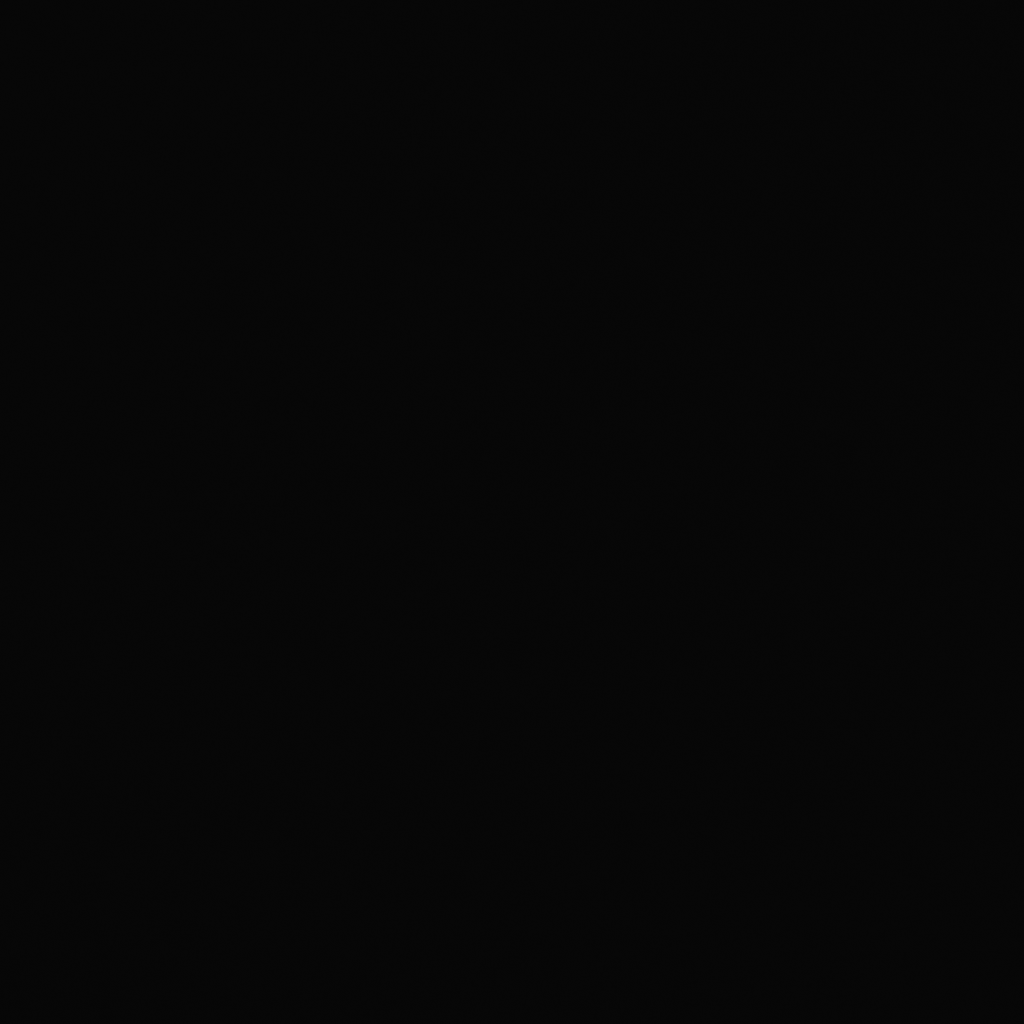

Supplement: Supplementary file 3 — Source data Fig. 1 [file 44321_2024_128_MOESM3_ESM.zip › EMM-2023-19008-V2-figure 1/EMM-2023-19008-V2-figure 1/figure 1D IF/PBS/14dpi/PBS 14dpi DCALK1 594-20x1-1.tif.╥╤╙├frames/PBS14dpi DCALK1 594-20x1-1_C003T001.tif]

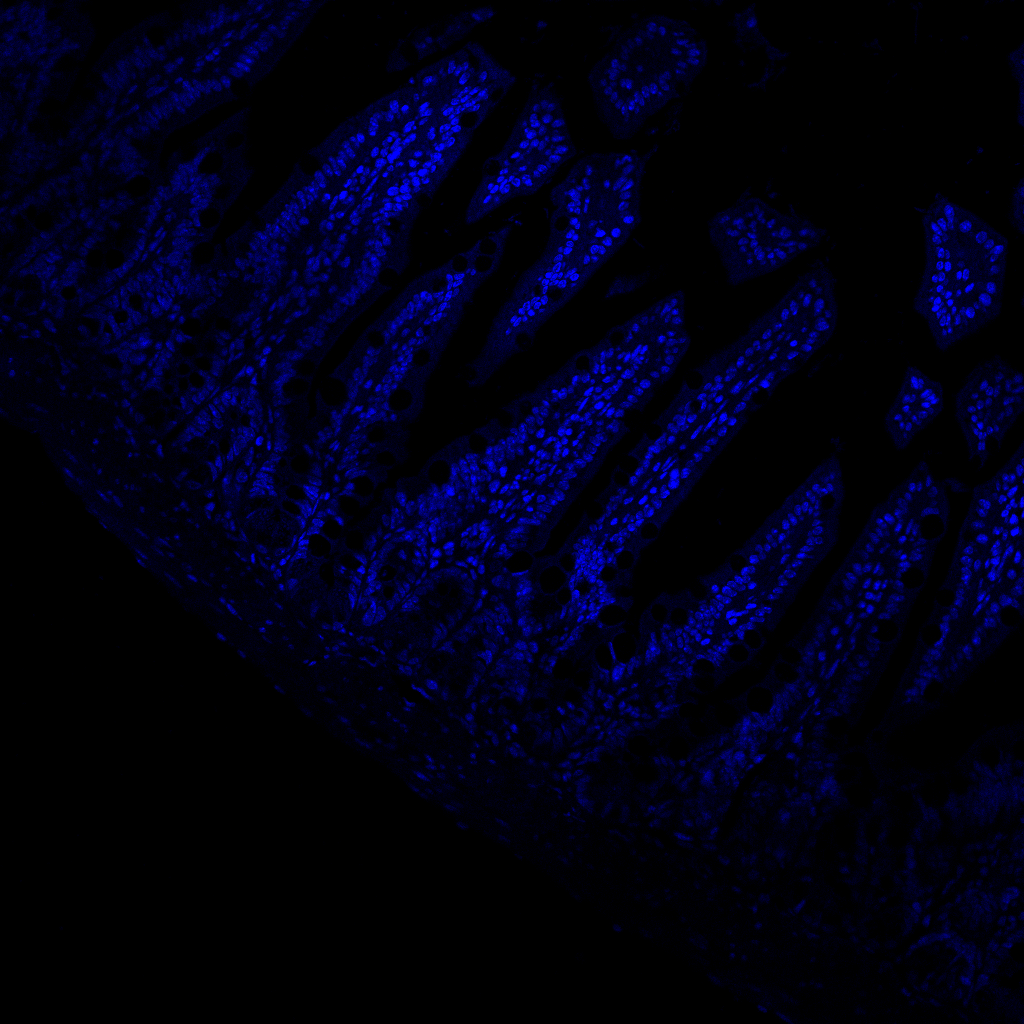

Supplement: Supplementary file 3 — Source data Fig. 1 [file 44321_2024_128_MOESM3_ESM.zip › EMM-2023-19008-V2-figure 1/EMM-2023-19008-V2-figure 1/figure 1D IF/PBS/3dpi/PBS 3dpi DCALK1 594-20x1-1.tif.╥╤╙├frames/3dpi DCALK1 594-20x1-1_C001T001.tif]

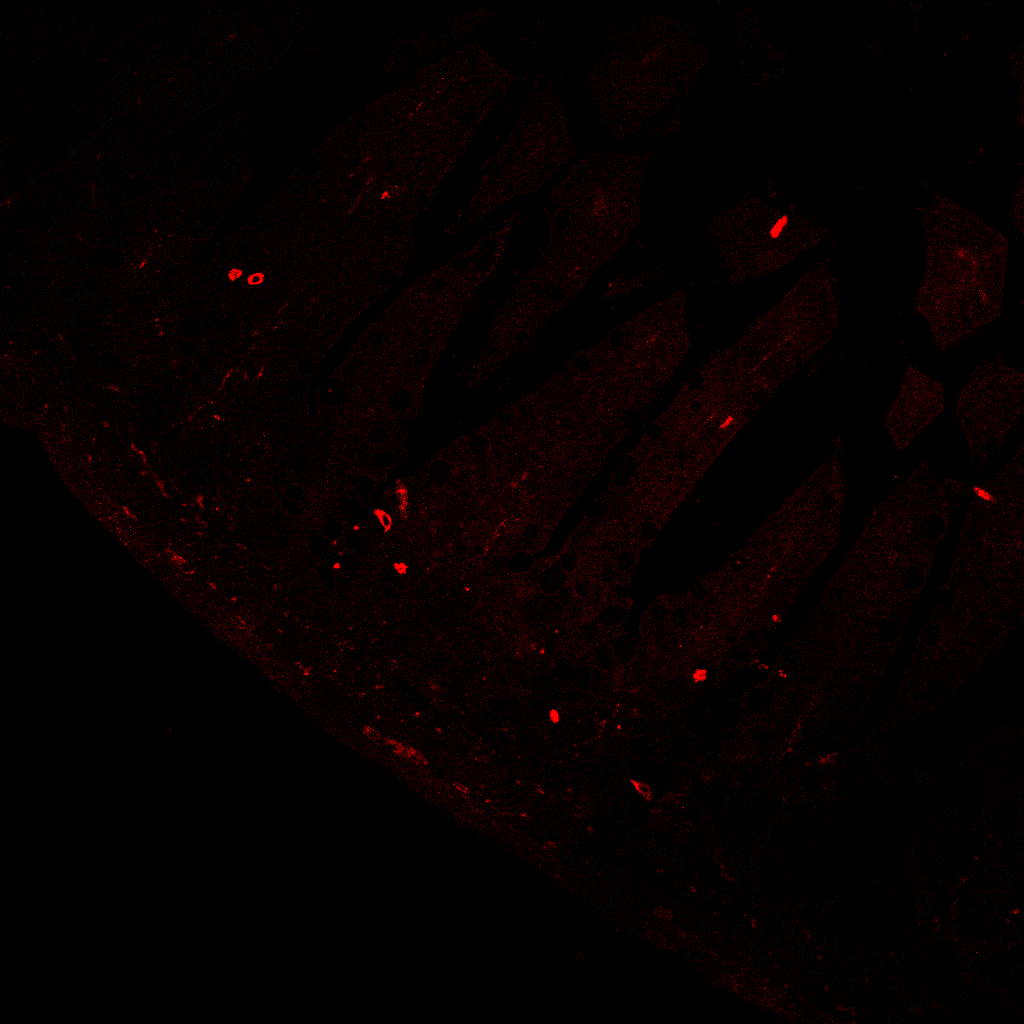

Supplement: Supplementary file 3 — Source data Fig. 1 [file 44321_2024_128_MOESM3_ESM.zip › EMM-2023-19008-V2-figure 1/EMM-2023-19008-V2-figure 1/figure 1D IF/PBS/3dpi/PBS 3dpi DCALK1 594-20x1-1.tif.╥╤╙├frames/3dpi DCALK1 594-20x1-1_C002T001.tif]

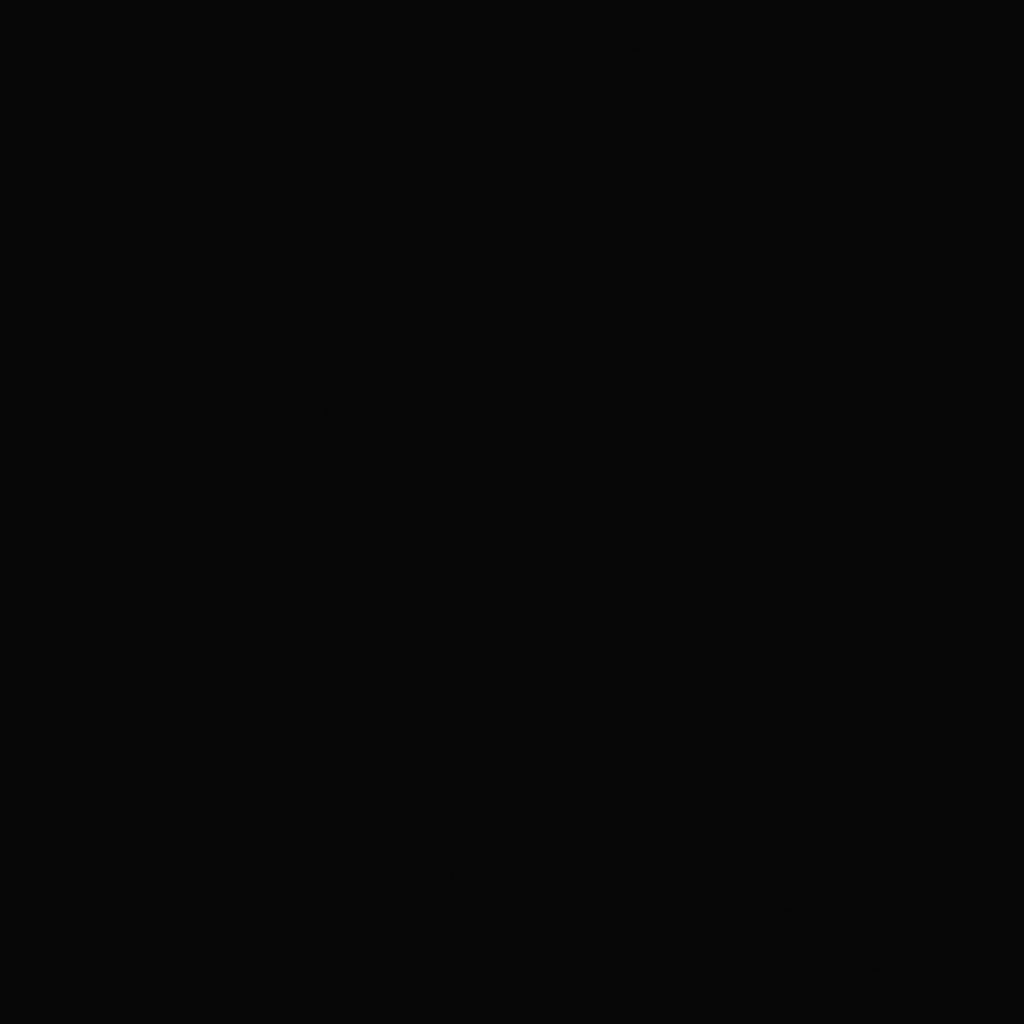

Supplement: Supplementary file 3 — Source data Fig. 1 [file 44321_2024_128_MOESM3_ESM.zip › EMM-2023-19008-V2-figure 1/EMM-2023-19008-V2-figure 1/figure 1D IF/PBS/3dpi/PBS 3dpi DCALK1 594-20x1-1.tif.╥╤╙├frames/3dpi DCALK1 594-20x1-1_C003T001.tif]

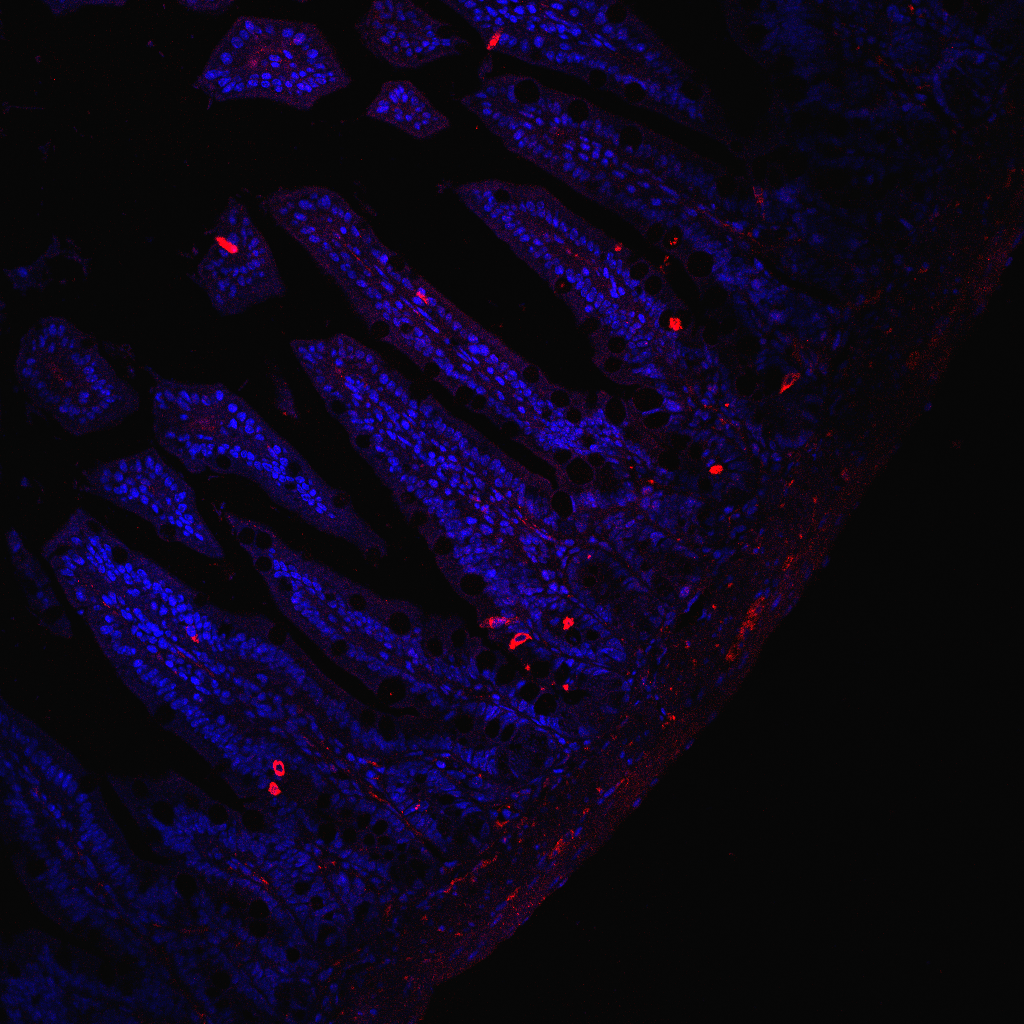

Supplement: Supplementary file 3 — Source data Fig. 1 [file 44321_2024_128_MOESM3_ESM.zip › EMM-2023-19008-V2-figure 1/EMM-2023-19008-V2-figure 1/figure 1D IF/PBS/3dpi/PBS 3dpi DCALK1 594-20x1-1.tif.╥╤╙├frames/3dpi DCALK1 594-20x1-1_T001.tif]

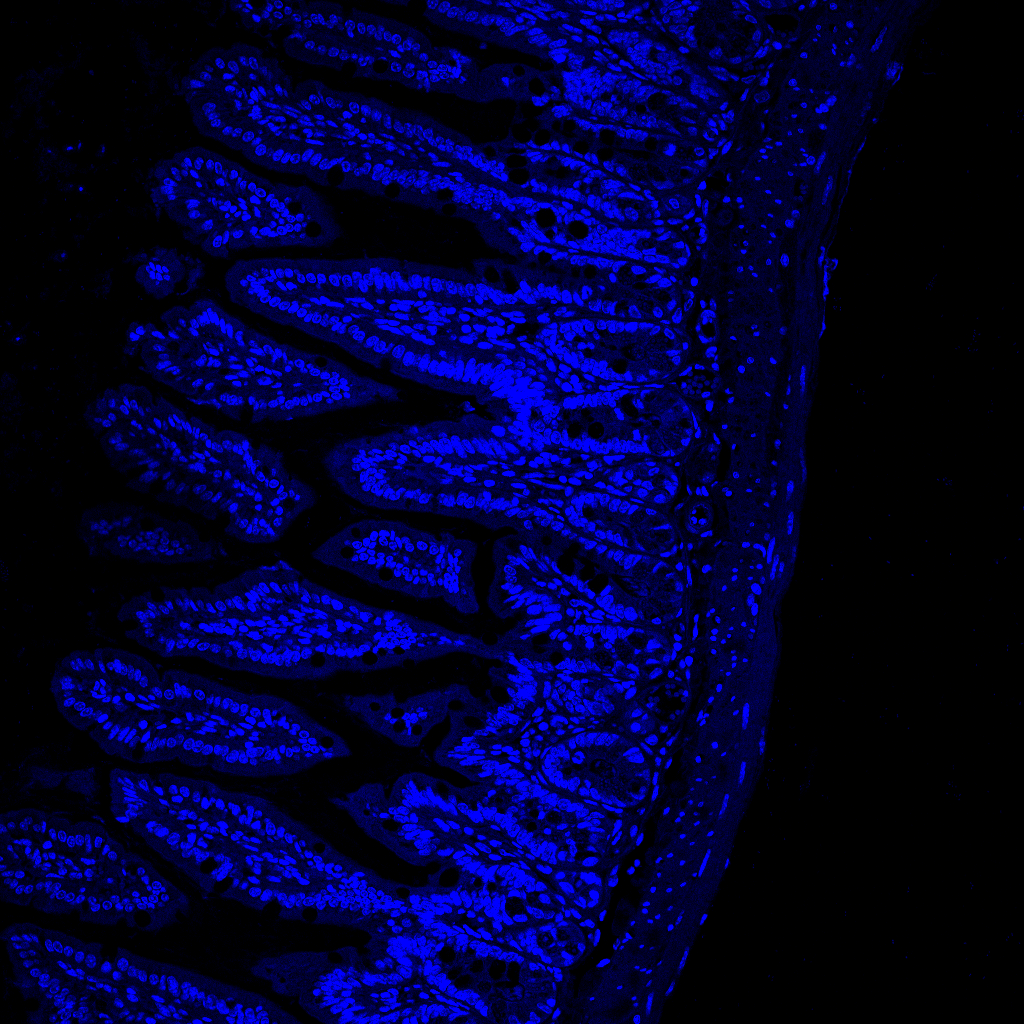

Supplement: Supplementary file 3 — Source data Fig. 1 [file 44321_2024_128_MOESM3_ESM.zip › EMM-2023-19008-V2-figure 1/EMM-2023-19008-V2-figure 1/figure 1D IF/PBS/5dpi/PBS 5dpi DCALK1 594-20x1-3.tif.╥╤╙├frames/2 IL-25 0 ug DCALK1 594-20x1-3_C001T001.tif]

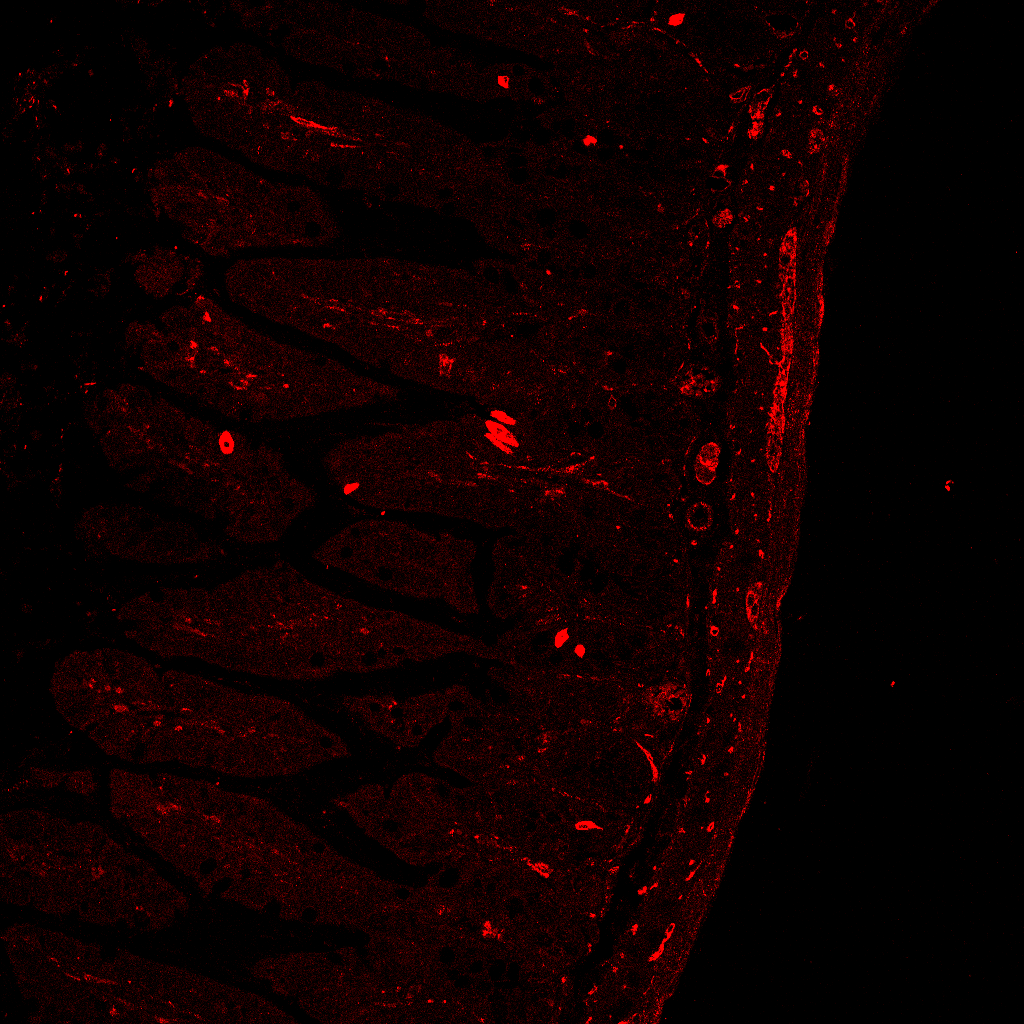

Supplement: Supplementary file 3 — Source data Fig. 1 [file 44321_2024_128_MOESM3_ESM.zip › EMM-2023-19008-V2-figure 1/EMM-2023-19008-V2-figure 1/figure 1D IF/PBS/5dpi/PBS 5dpi DCALK1 594-20x1-3.tif.╥╤╙├frames/2 IL-25 0 ug DCALK1 594-20x1-3_C002T001.tif]

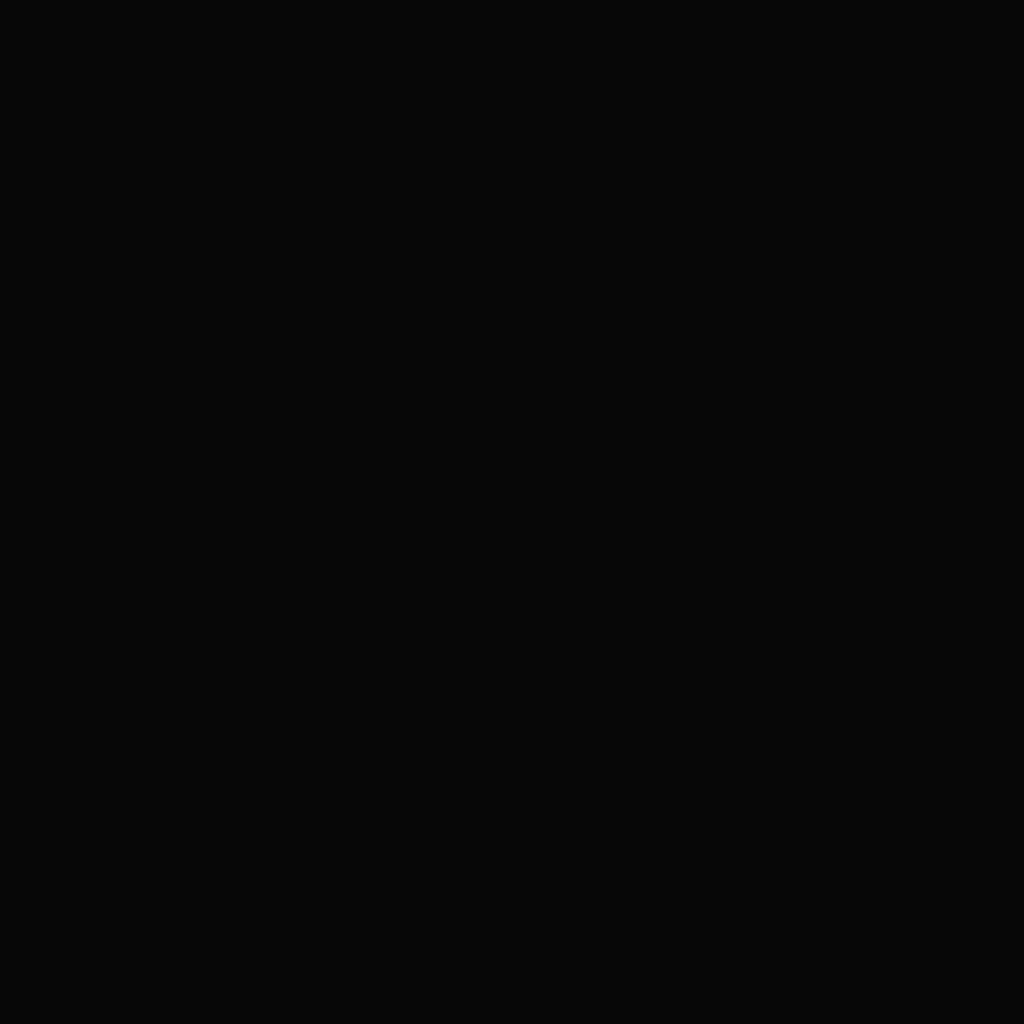

Supplement: Supplementary file 3 — Source data Fig. 1 [file 44321_2024_128_MOESM3_ESM.zip › EMM-2023-19008-V2-figure 1/EMM-2023-19008-V2-figure 1/figure 1D IF/PBS/5dpi/PBS 5dpi DCALK1 594-20x1-3.tif.╥╤╙├frames/2 IL-25 0 ug DCALK1 594-20x1-3_C003T001.tif]

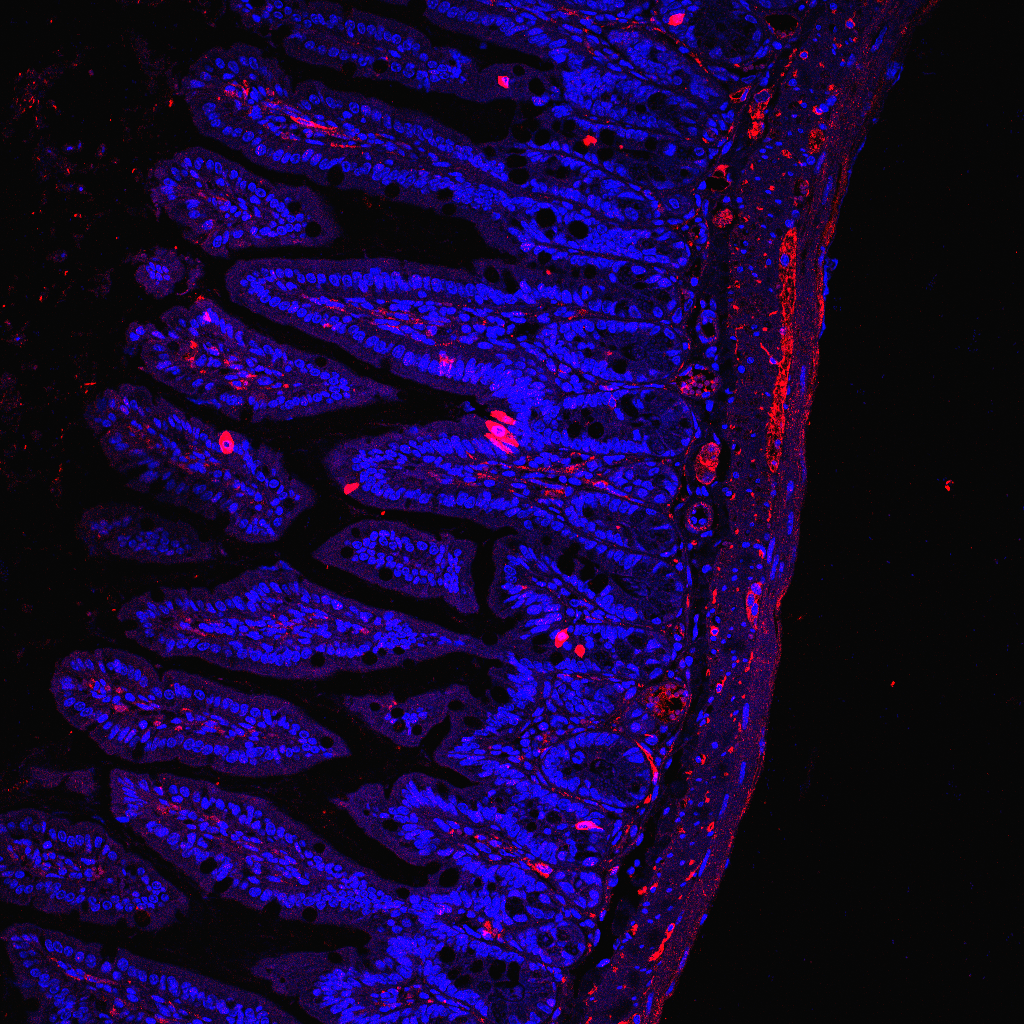

Supplement: Supplementary file 3 — Source data Fig. 1 [file 44321_2024_128_MOESM3_ESM.zip › EMM-2023-19008-V2-figure 1/EMM-2023-19008-V2-figure 1/figure 1D IF/PBS/5dpi/PBS 5dpi DCALK1 594-20x1-3.tif.╥╤╙├frames/2 IL-25 0 ug DCALK1 594-20x1-3_T001.tif]

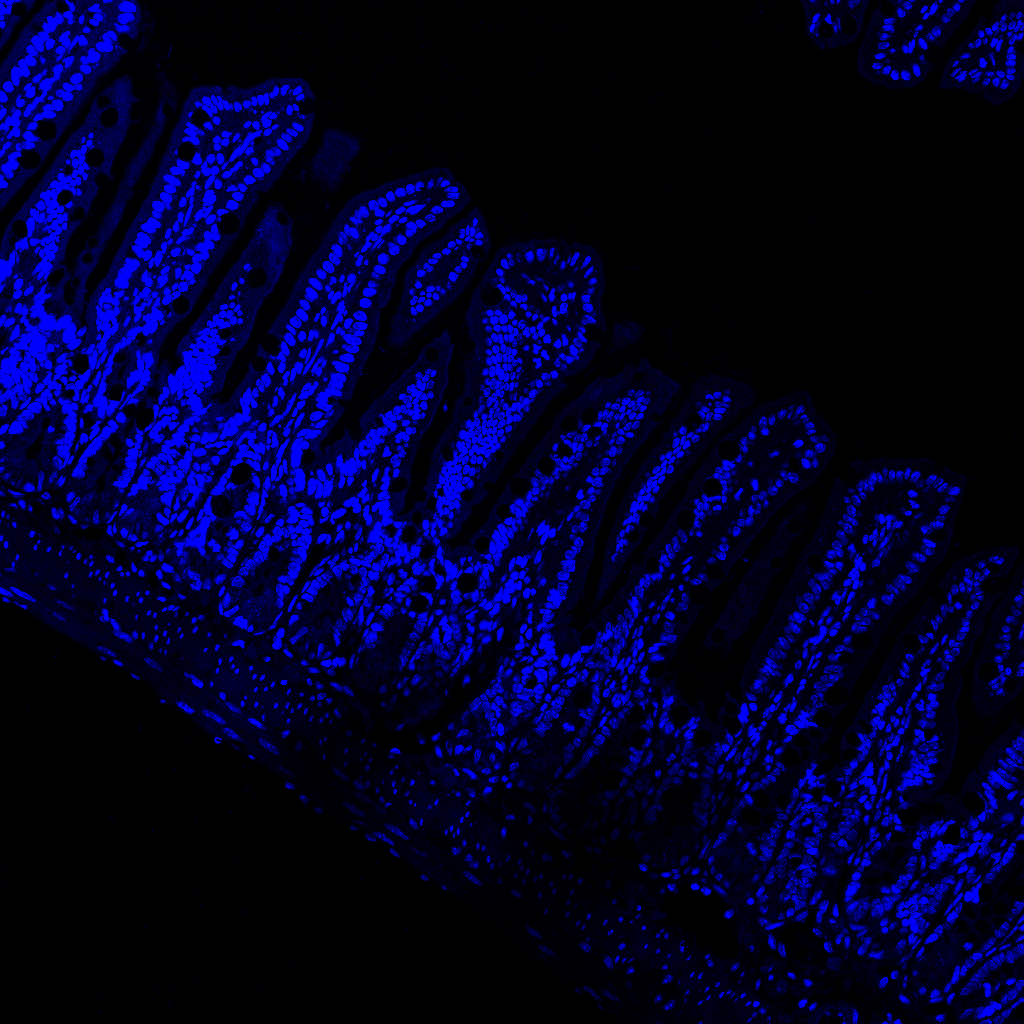

Supplement: Supplementary file 3 — Source data Fig. 1 [file 44321_2024_128_MOESM3_ESM.zip › EMM-2023-19008-V2-figure 1/EMM-2023-19008-V2-figure 1/figure 1D IF/PBS/7dpi/PBS 7dpi DCALK1 594-20x1-1.tif.╥╤╙├frames/PBS 7dpi DCALK1 594-20x1-1_C001T001.tif]

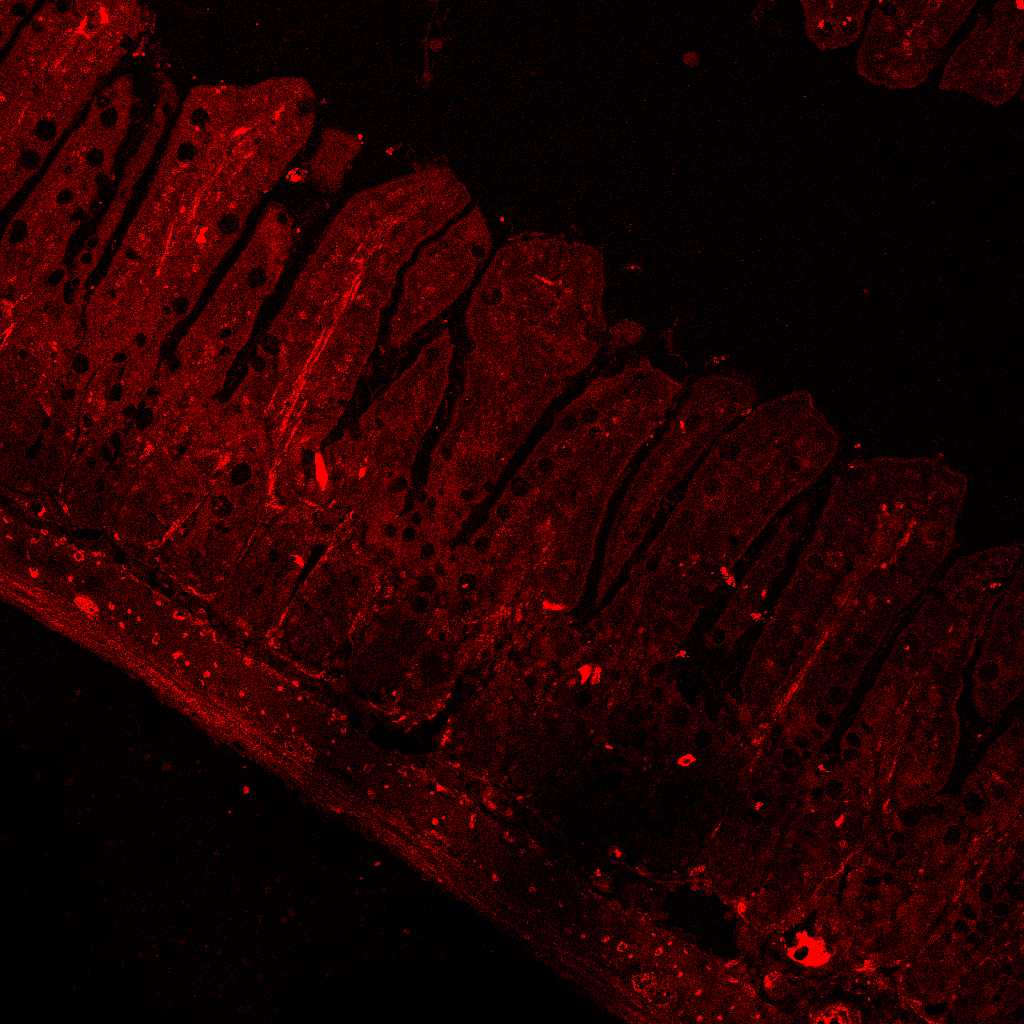

Supplement: Supplementary file 3 — Source data Fig. 1 [file 44321_2024_128_MOESM3_ESM.zip › EMM-2023-19008-V2-figure 1/EMM-2023-19008-V2-figure 1/figure 1D IF/PBS/7dpi/PBS 7dpi DCALK1 594-20x1-1.tif.╥╤╙├frames/PBS 7dpi DCALK1 594-20x1-1_C002T001.tif]

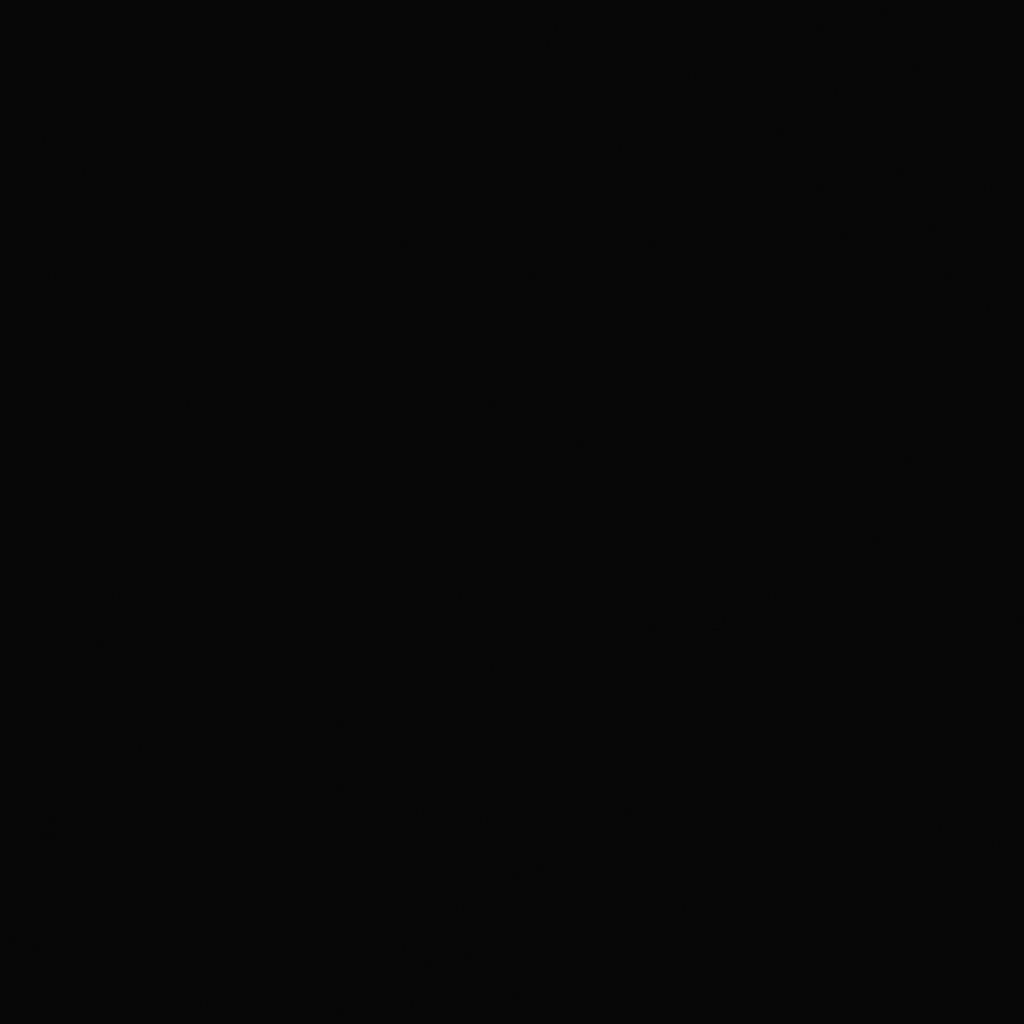

Supplement: Supplementary file 3 — Source data Fig. 1 [file 44321_2024_128_MOESM3_ESM.zip › EMM-2023-19008-V2-figure 1/EMM-2023-19008-V2-figure 1/figure 1D IF/PBS/7dpi/PBS 7dpi DCALK1 594-20x1-1.tif.╥╤╙├frames/PBS 7dpi DCALK1 594-20x1-1_C003T001.tif]

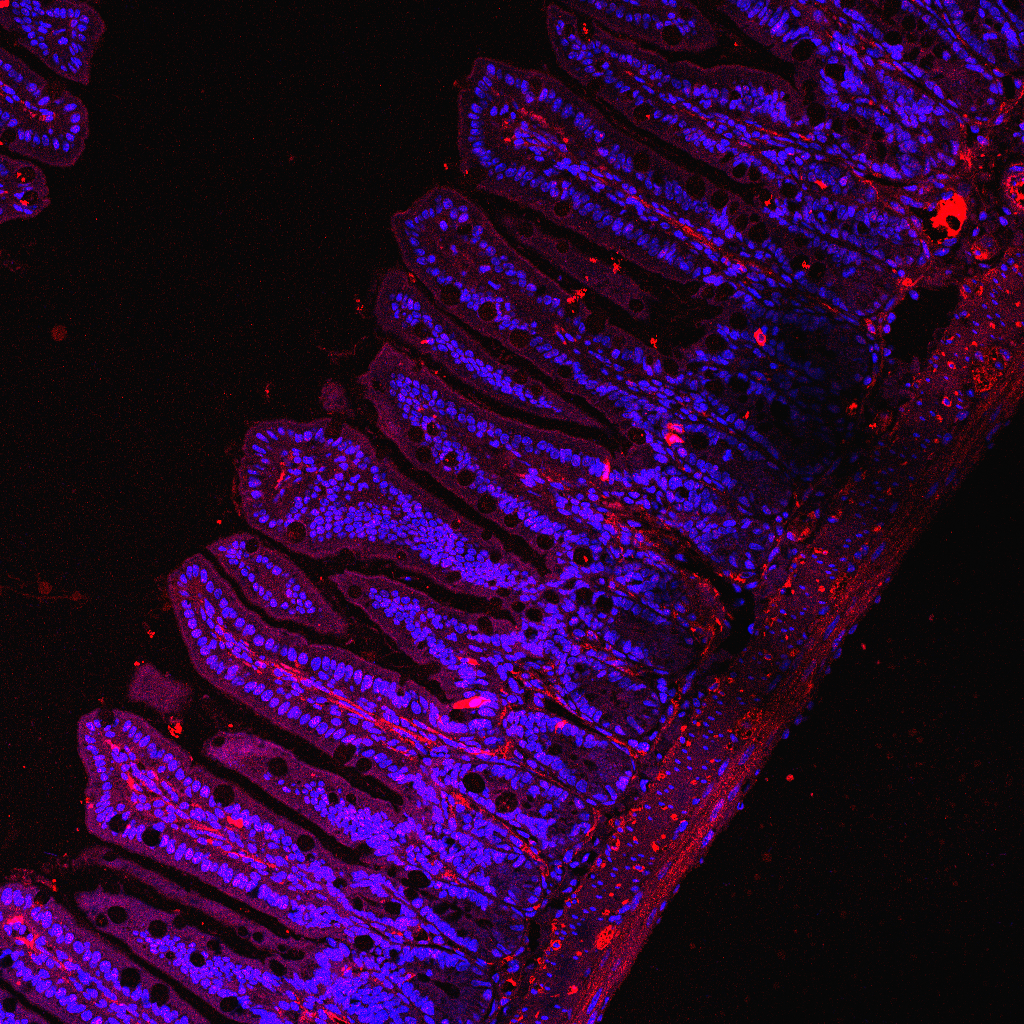

Supplement: Supplementary file 3 — Source data Fig. 1 [file 44321_2024_128_MOESM3_ESM.zip › EMM-2023-19008-V2-figure 1/EMM-2023-19008-V2-figure 1/figure 1D IF/PBS/7dpi/PBS 7dpi DCALK1 594-20x1-1.tif.╥╤╙├frames/PBS 7dpi DCALK1 594-20x1-1_T001.tif]

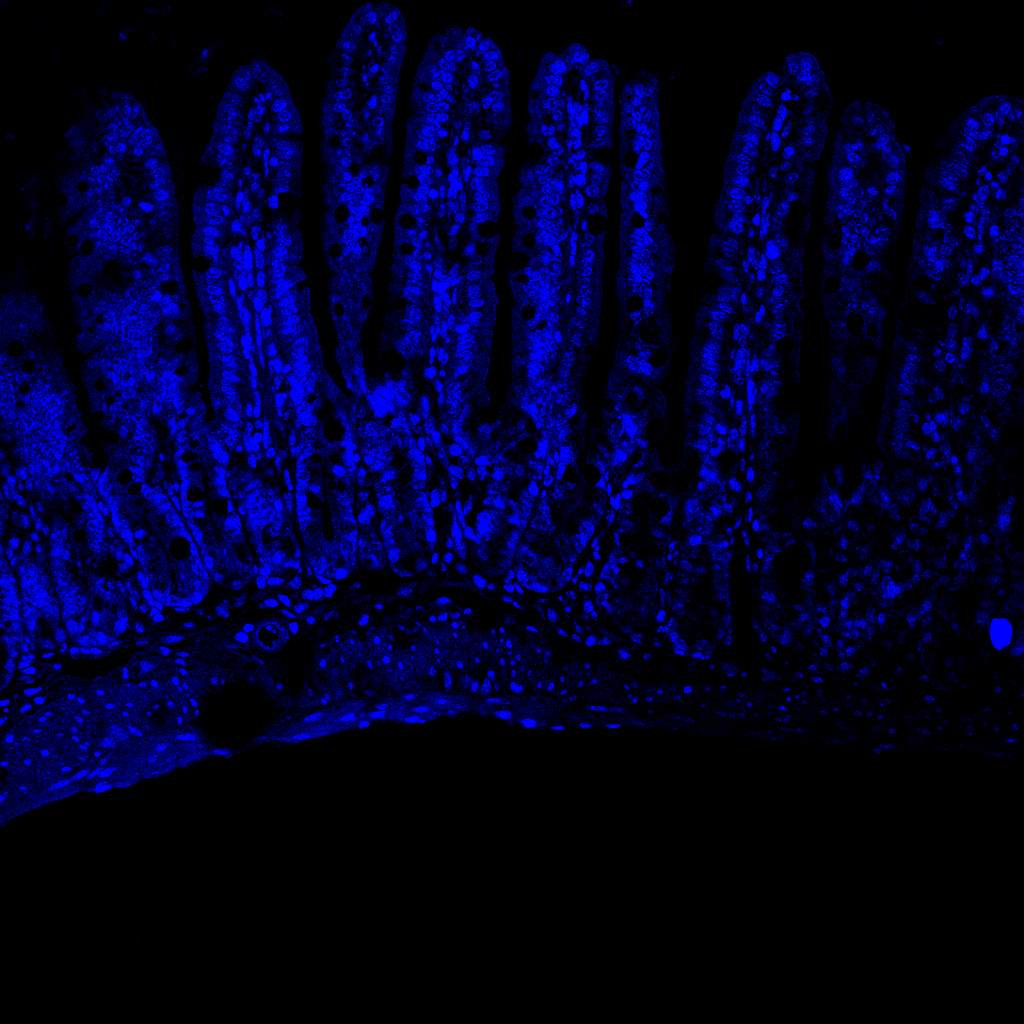

Supplement: Supplementary file 3 — Source data Fig. 1 [file 44321_2024_128_MOESM3_ESM.zip › EMM-2023-19008-V2-figure 1/EMM-2023-19008-V2-figure 1/figure 1F IF/C57 01 ileum DCLK2594 TRPM5488-20x1-3.tif.frames ╥╤╙├/C57 01 ileum DCLK2594 TRPM5488-20x1-3_C001T001.tif]

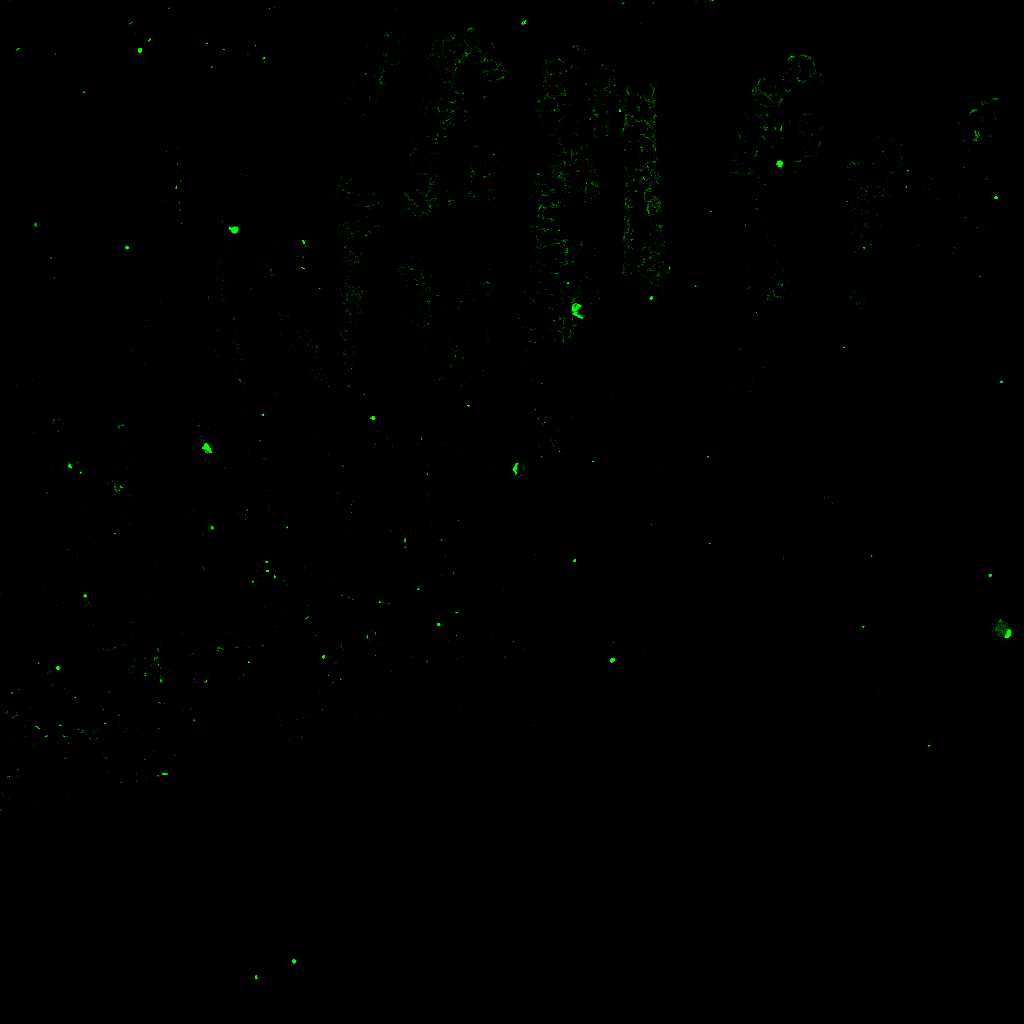

Supplement: Supplementary file 3 — Source data Fig. 1 [file 44321_2024_128_MOESM3_ESM.zip › EMM-2023-19008-V2-figure 1/EMM-2023-19008-V2-figure 1/figure 1F IF/C57 01 ileum DCLK2594 TRPM5488-20x1-3.tif.frames ╥╤╙├/C57 01 ileum DCLK2594 TRPM5488-20x1-3_C002T001.tif]

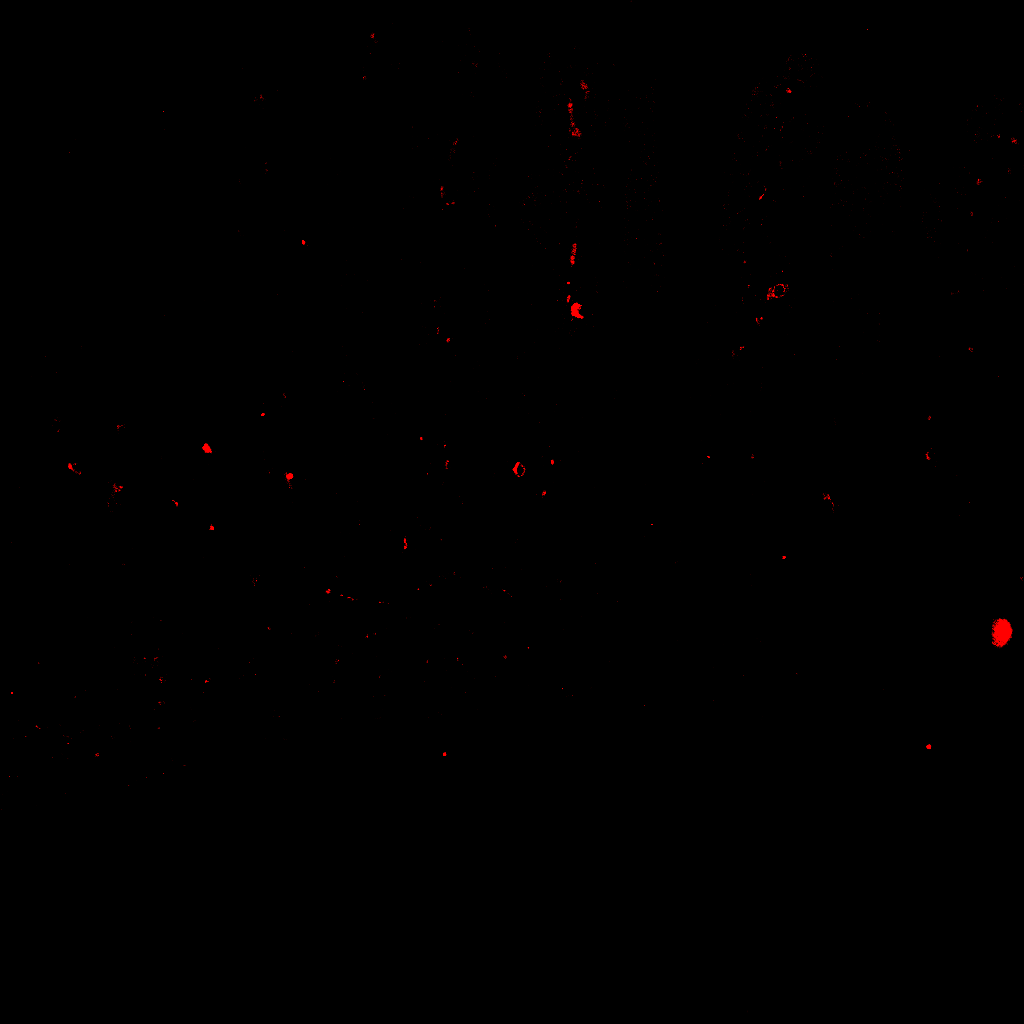

Supplement: Supplementary file 3 — Source data Fig. 1 [file 44321_2024_128_MOESM3_ESM.zip › EMM-2023-19008-V2-figure 1/EMM-2023-19008-V2-figure 1/figure 1F IF/C57 01 ileum DCLK2594 TRPM5488-20x1-3.tif.frames ╥╤╙├/C57 01 ileum DCLK2594 TRPM5488-20x1-3_C003T001.tif]

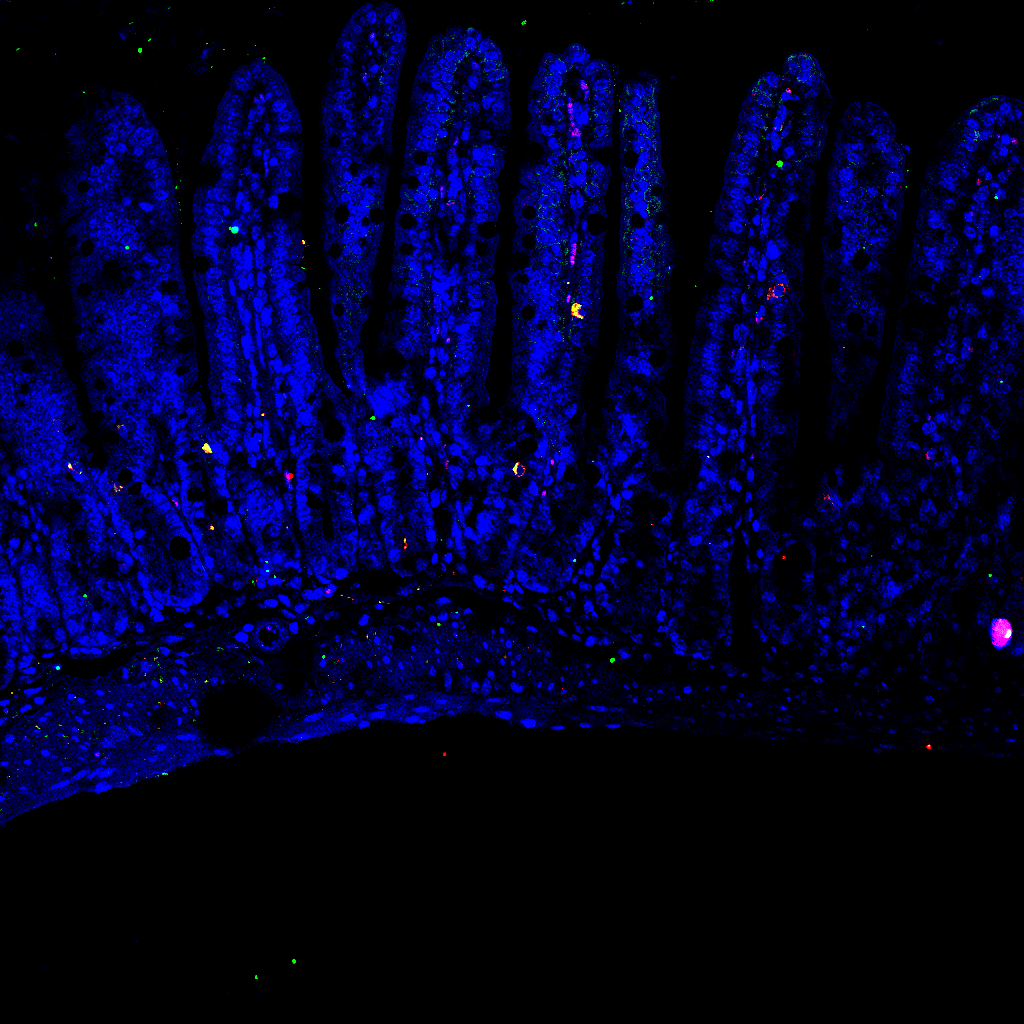

Supplement: Supplementary file 3 — Source data Fig. 1 [file 44321_2024_128_MOESM3_ESM.zip › EMM-2023-19008-V2-figure 1/EMM-2023-19008-V2-figure 1/figure 1F IF/C57 01 ileum DCLK2594 TRPM5488-20x1-3.tif.frames ╥╤╙├/C57 01 ileum DCLK2594 TRPM5488-20x1-3_T001.tif]

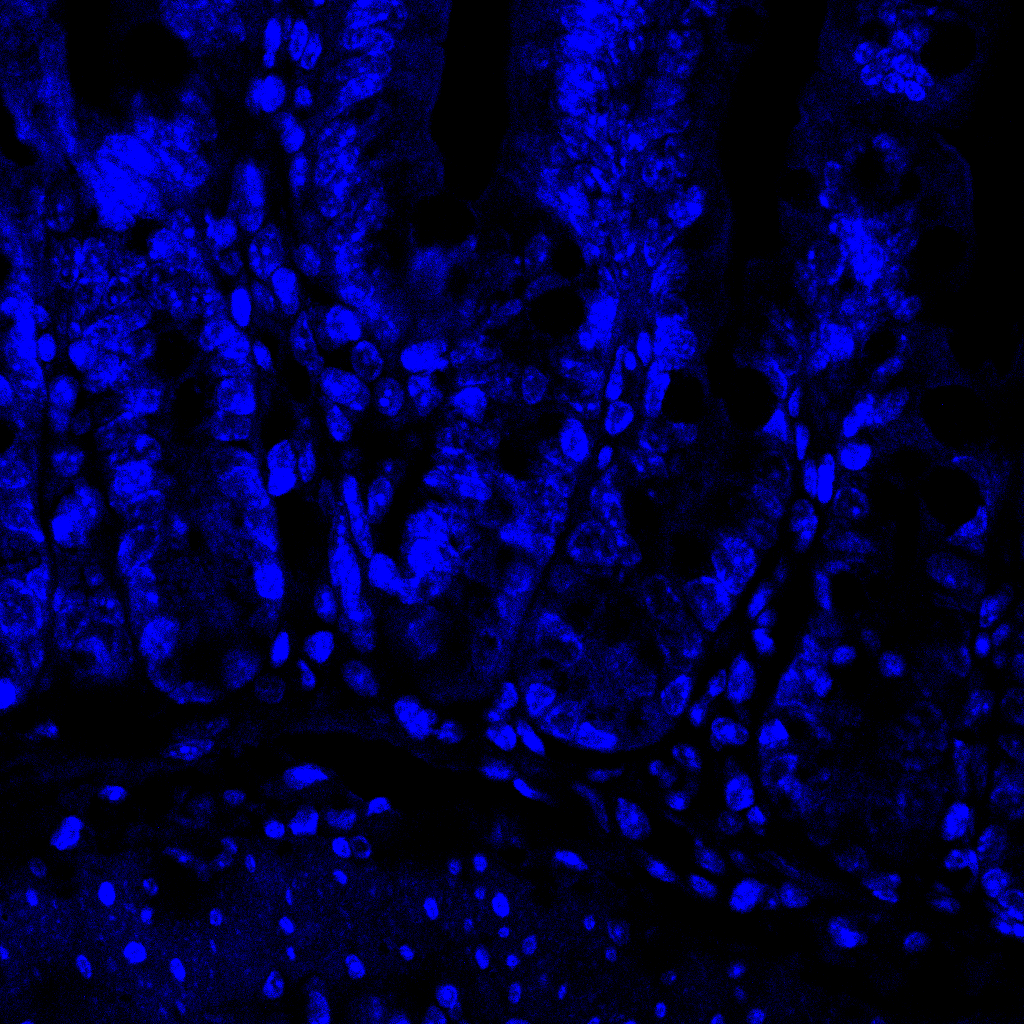

Supplement: Supplementary file 3 — Source data Fig. 1 [file 44321_2024_128_MOESM3_ESM.zip › EMM-2023-19008-V2-figure 1/EMM-2023-19008-V2-figure 1/figure 1F IF/C57 01 ileum DCLK2594 TRPM5488-20x3-3.tif.frames ╥╤╙├/C57 01 ileum DCLK2594 TRPM5488-20x3-3_C001T001.tif]

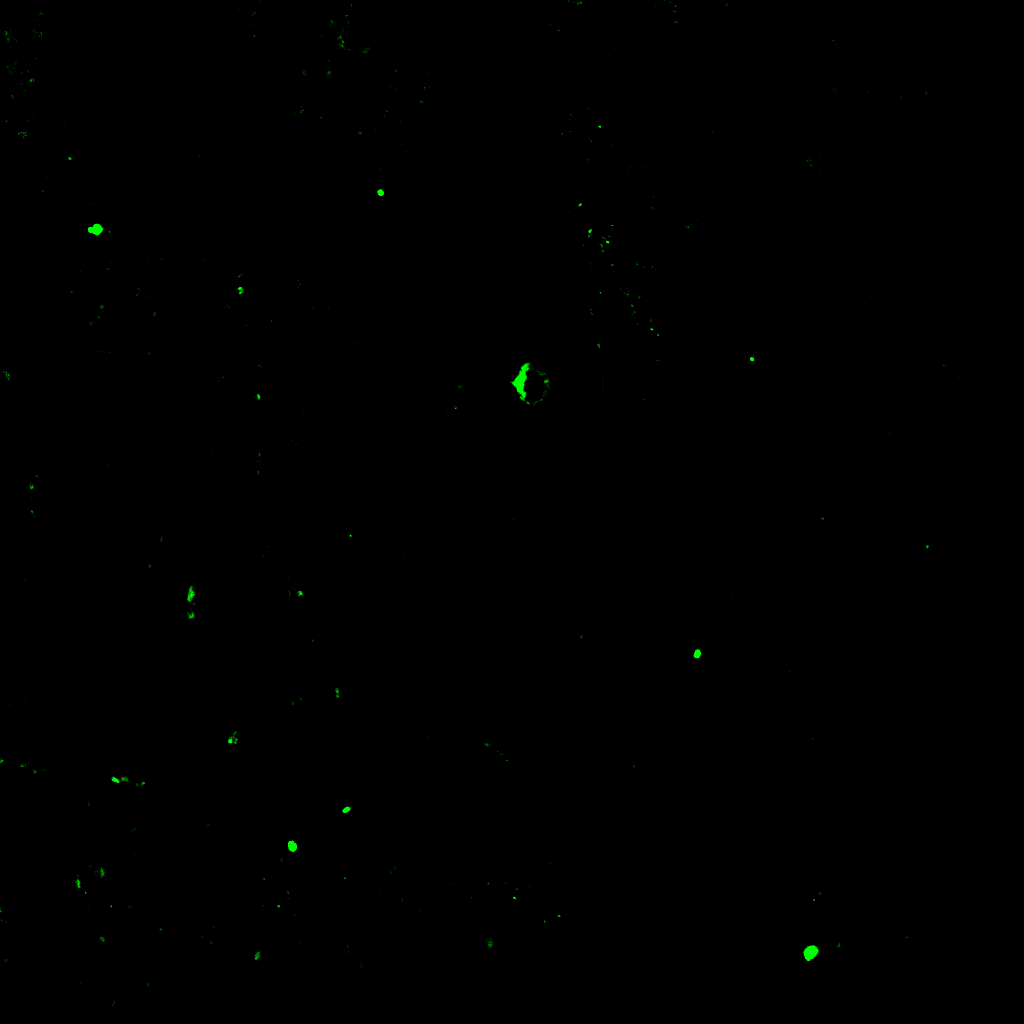

Supplement: Supplementary file 3 — Source data Fig. 1 [file 44321_2024_128_MOESM3_ESM.zip › EMM-2023-19008-V2-figure 1/EMM-2023-19008-V2-figure 1/figure 1F IF/C57 01 ileum DCLK2594 TRPM5488-20x3-3.tif.frames ╥╤╙├/C57 01 ileum DCLK2594 TRPM5488-20x3-3_C002T001.tif]

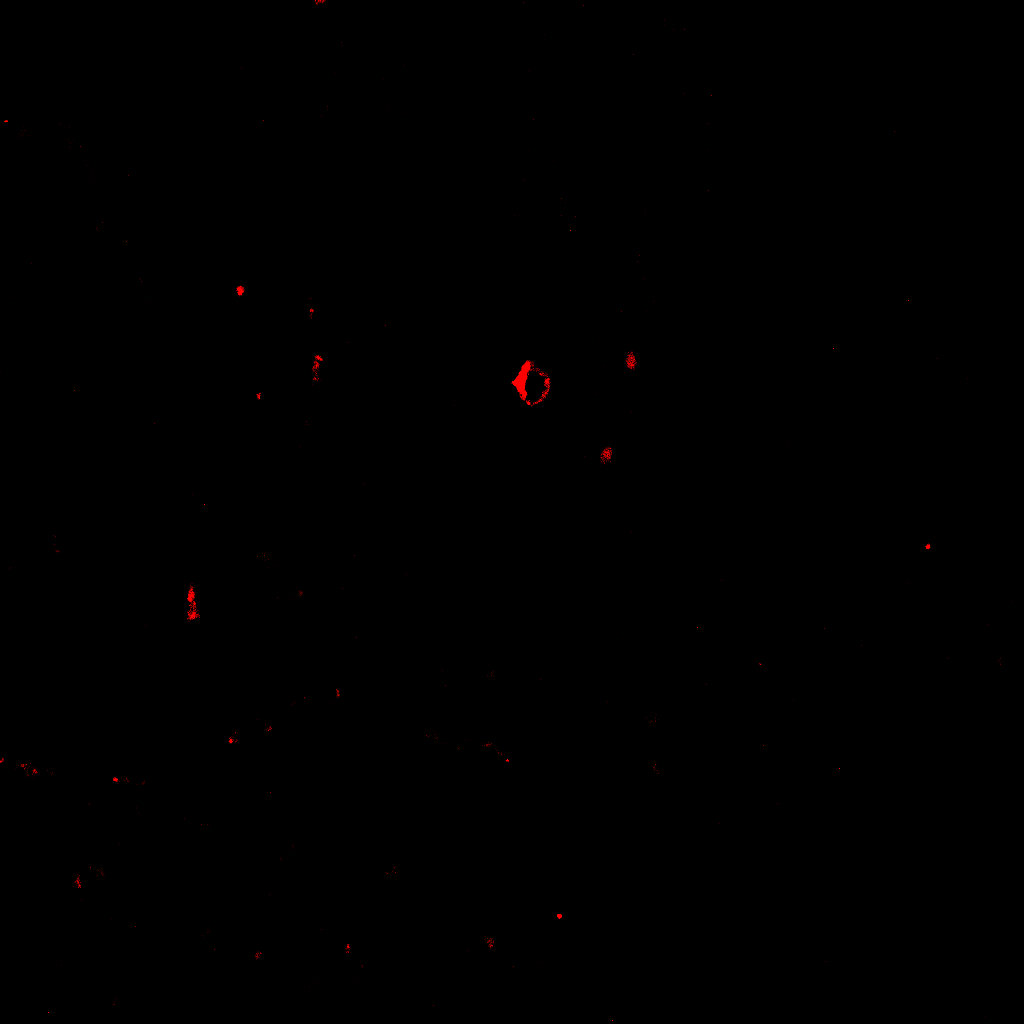

Supplement: Supplementary file 3 — Source data Fig. 1 [file 44321_2024_128_MOESM3_ESM.zip › EMM-2023-19008-V2-figure 1/EMM-2023-19008-V2-figure 1/figure 1F IF/C57 01 ileum DCLK2594 TRPM5488-20x3-3.tif.frames ╥╤╙├/C57 01 ileum DCLK2594 TRPM5488-20x3-3_C003T001.tif]

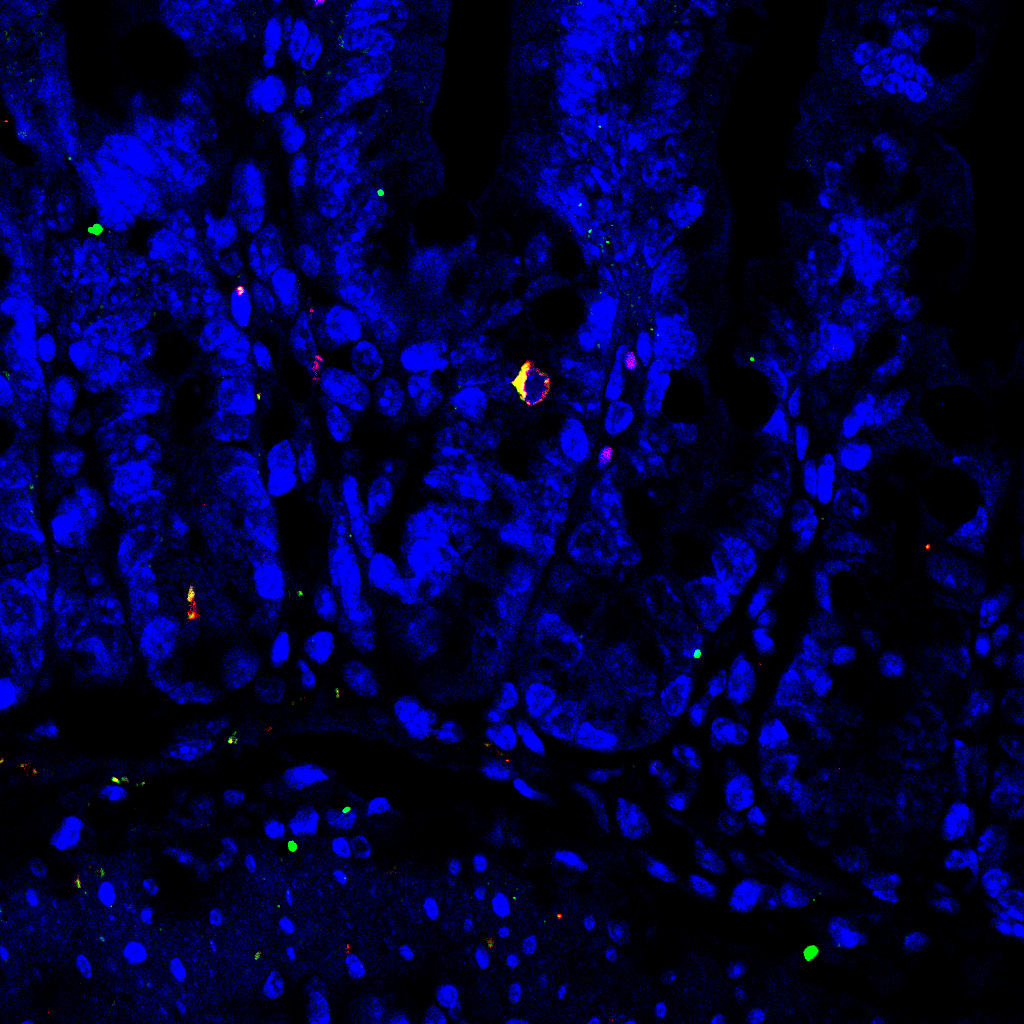

Supplement: Supplementary file 3 — Source data Fig. 1 [file 44321_2024_128_MOESM3_ESM.zip › EMM-2023-19008-V2-figure 1/EMM-2023-19008-V2-figure 1/figure 1F IF/C57 01 ileum DCLK2594 TRPM5488-20x3-3.tif.frames ╥╤╙├/C57 01 ileum DCLK2594 TRPM5488-20x3-3_T001.tif]

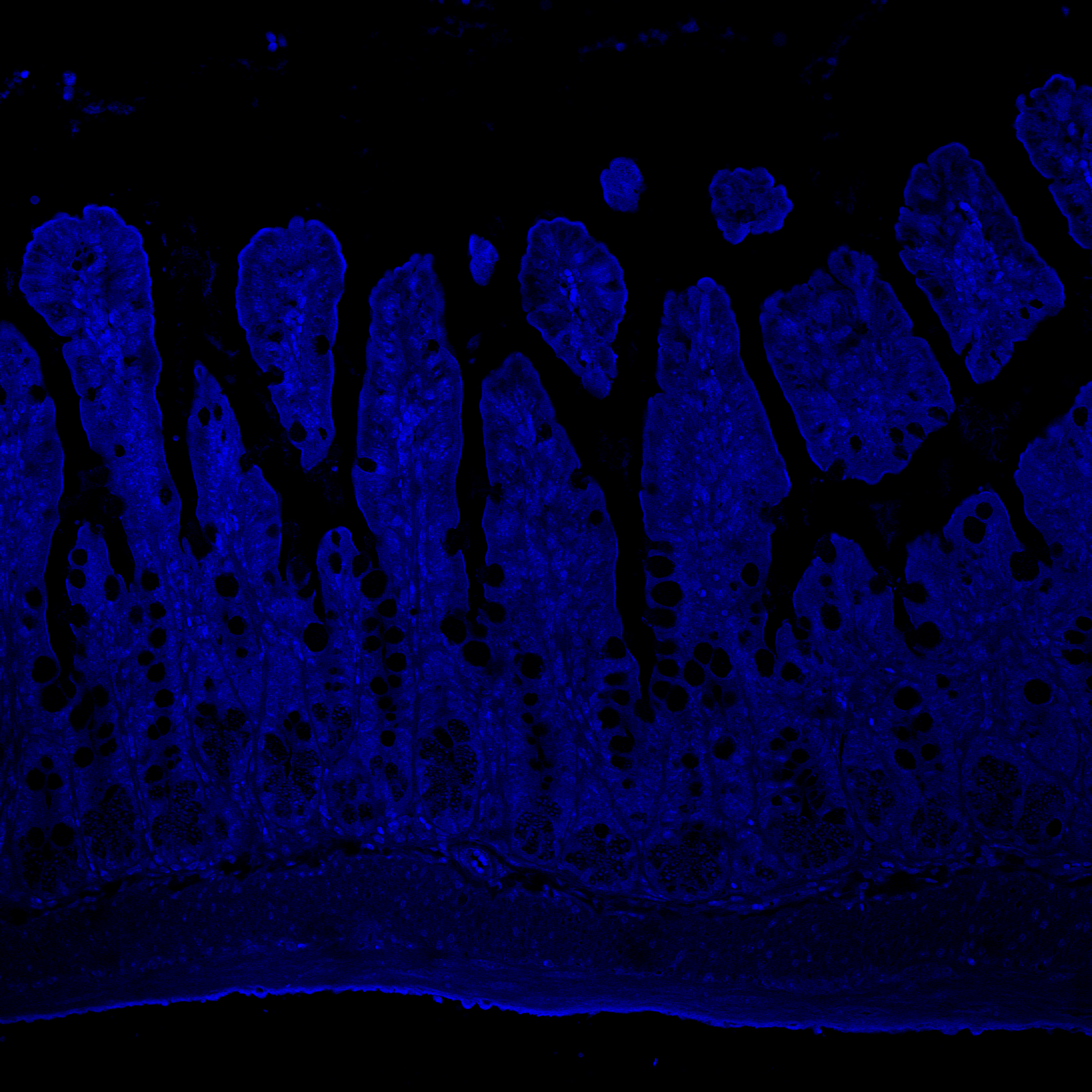

Supplement: Supplementary file 3 — Source data Fig. 1 [file 44321_2024_128_MOESM3_ESM.zip › EMM-2023-19008-V2-figure 1/EMM-2023-19008-V2-figure 1/figure 1F IF/C57 IL-25 8 DCLK1 594 TRPM5 488-20x1-5.tif.frames ╥╤╙├/C57 IL-25 8 DCLK1 594 TRPM5 488-20x1-5_C001T001.tif]

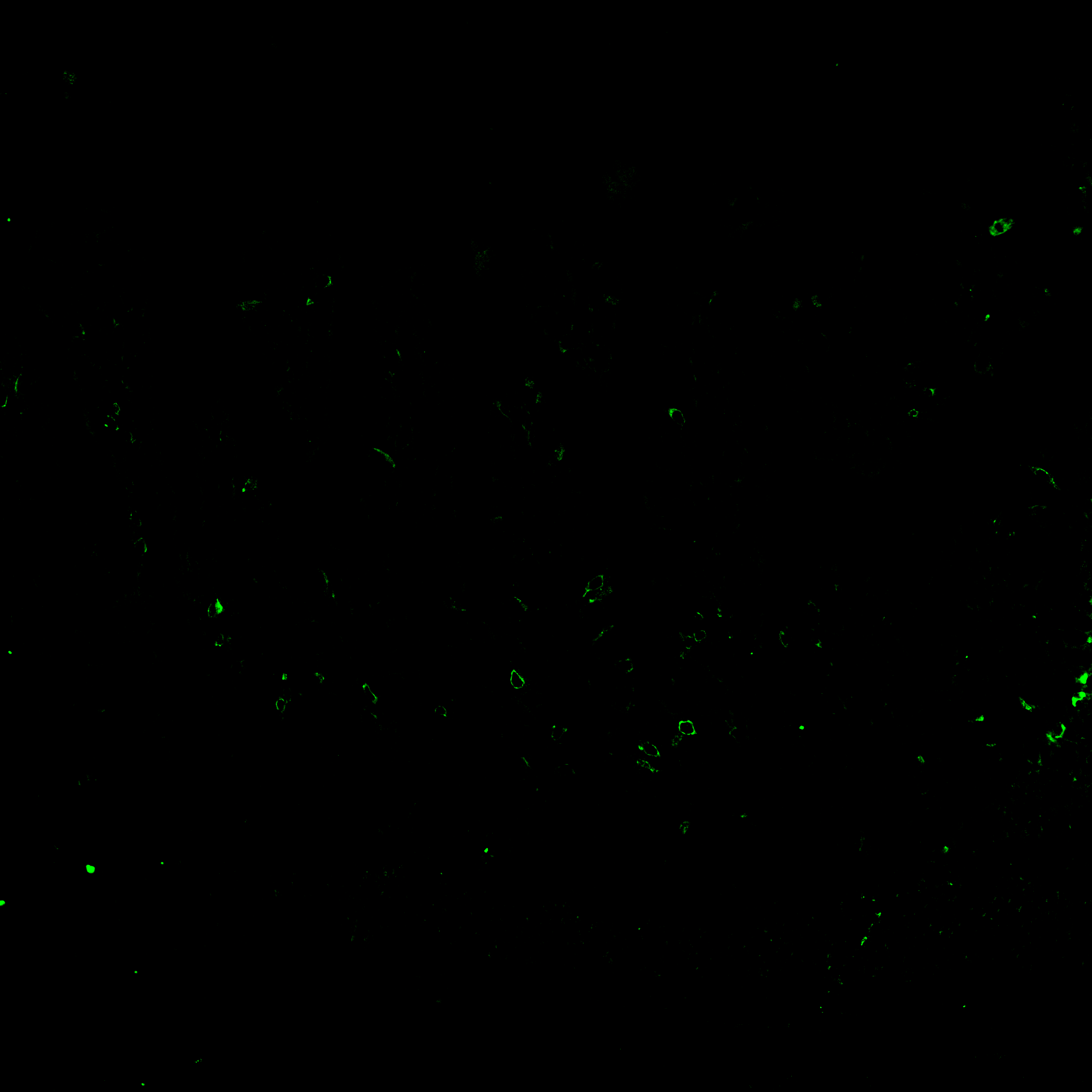

Supplement: Supplementary file 3 — Source data Fig. 1 [file 44321_2024_128_MOESM3_ESM.zip › EMM-2023-19008-V2-figure 1/EMM-2023-19008-V2-figure 1/figure 1F IF/C57 IL-25 8 DCLK1 594 TRPM5 488-20x1-5.tif.frames ╥╤╙├/C57 IL-25 8 DCLK1 594 TRPM5 488-20x1-5_C002T001.tif]

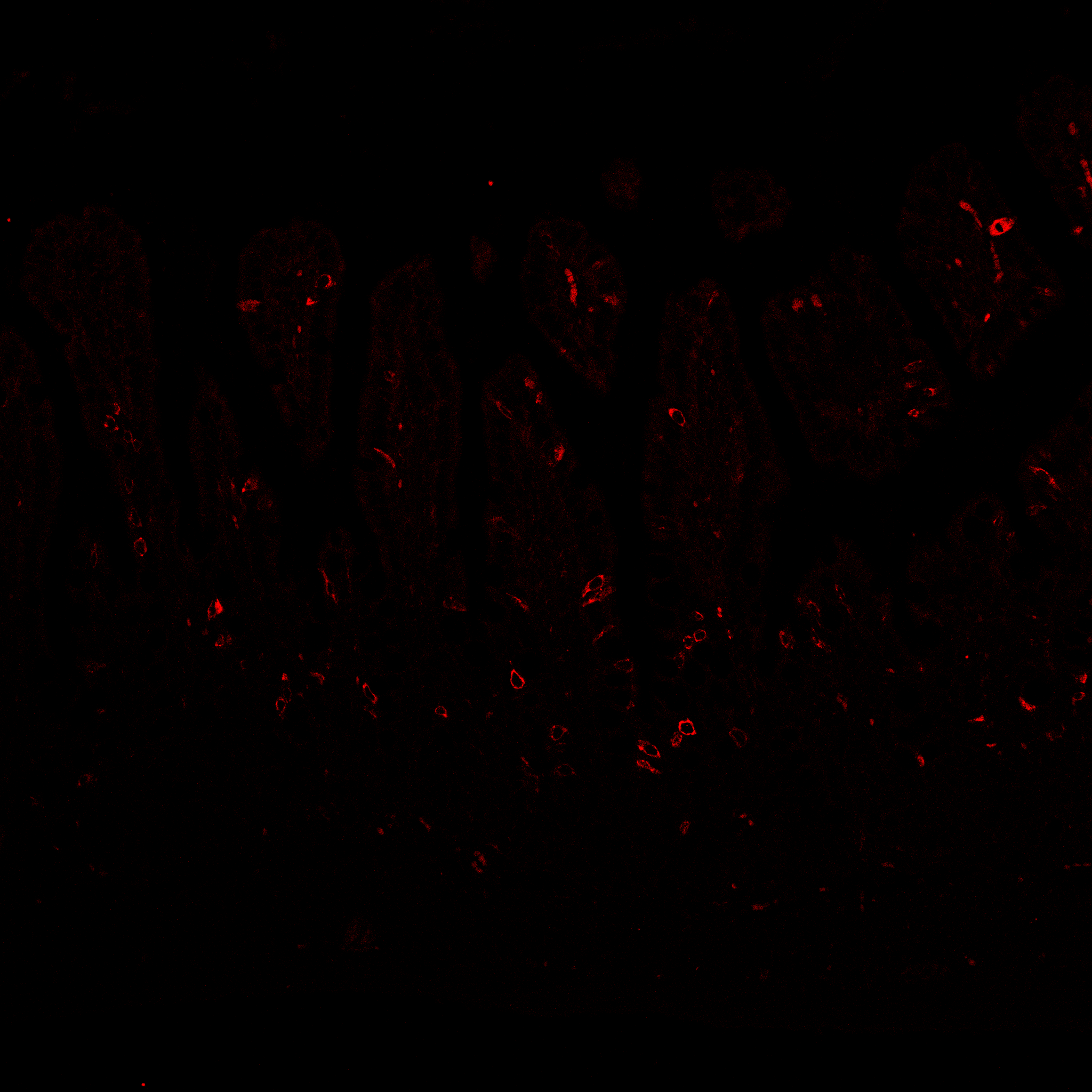

Supplement: Supplementary file 3 — Source data Fig. 1 [file 44321_2024_128_MOESM3_ESM.zip › EMM-2023-19008-V2-figure 1/EMM-2023-19008-V2-figure 1/figure 1F IF/C57 IL-25 8 DCLK1 594 TRPM5 488-20x1-5.tif.frames ╥╤╙├/C57 IL-25 8 DCLK1 594 TRPM5 488-20x1-5_C003T001.tif]

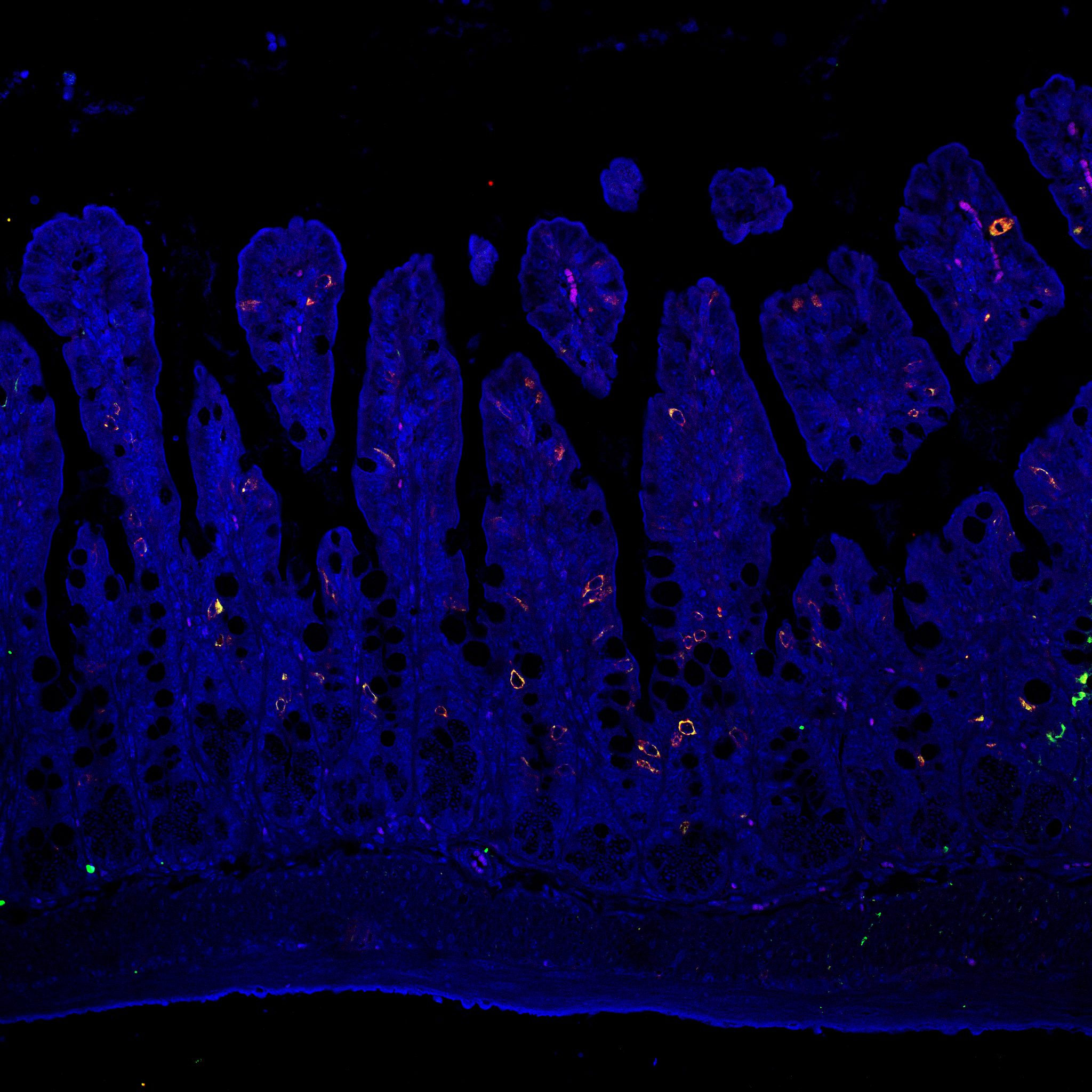

Supplement: Supplementary file 3 — Source data Fig. 1 [file 44321_2024_128_MOESM3_ESM.zip › EMM-2023-19008-V2-figure 1/EMM-2023-19008-V2-figure 1/figure 1F IF/C57 IL-25 8 DCLK1 594 TRPM5 488-20x1-5.tif.frames ╥╤╙├/C57 IL-25 8 DCLK1 594 TRPM5 488-20x1-5_T001.tif]

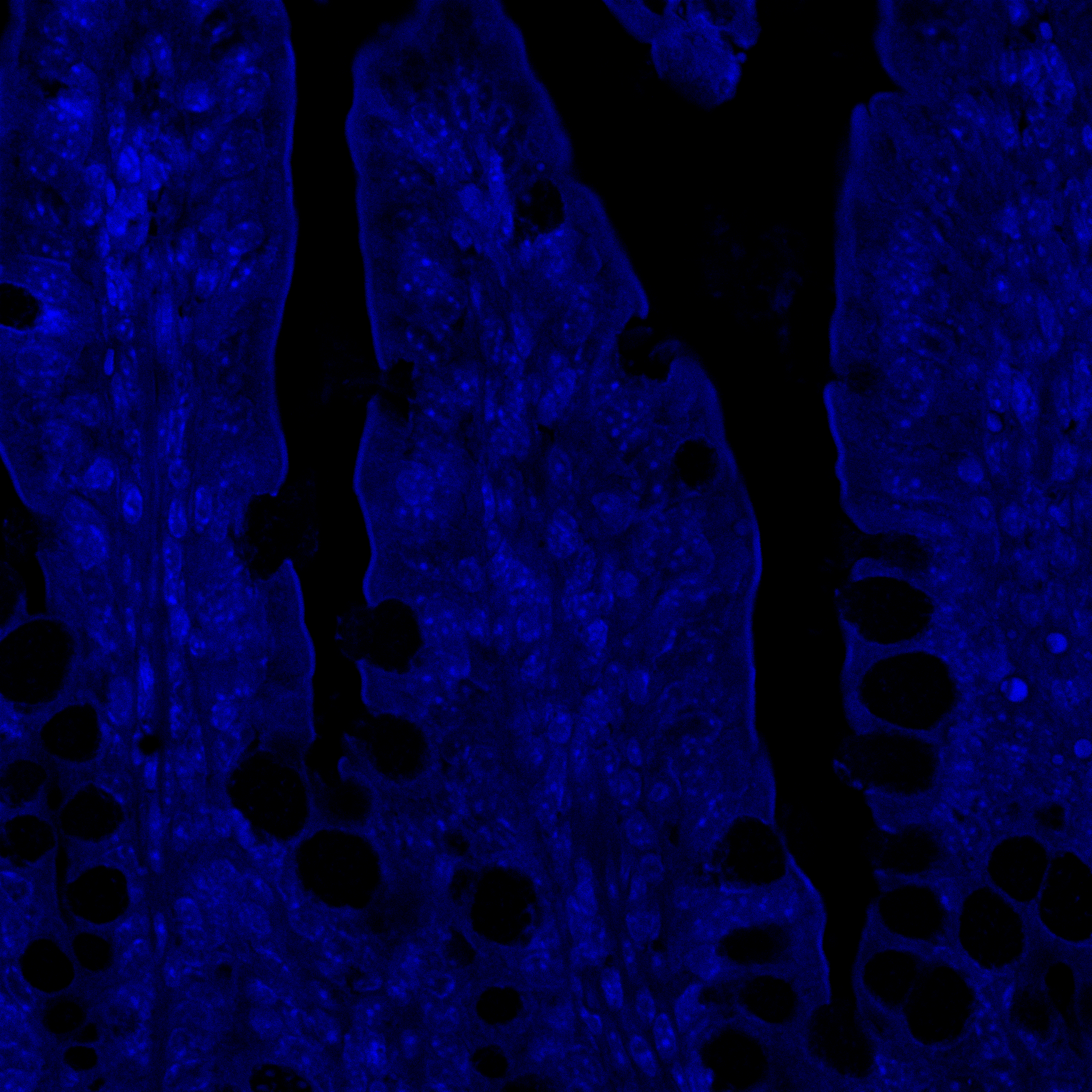

Supplement: Supplementary file 3 — Source data Fig. 1 [file 44321_2024_128_MOESM3_ESM.zip › EMM-2023-19008-V2-figure 1/EMM-2023-19008-V2-figure 1/figure 1F IF/C57 IL-25 8 DCLK1 594 TRPM5 488-20x3-5.tif.frames ╥╤╙├/C57 IL-25 8 DCLK1 594 TRPM5 488-20x3-5_C001T001.tif]

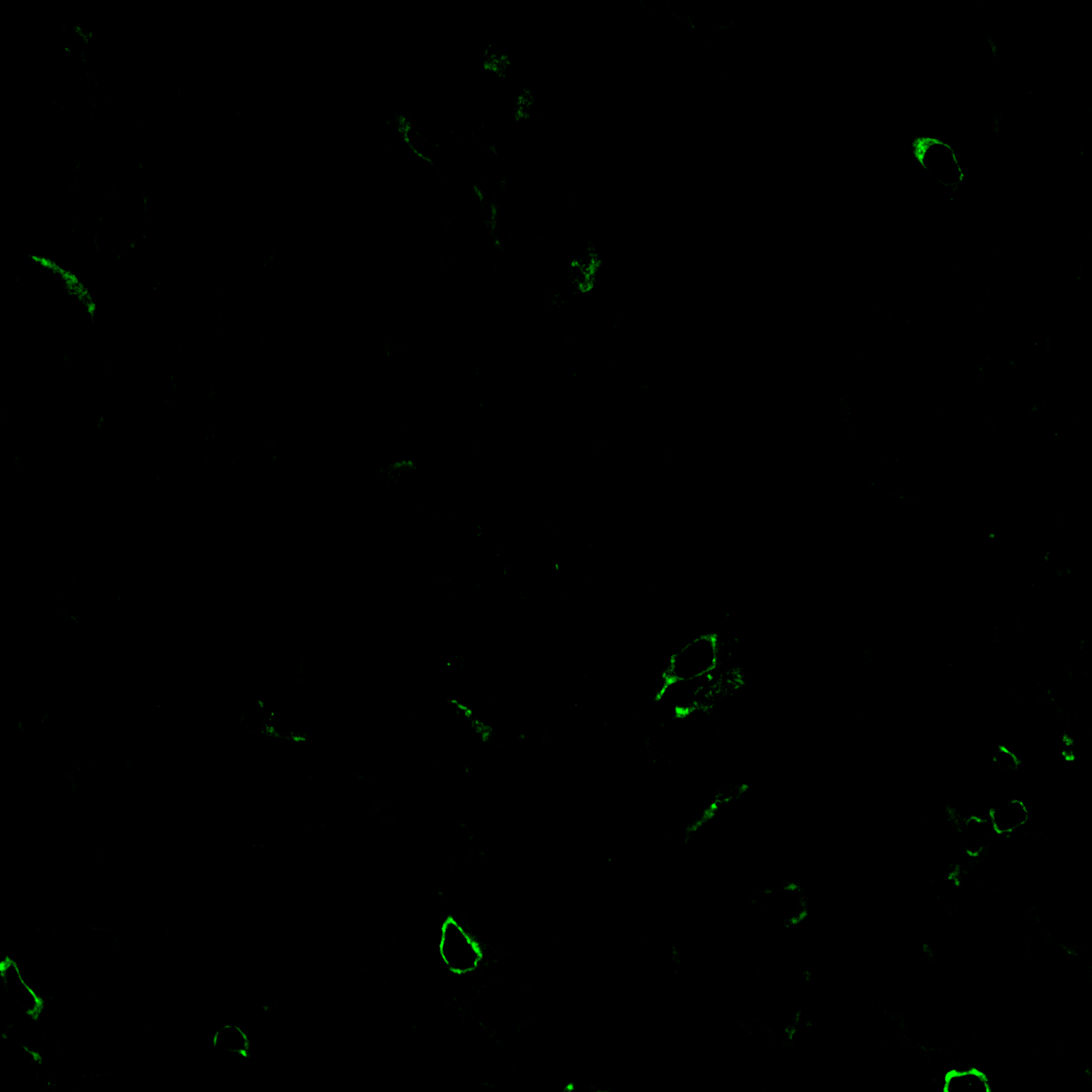

Supplement: Supplementary file 3 — Source data Fig. 1 [file 44321_2024_128_MOESM3_ESM.zip › EMM-2023-19008-V2-figure 1/EMM-2023-19008-V2-figure 1/figure 1F IF/C57 IL-25 8 DCLK1 594 TRPM5 488-20x3-5.tif.frames ╥╤╙├/C57 IL-25 8 DCLK1 594 TRPM5 488-20x3-5_C002T001.tif]

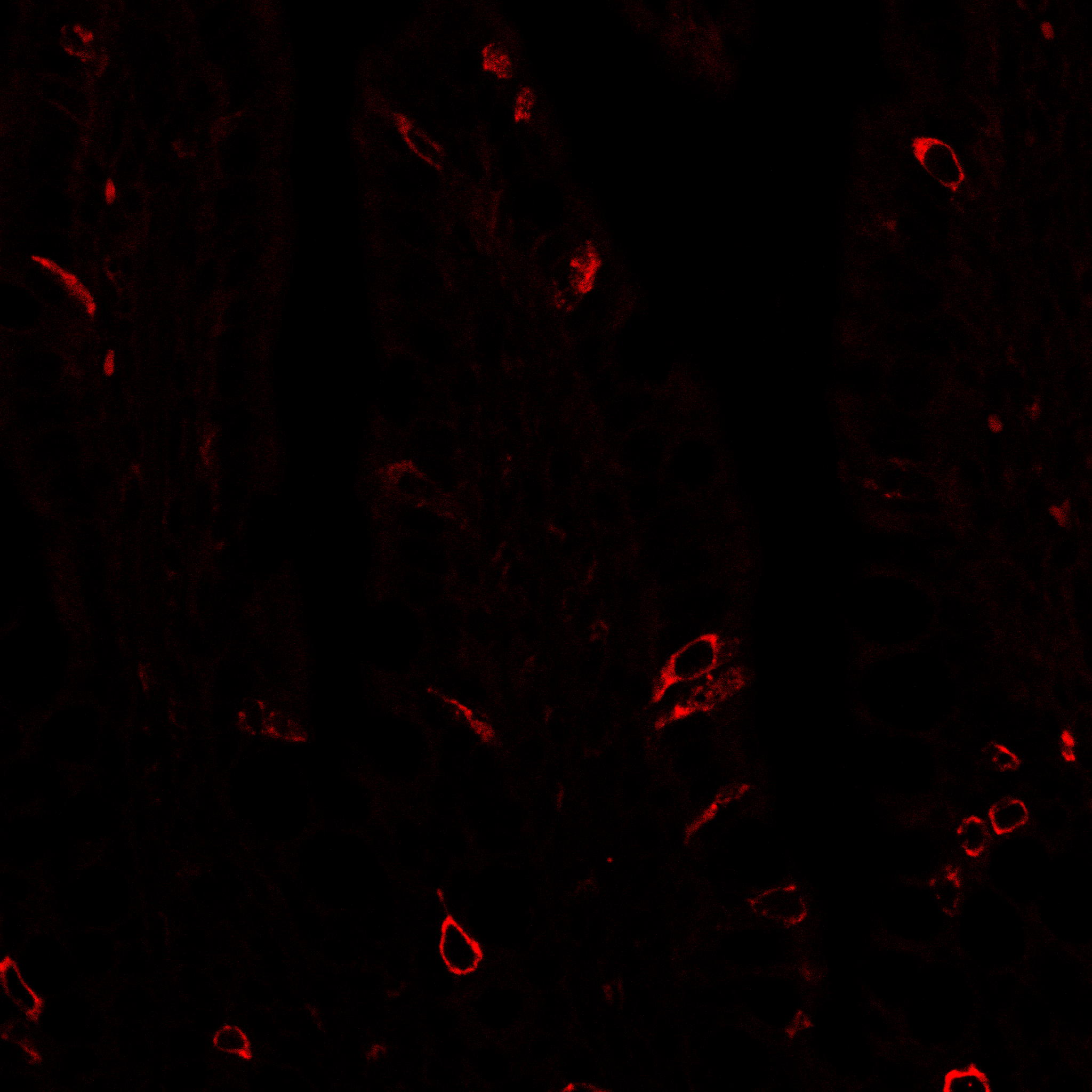

Supplement: Supplementary file 3 — Source data Fig. 1 [file 44321_2024_128_MOESM3_ESM.zip › EMM-2023-19008-V2-figure 1/EMM-2023-19008-V2-figure 1/figure 1F IF/C57 IL-25 8 DCLK1 594 TRPM5 488-20x3-5.tif.frames ╥╤╙├/C57 IL-25 8 DCLK1 594 TRPM5 488-20x3-5_C003T001.tif]

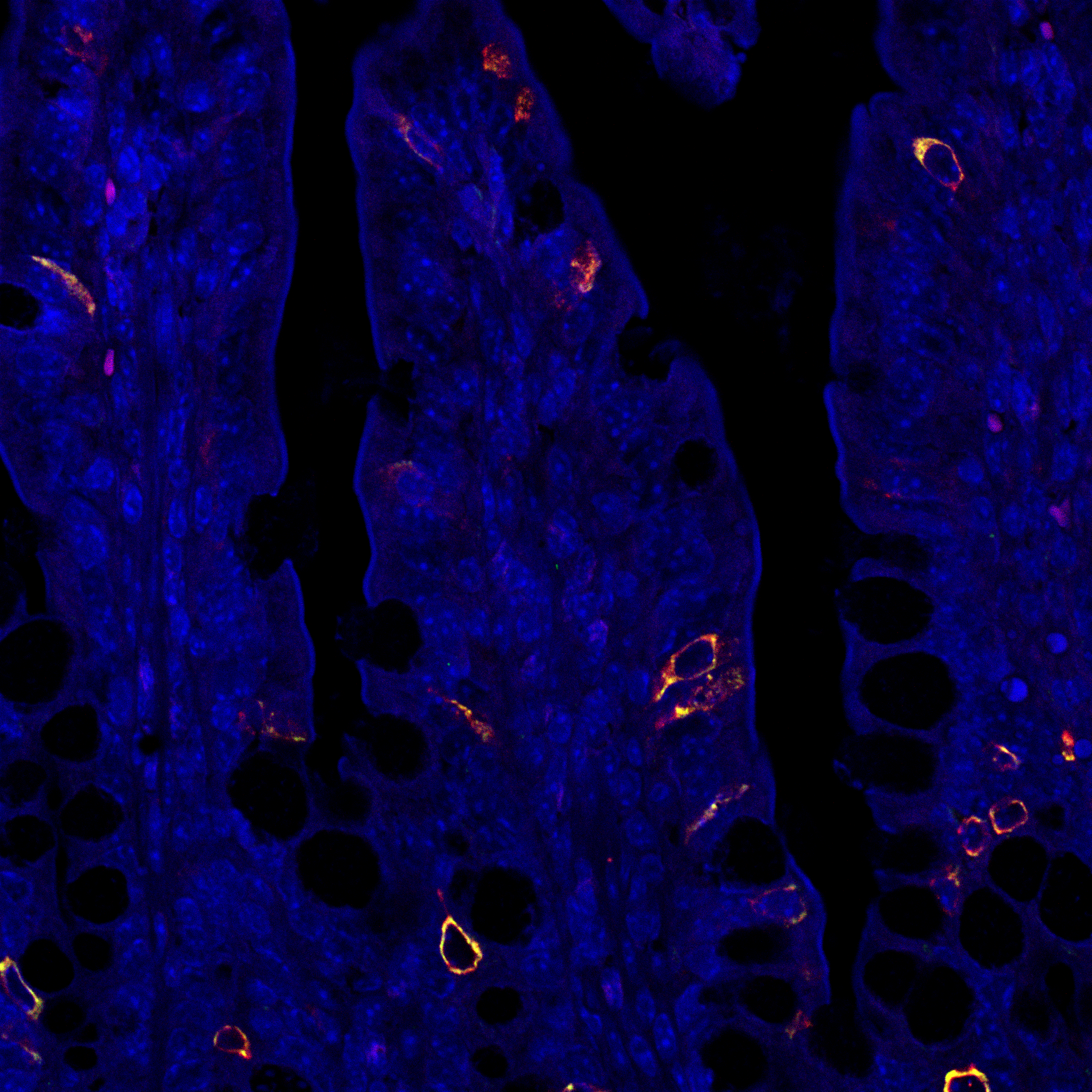

Supplement: Supplementary file 3 — Source data Fig. 1 [file 44321_2024_128_MOESM3_ESM.zip › EMM-2023-19008-V2-figure 1/EMM-2023-19008-V2-figure 1/figure 1F IF/C57 IL-25 8 DCLK1 594 TRPM5 488-20x3-5.tif.frames ╥╤╙├/C57 IL-25 8 DCLK1 594 TRPM5 488-20x3-5_T001.tif]

## Slide 1
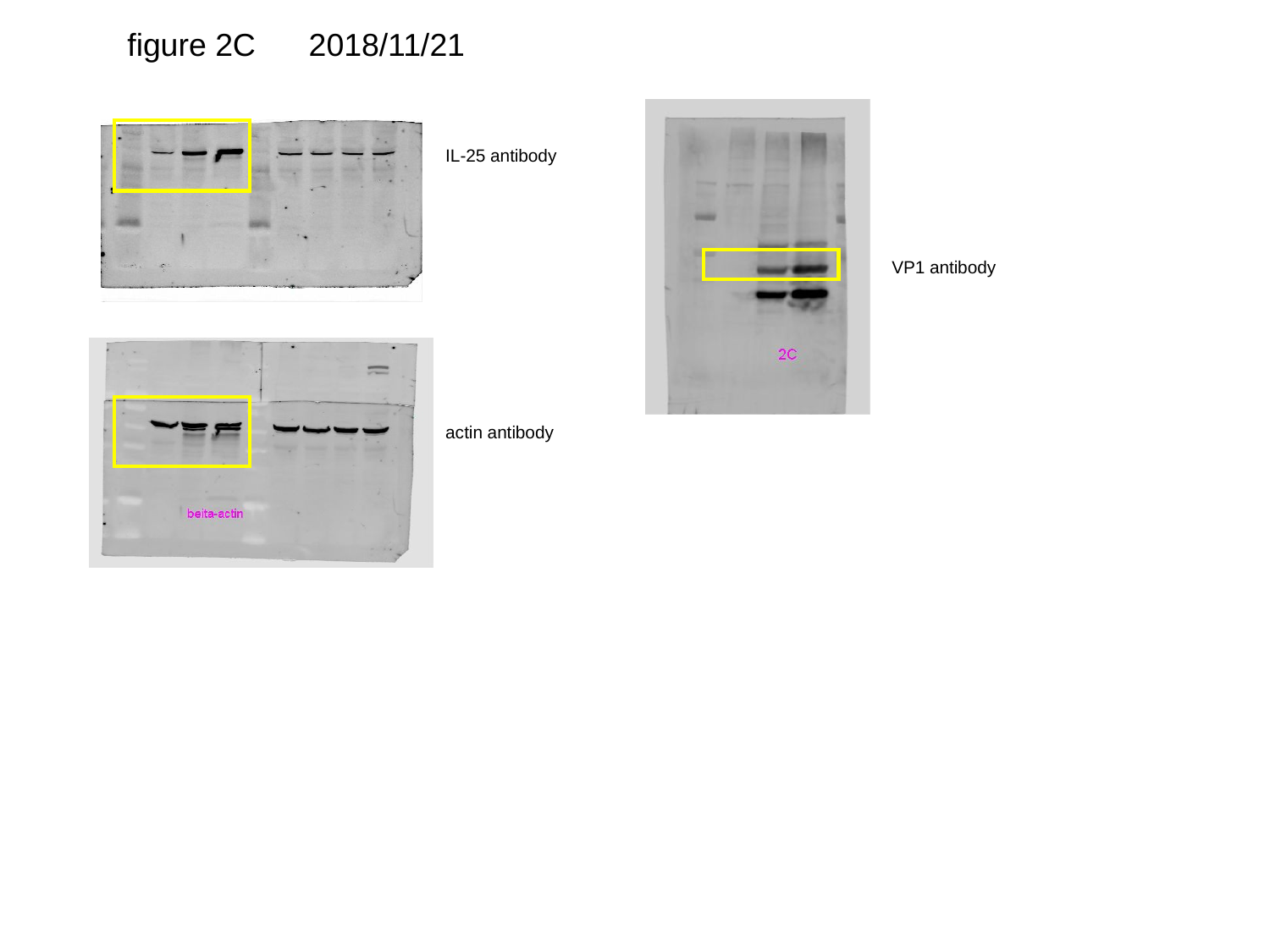

figure 2C 2018/11/21
IL-25 antibody
VP1 antibody
actin antibody

Supplement: Supplementary file 4 — Source data Fig. 2 [file 44321_2024_128_MOESM4_ESM.zip › EMM-2023-19008-V2-figure 2/figure 2C WB raw data/figure 2C.pptx]

## Slide 1
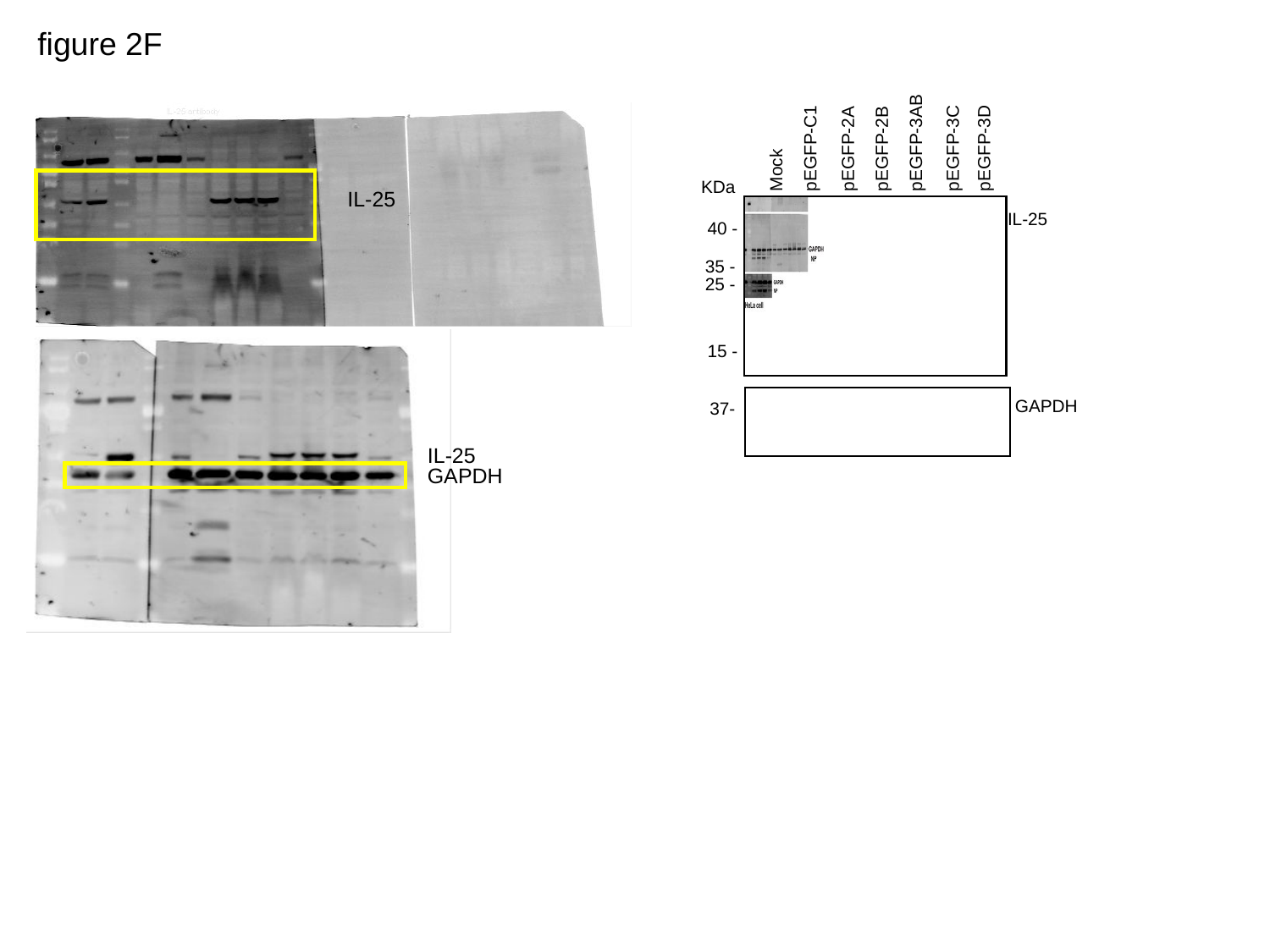

figure 2F
pEGFP-C1
pEGFP-2A
pEGFP-2B
pEGFP-3AB
pEGFP-3C
pEGFP-3D
Mock
KDa
IL-25
IL-25
40 -
35 -
25 -
15 -
GAPDH
37-
IL-25
GAPDH

Supplement: Supplementary file 4 — Source data Fig. 2 [file 44321_2024_128_MOESM4_ESM.zip › EMM-2023-19008-V2-figure 2/figure 2F WB raw data/figure 2F.pptx]

## Slide 1
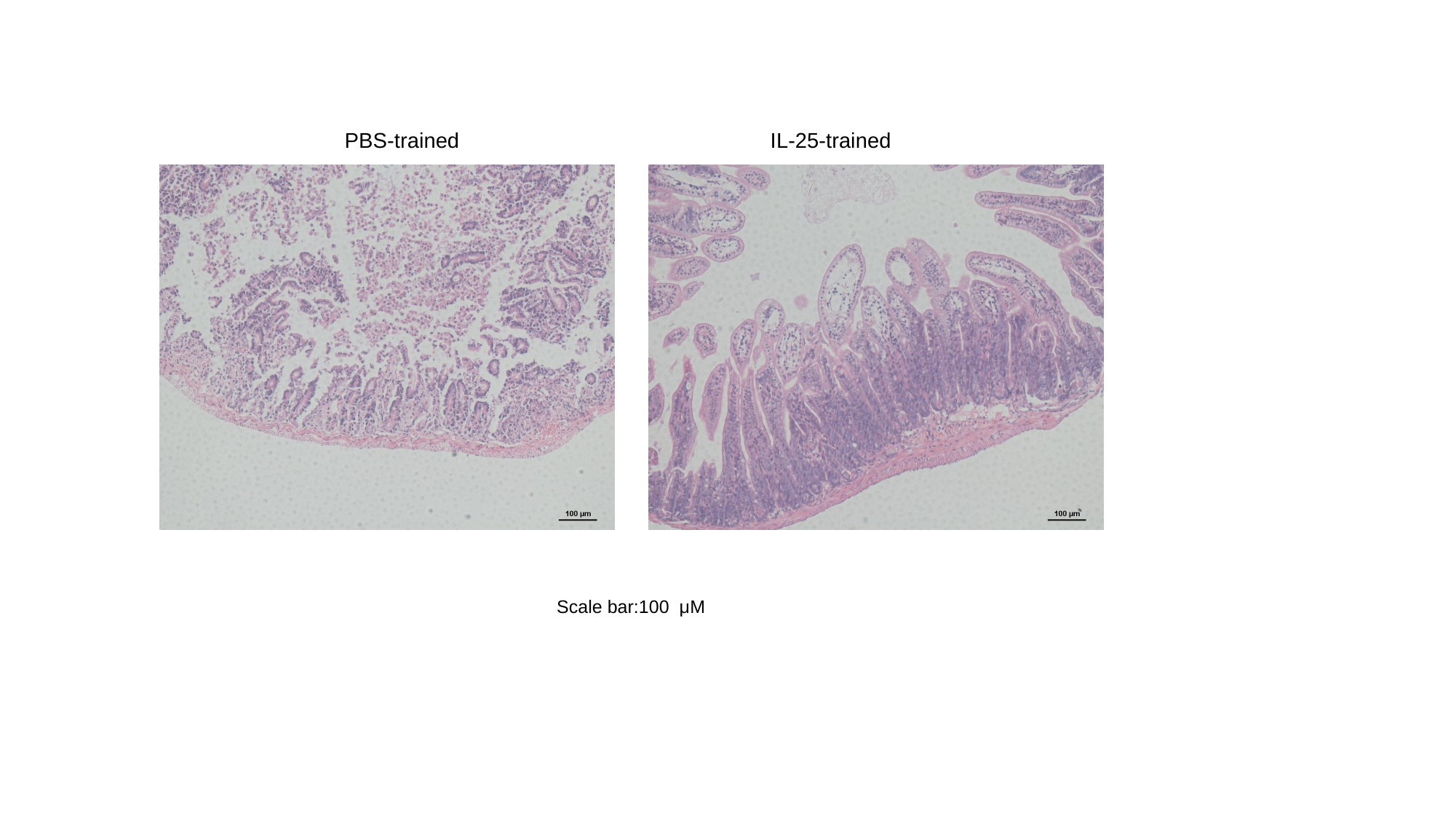

PBS-trained
IL-25-trained
Scale bar:100 μM

Supplement: Supplementary file 5 — Source data Fig. 3 [file 44321_2024_128_MOESM5_ESM.zip › EMM-2023-19008-V2-figure 3/figure 3/figure 3P/figure 3P.pptx]

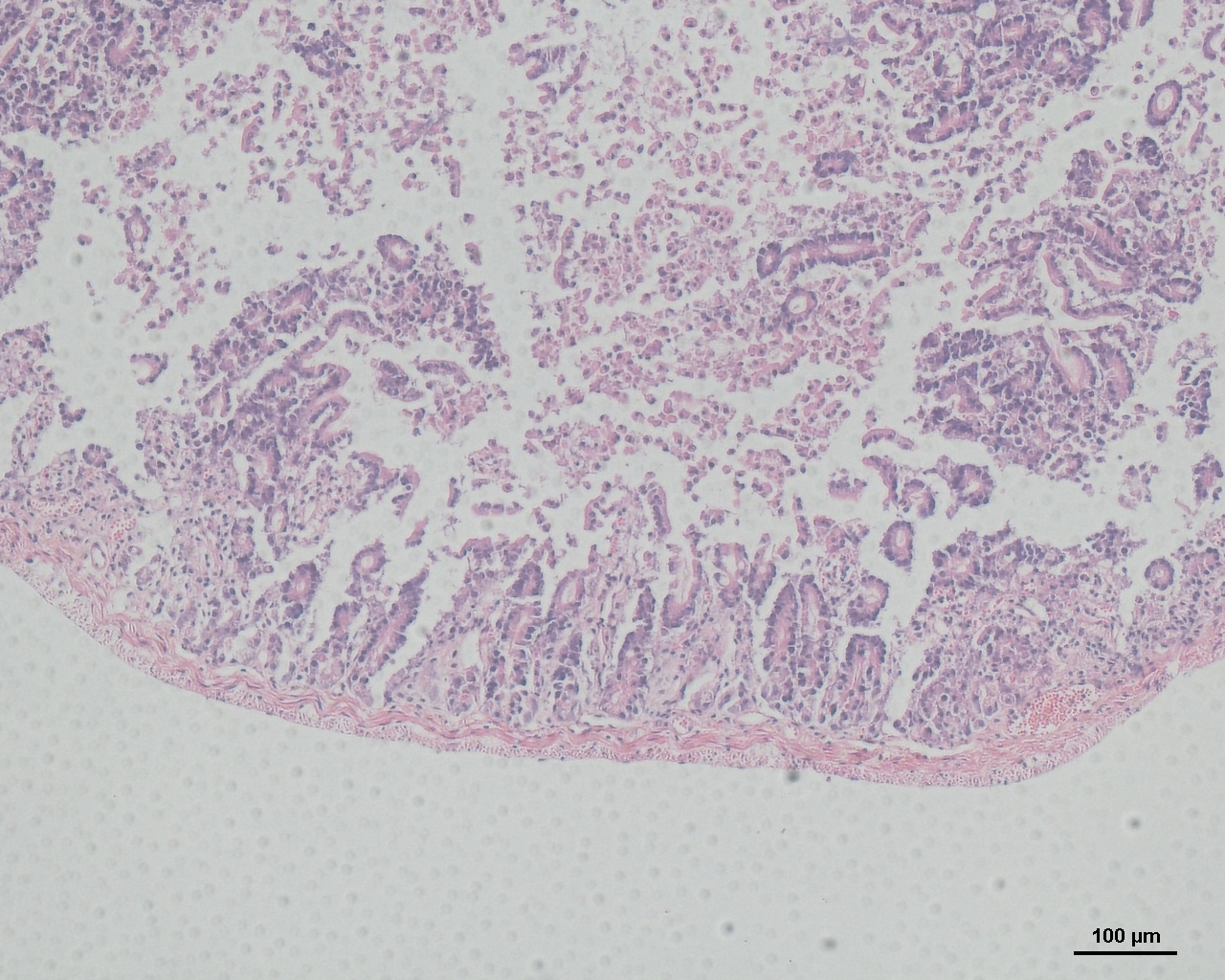

Supplement: Supplementary file 5 — Source data Fig. 3 [file 44321_2024_128_MOESM5_ESM.zip › EMM-2023-19008-V2-figure 3/figure 3/figure 3P/┼─╔π10x-EV71 C-1 100um ╥╤╙├.tif]

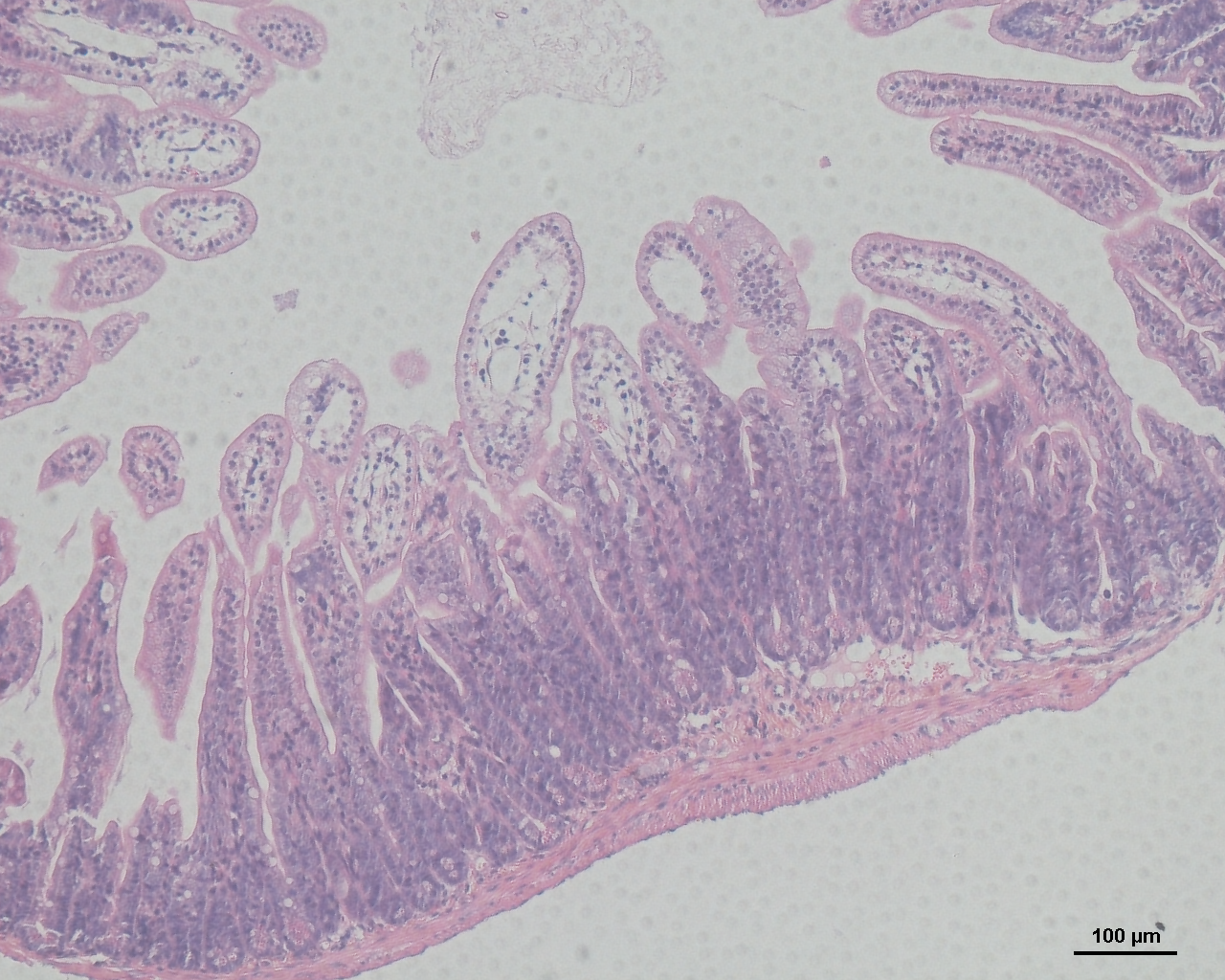

Supplement: Supplementary file 5 — Source data Fig. 3 [file 44321_2024_128_MOESM5_ESM.zip › EMM-2023-19008-V2-figure 3/figure 3/figure 3P/┼─╔π10x-il-25 D-2 100um ╥╤╙├.tif]

## Slide 1
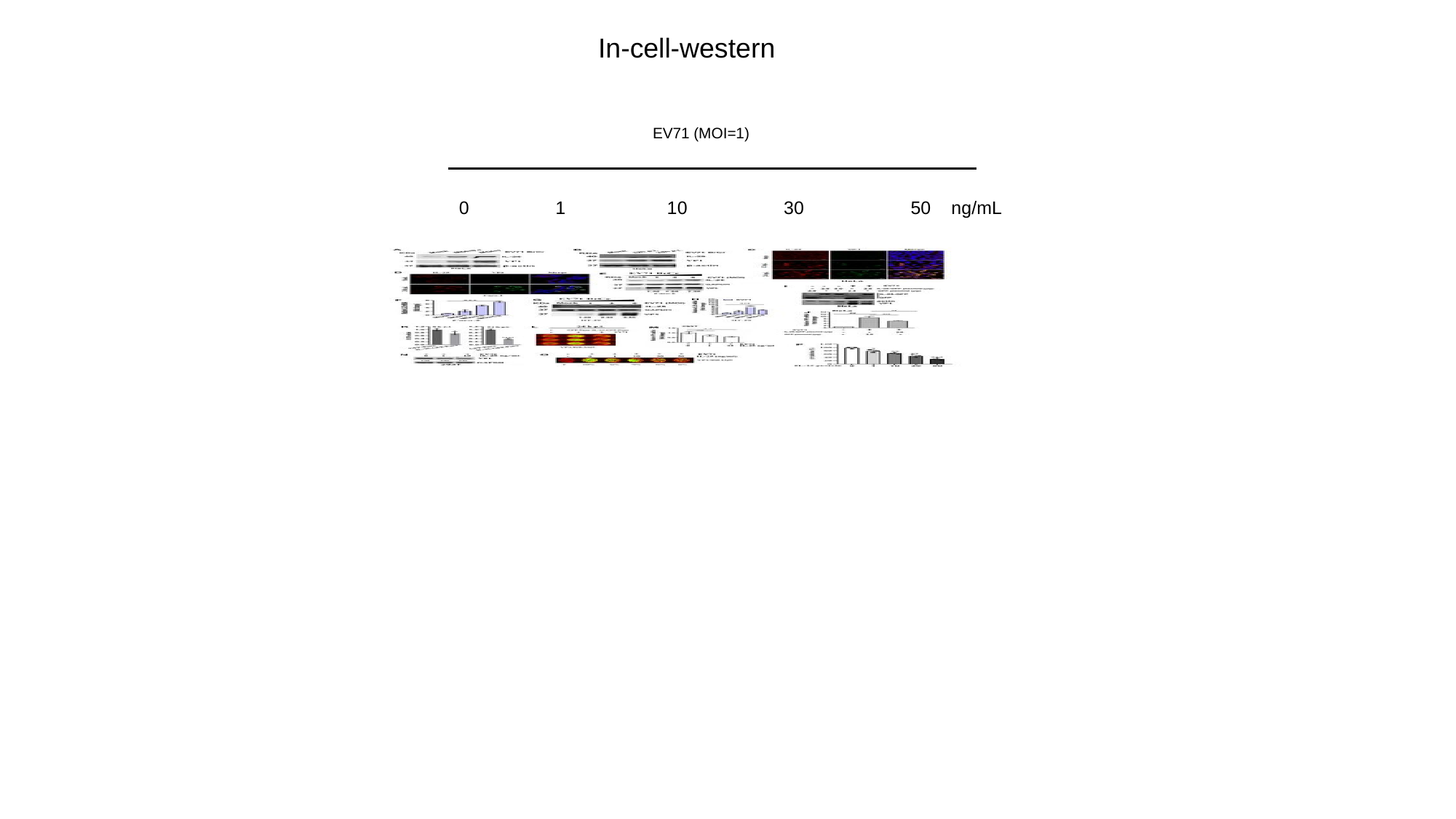

In-cell-western
EV71 (MOI=1)
 0 1 10 30 50 ng/mL

Supplement: Supplementary file 6 — Source data Fig. 4 [file 44321_2024_128_MOESM6_ESM.zip › EMM-2023-19008-V2-figure 4/figure 4F In-cell westernblot raw data/Figure 4F.pptx]

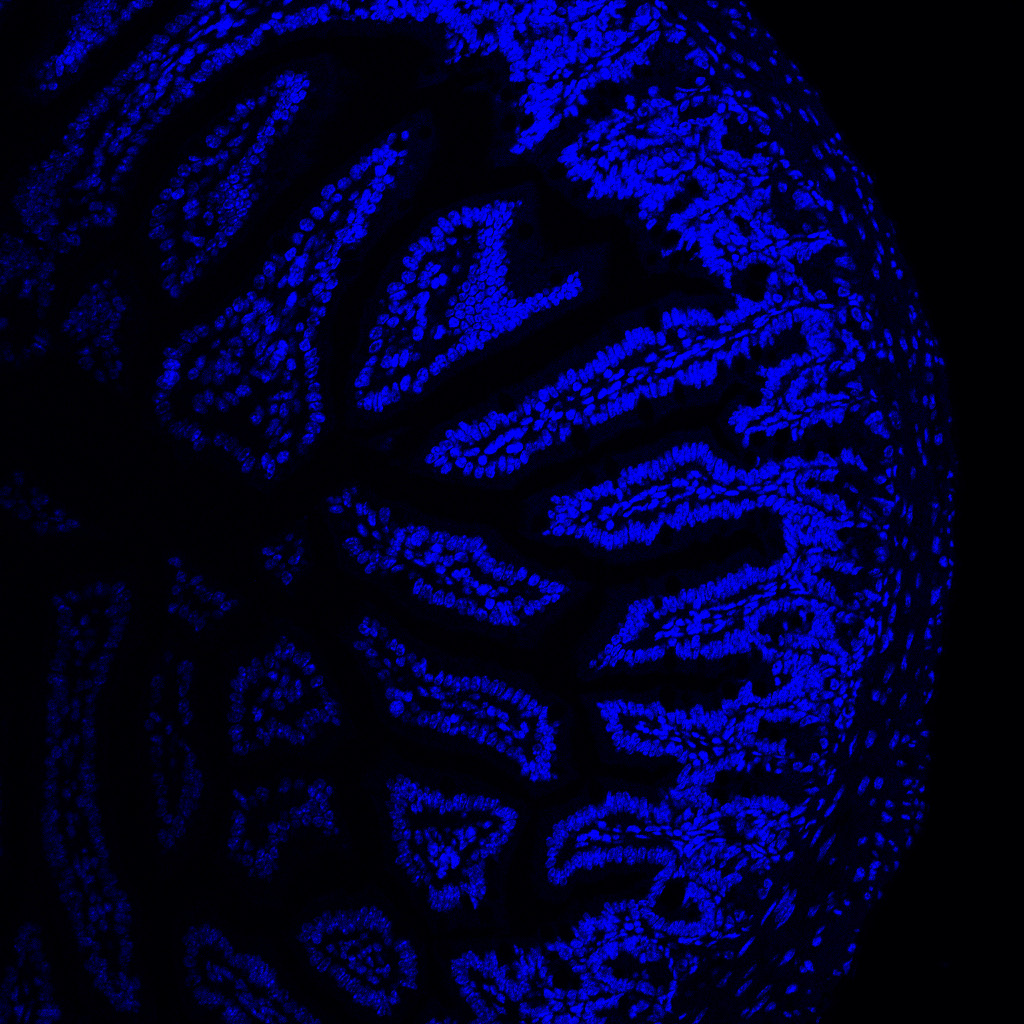

Supplement: Supplementary file 7 — Source data Fig. 5 [file 44321_2024_128_MOESM7_ESM.zip › EMM-2023-19008-V2-figure 5/figure 5D left/C57 -NO3-20x-1╥╤╙├.tif.frames/C57 -NO3-20x-1_C001T001.tif]

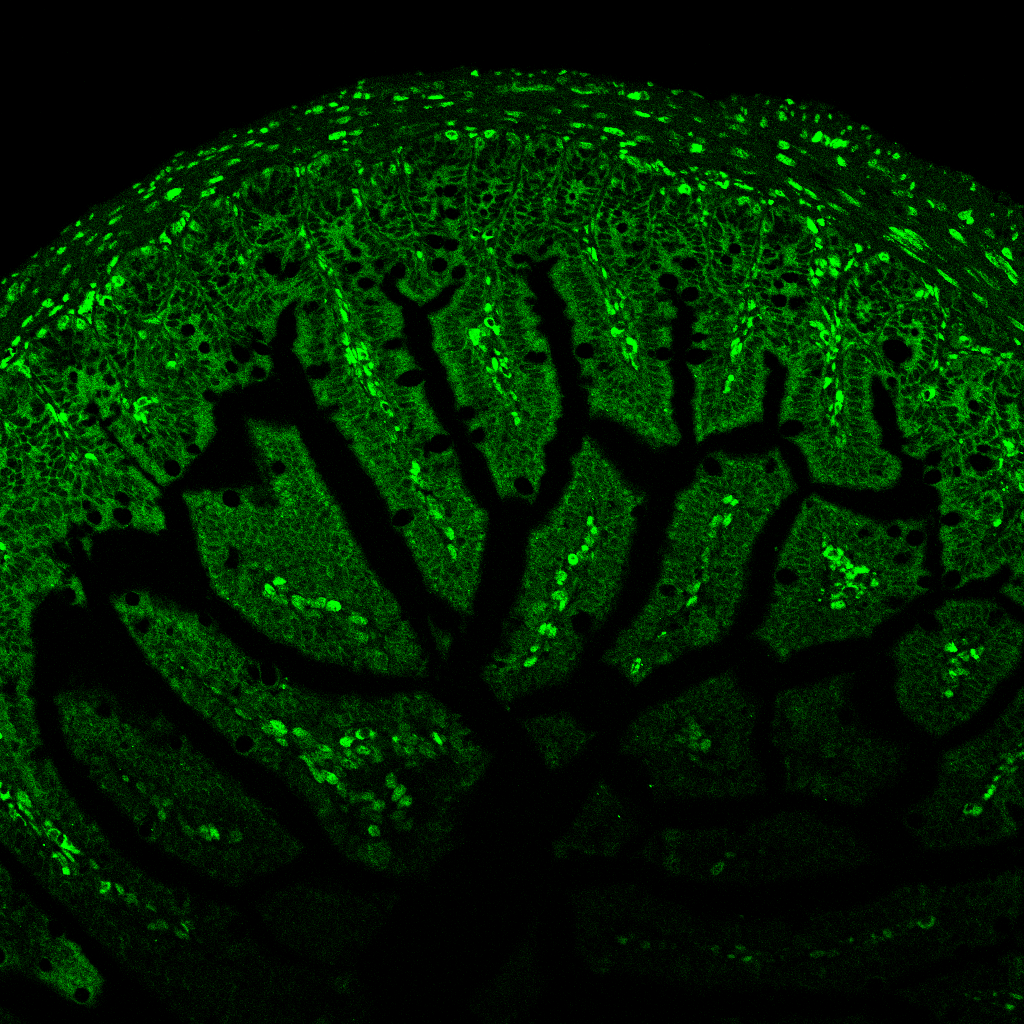

Supplement: Supplementary file 7 — Source data Fig. 5 [file 44321_2024_128_MOESM7_ESM.zip › EMM-2023-19008-V2-figure 5/figure 5D left/C57 -NO3-20x-1╥╤╙├.tif.frames/C57 -NO3-20x-1_C002T001.tif]

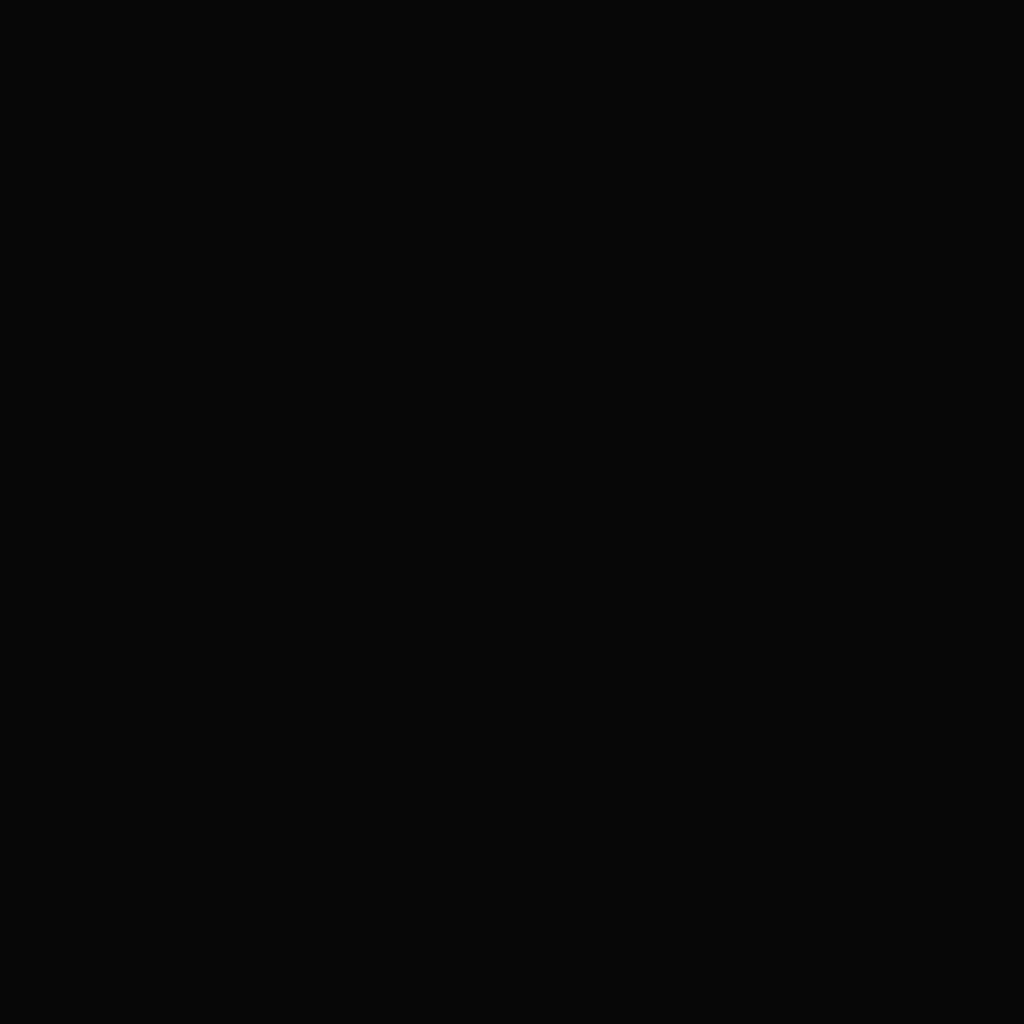

Supplement: Supplementary file 7 — Source data Fig. 5 [file 44321_2024_128_MOESM7_ESM.zip › EMM-2023-19008-V2-figure 5/figure 5D left/C57 -NO3-20x-1╥╤╙├.tif.frames/C57 -NO3-20x-1_C003T001.tif]

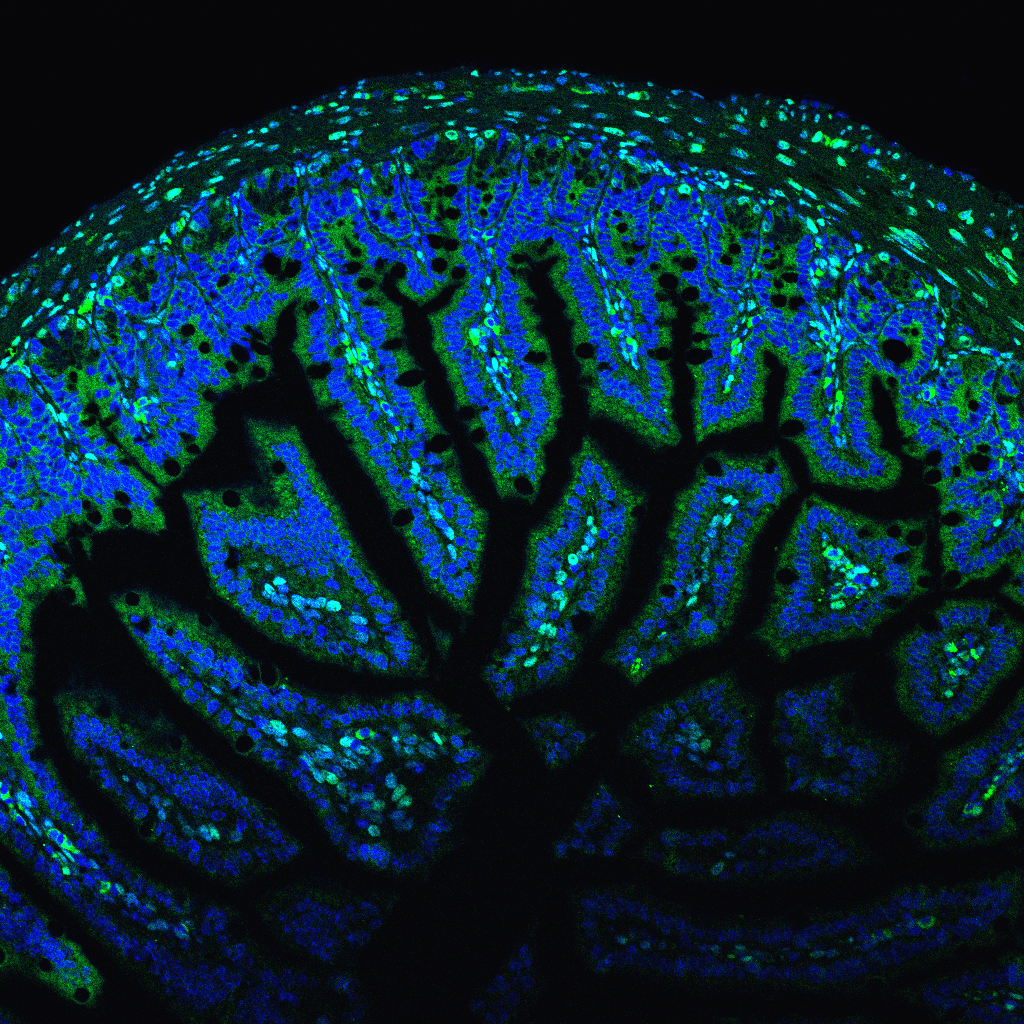

Supplement: Supplementary file 7 — Source data Fig. 5 [file 44321_2024_128_MOESM7_ESM.zip › EMM-2023-19008-V2-figure 5/figure 5D left/C57 -NO3-20x-1╥╤╙├.tif.frames/C57 -NO3-20x-1_T001.tif]

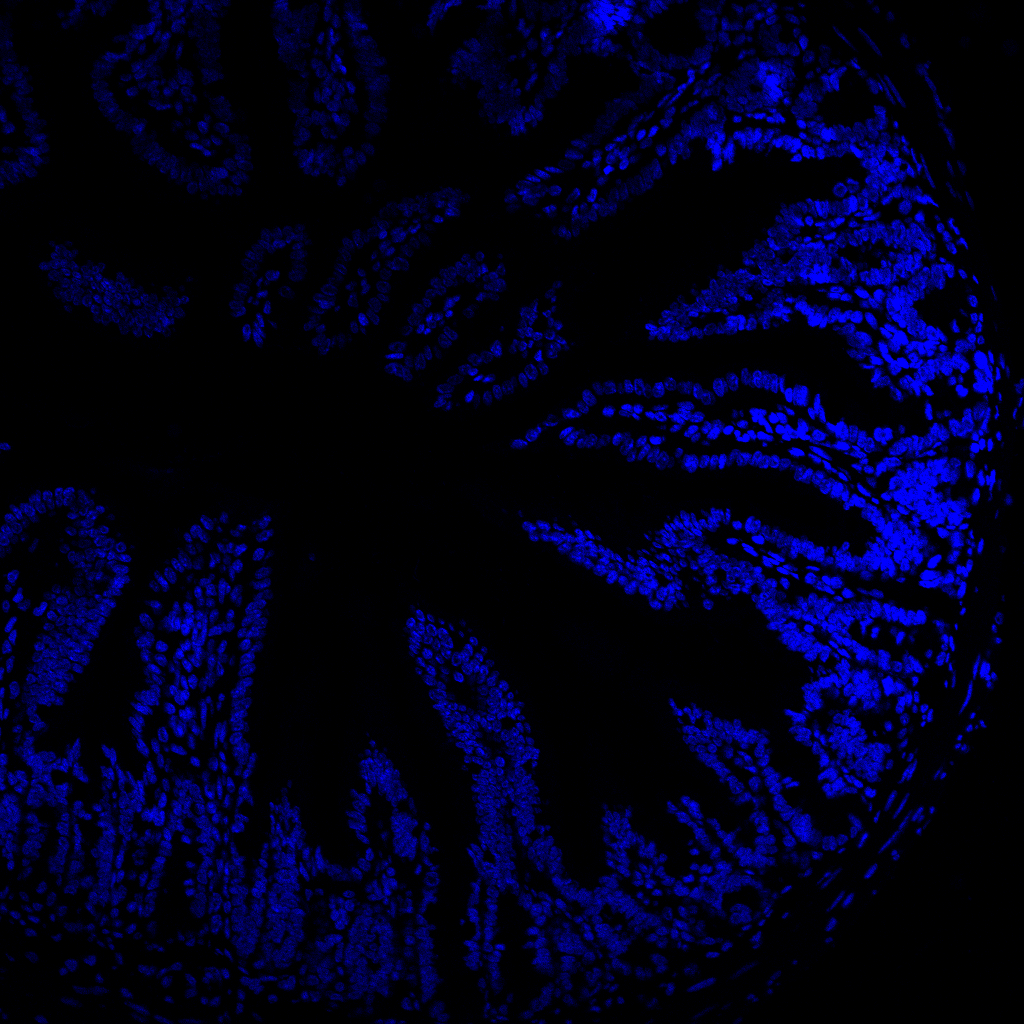

Supplement: Supplementary file 7 — Source data Fig. 5 [file 44321_2024_128_MOESM7_ESM.zip › EMM-2023-19008-V2-figure 5/figure 5D left/C57-Mock-VP1488-20x-1-1╥╤╙├.tif.frames/C57-Mock-VP1488-20x-1-1_C001T001.tif]

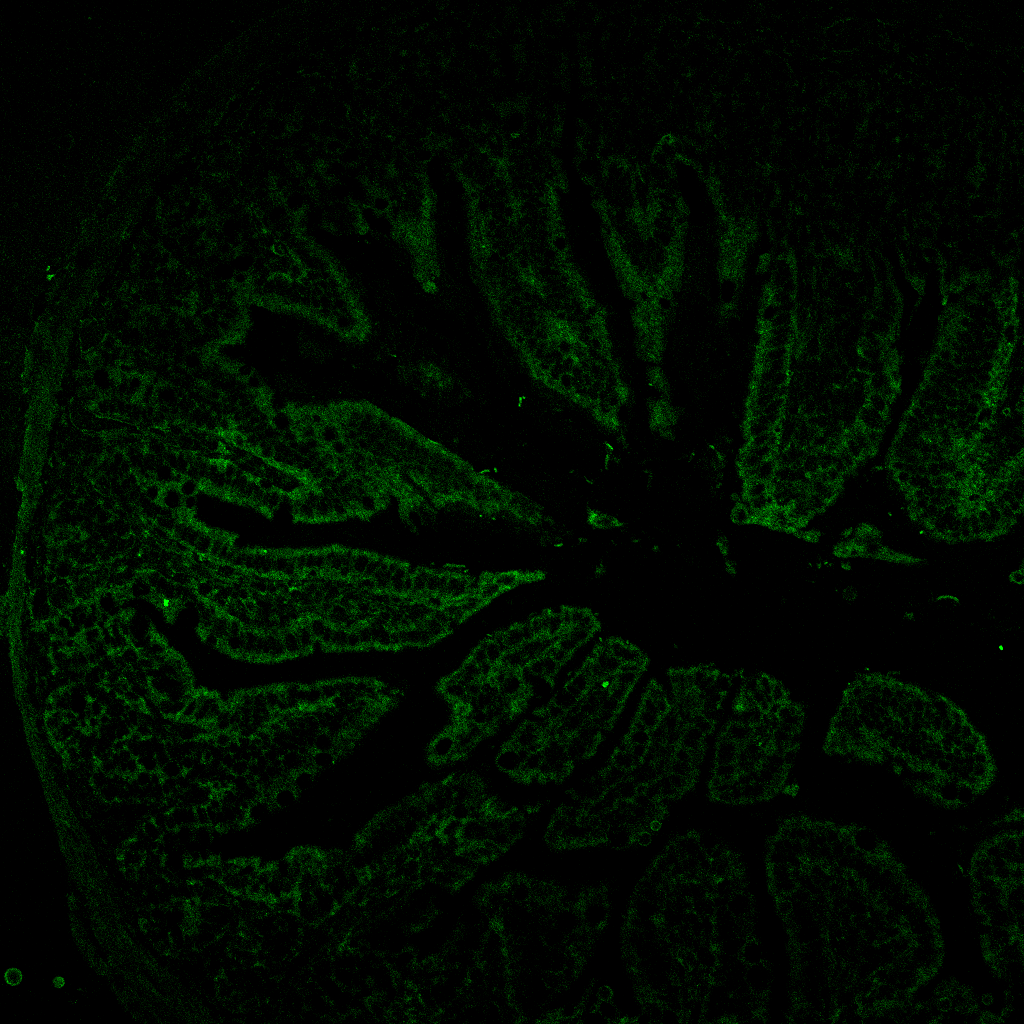

Supplement: Supplementary file 7 — Source data Fig. 5 [file 44321_2024_128_MOESM7_ESM.zip › EMM-2023-19008-V2-figure 5/figure 5D left/C57-Mock-VP1488-20x-1-1╥╤╙├.tif.frames/C57-Mock-VP1488-20x-1-1_C002T001.tif]

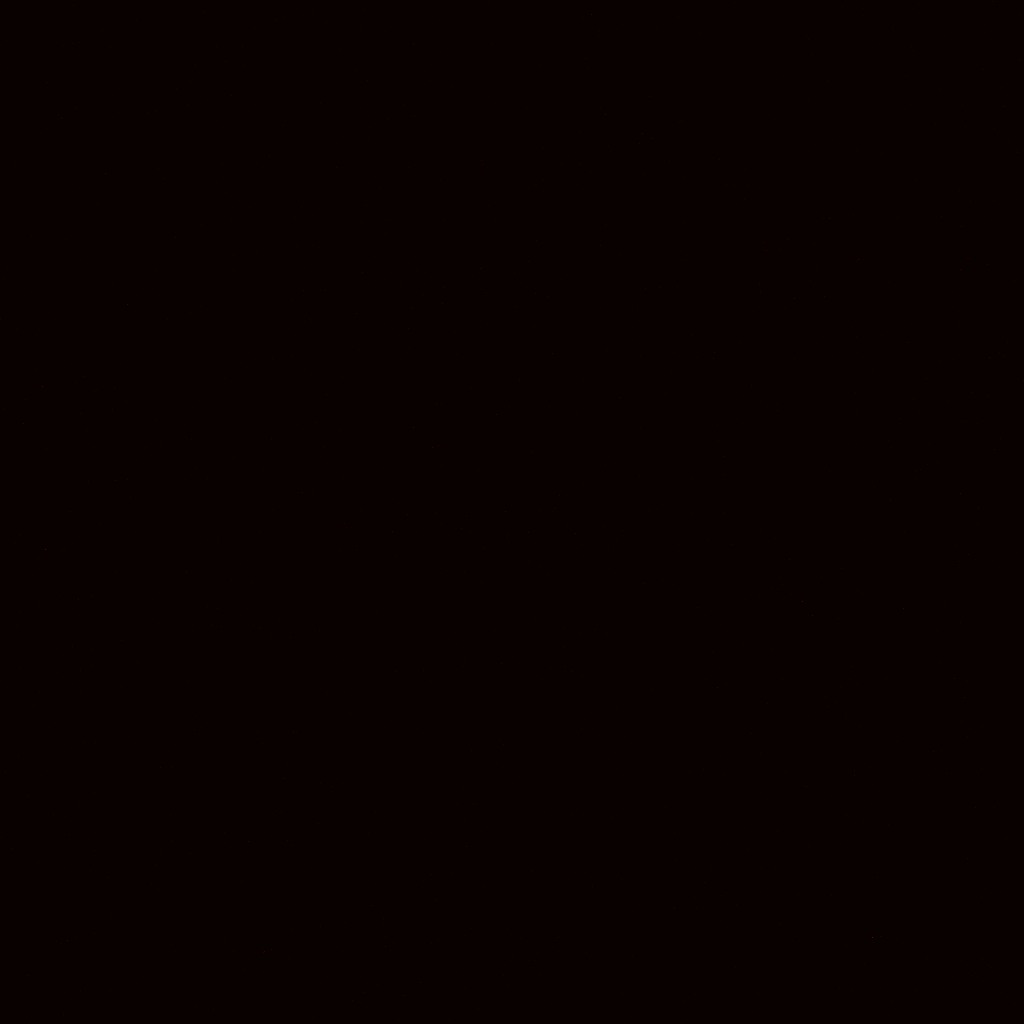

Supplement: Supplementary file 7 — Source data Fig. 5 [file 44321_2024_128_MOESM7_ESM.zip › EMM-2023-19008-V2-figure 5/figure 5D left/C57-Mock-VP1488-20x-1-1╥╤╙├.tif.frames/C57-Mock-VP1488-20x-1-1_C003T001.tif]

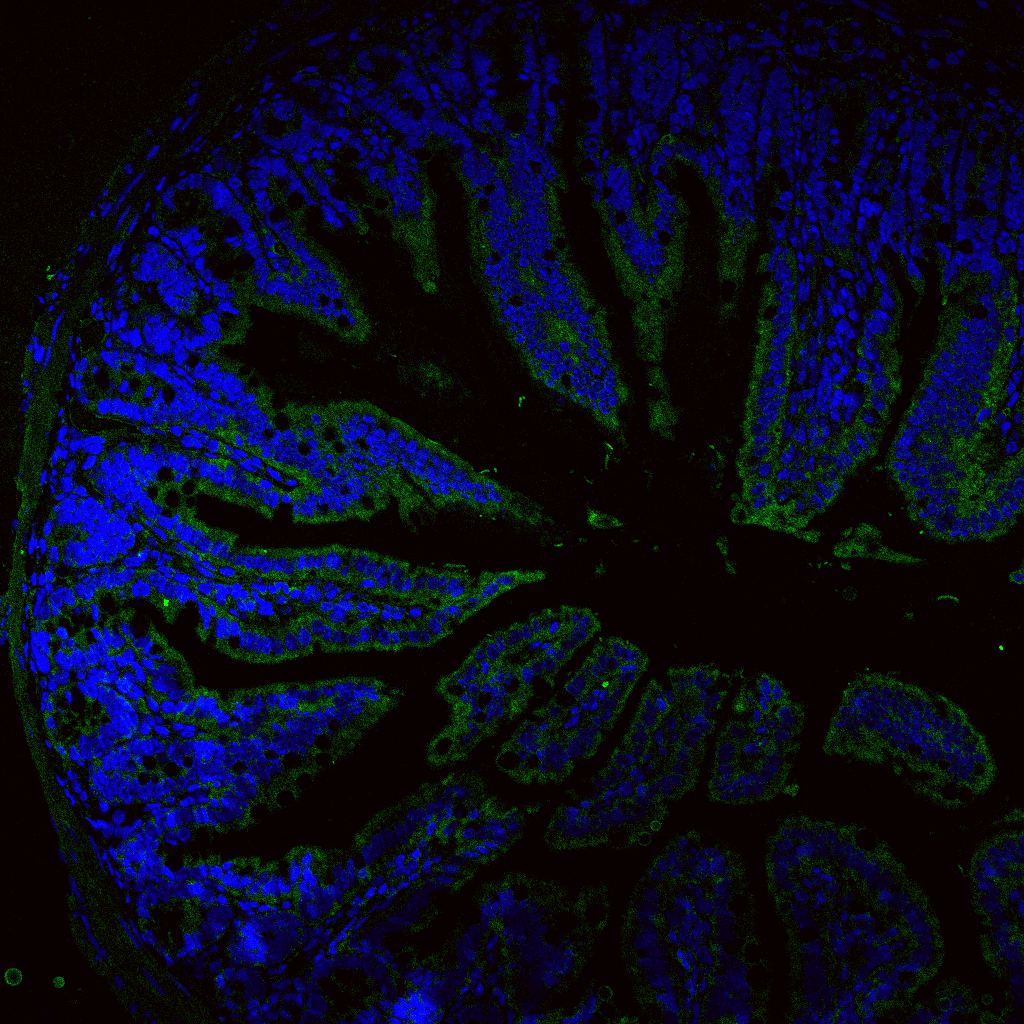

Supplement: Supplementary file 7 — Source data Fig. 5 [file 44321_2024_128_MOESM7_ESM.zip › EMM-2023-19008-V2-figure 5/figure 5D left/C57-Mock-VP1488-20x-1-1╥╤╙├.tif.frames/C57-Mock-VP1488-20x-1-1_T001.tif]

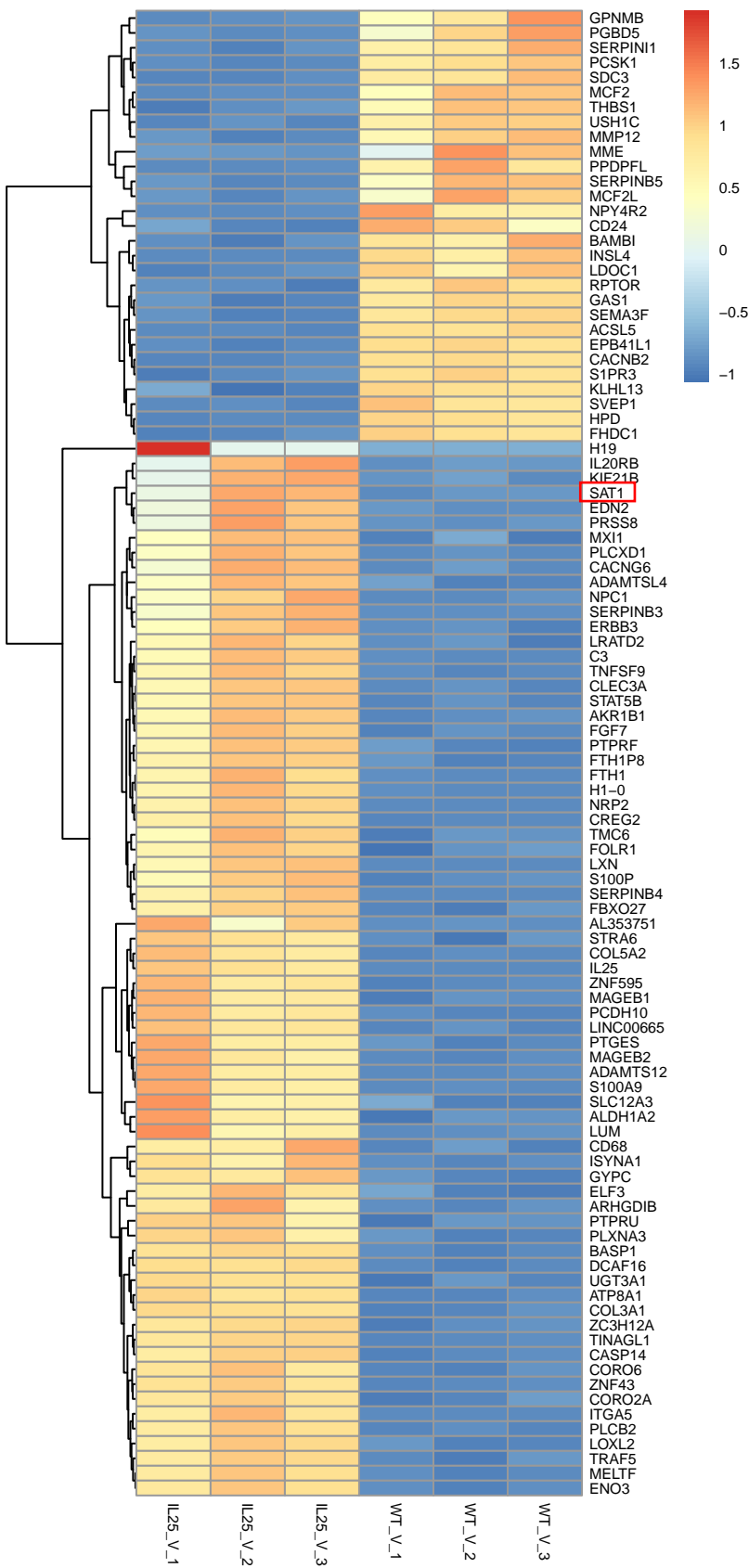

Supplement: Supplementary file 8 — Source data Fig. 6 [file 44321_2024_128_MOESM8_ESM.zip › EMM-2023-19008-V2-figure 6/figure 6A/IL25_VVSWT_V_genes_heatmap.pdf]

## Slide 1
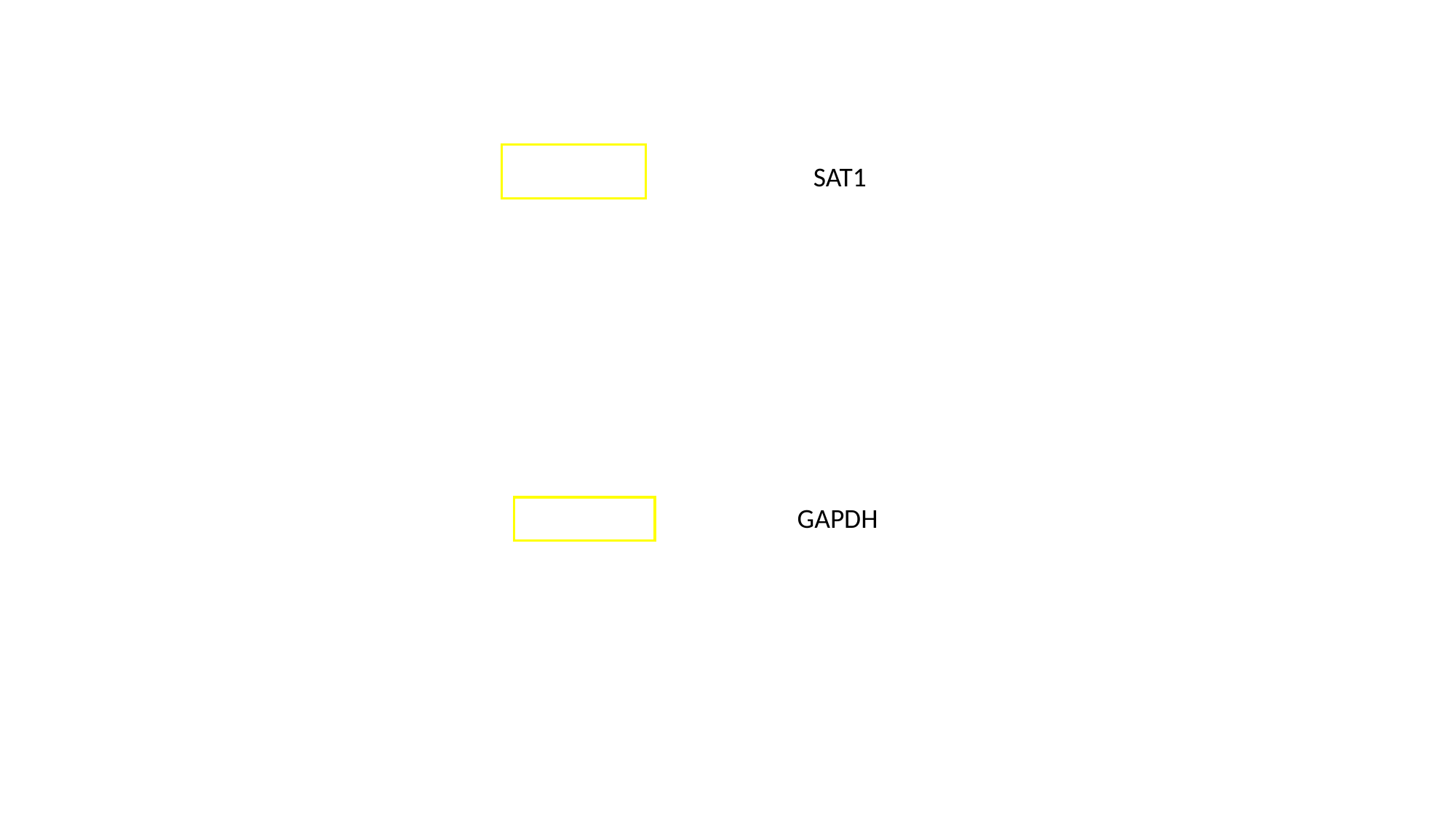

SAT1
GAPDH

Supplement: Supplementary file 8 — Source data Fig. 6 [file 44321_2024_128_MOESM8_ESM.zip › EMM-2023-19008-V2-figure 6/figure 6M Left WB raw data/figure 6M.pptx]

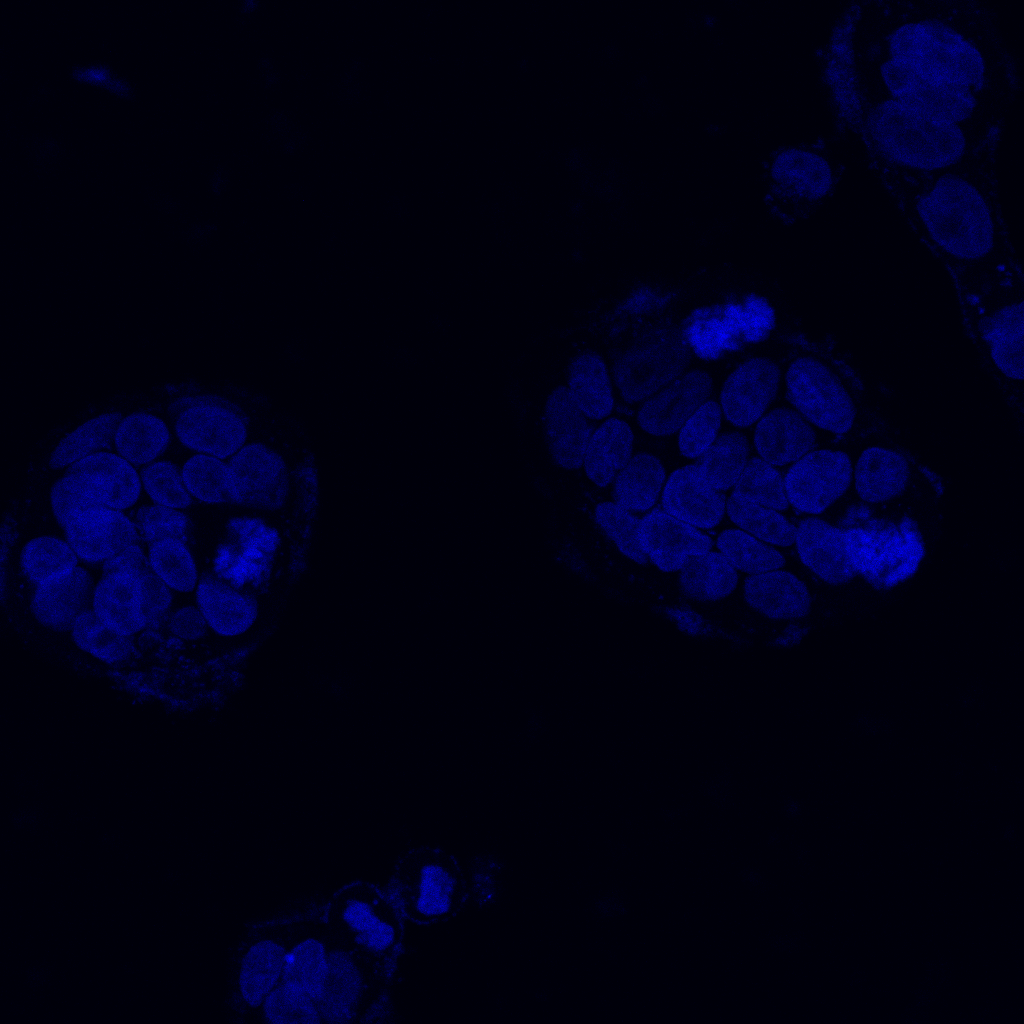

Supplement: Supplementary file 10 — Source data Fig. 8 [file 44321_2024_128_MOESM10_ESM.zip › EMM-2023-19008-V2-figure 8/figure 8A/ú¿1ú⌐ shNC duo594-SAT1488-20x3-3.tif.frames/shNC duo594-SAT1488-20x3-3_C001T001.tif]

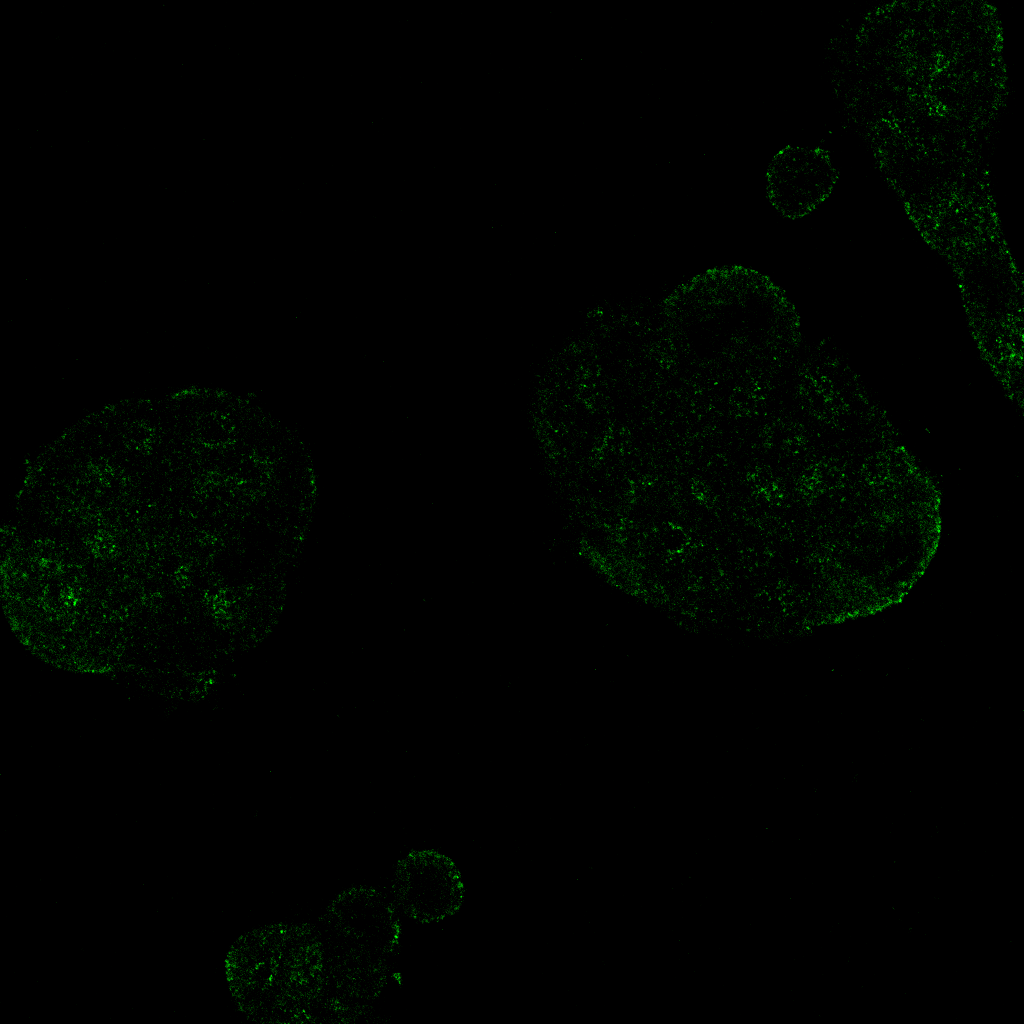

Supplement: Supplementary file 10 — Source data Fig. 8 [file 44321_2024_128_MOESM10_ESM.zip › EMM-2023-19008-V2-figure 8/figure 8A/ú¿1ú⌐ shNC duo594-SAT1488-20x3-3.tif.frames/shNC duo594-SAT1488-20x3-3_C002T001.tif]

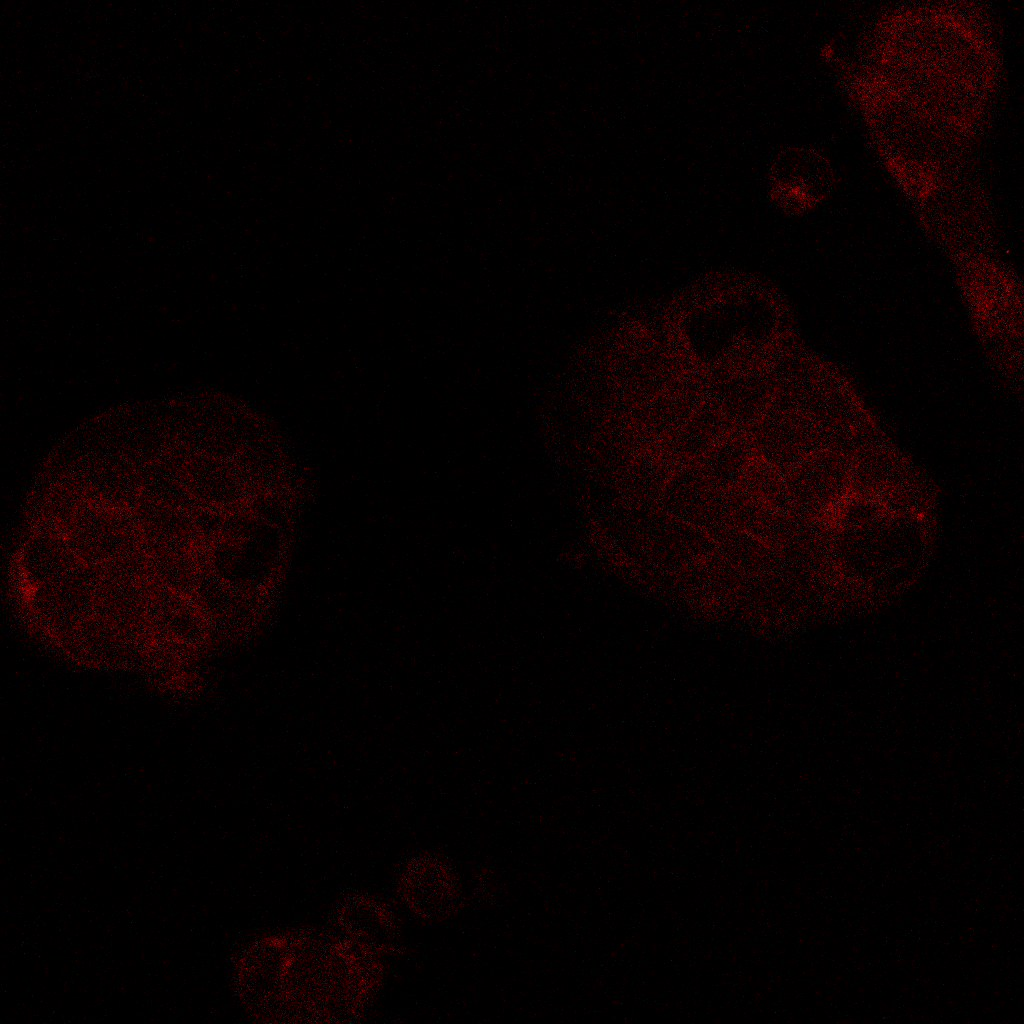

Supplement: Supplementary file 10 — Source data Fig. 8 [file 44321_2024_128_MOESM10_ESM.zip › EMM-2023-19008-V2-figure 8/figure 8A/ú¿1ú⌐ shNC duo594-SAT1488-20x3-3.tif.frames/shNC duo594-SAT1488-20x3-3_C003T001.tif]

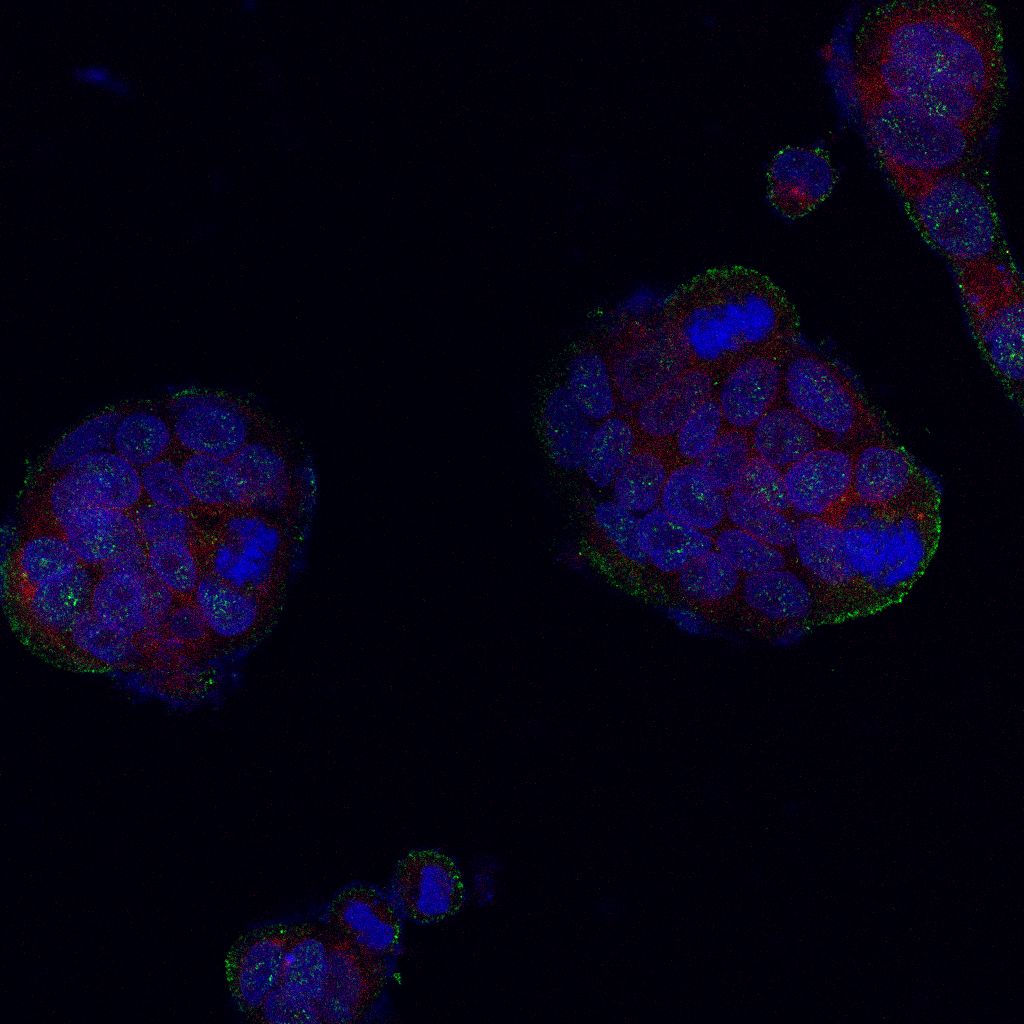

Supplement: Supplementary file 10 — Source data Fig. 8 [file 44321_2024_128_MOESM10_ESM.zip › EMM-2023-19008-V2-figure 8/figure 8A/ú¿1ú⌐ shNC duo594-SAT1488-20x3-3.tif.frames/shNC duo594-SAT1488-20x3-3_T001.tif]

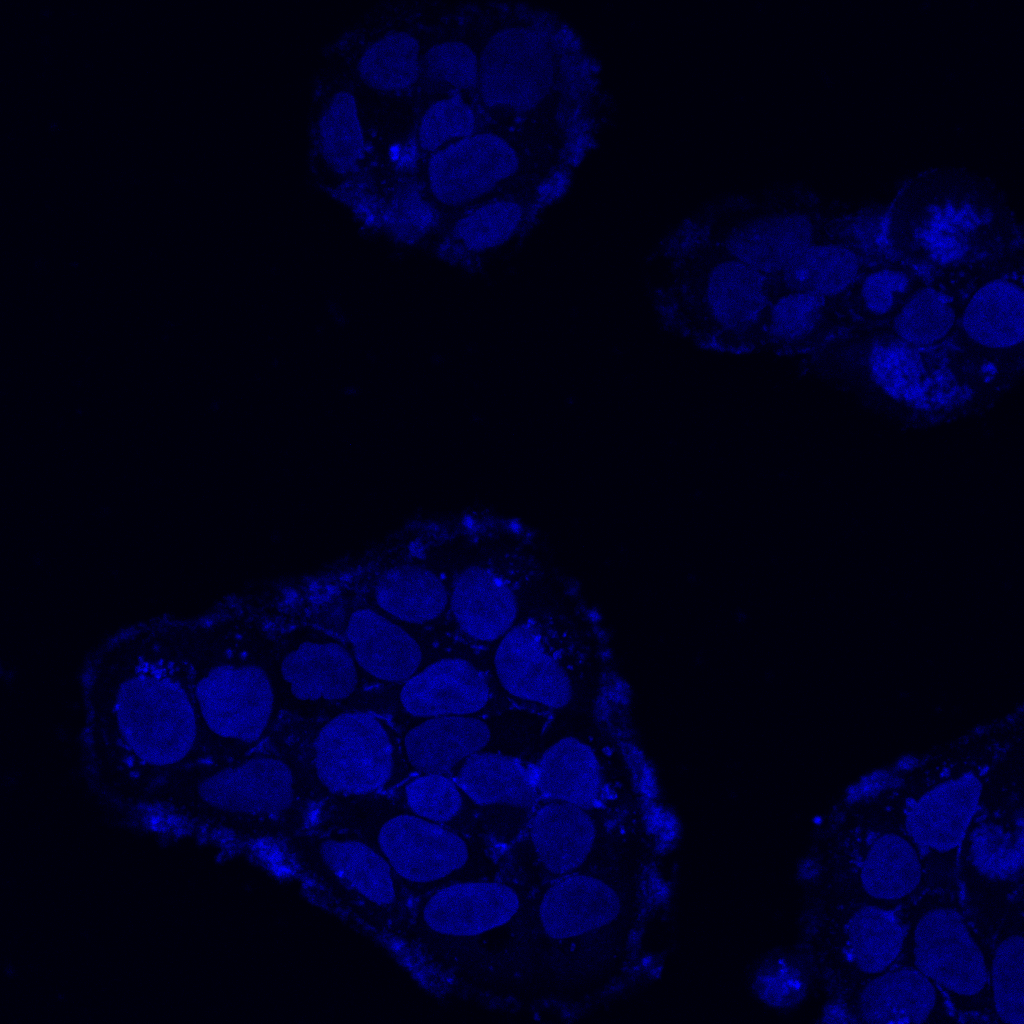

Supplement: Supplementary file 10 — Source data Fig. 8 [file 44321_2024_128_MOESM10_ESM.zip › EMM-2023-19008-V2-figure 8/figure 8A/ú¿2ú⌐ shNC+rhIL-25 duo594-SAT1488-20x3-3.tif (2).frames/shNC+IL-25duo594-SAT1488-20x3-1_C001T001.tif]

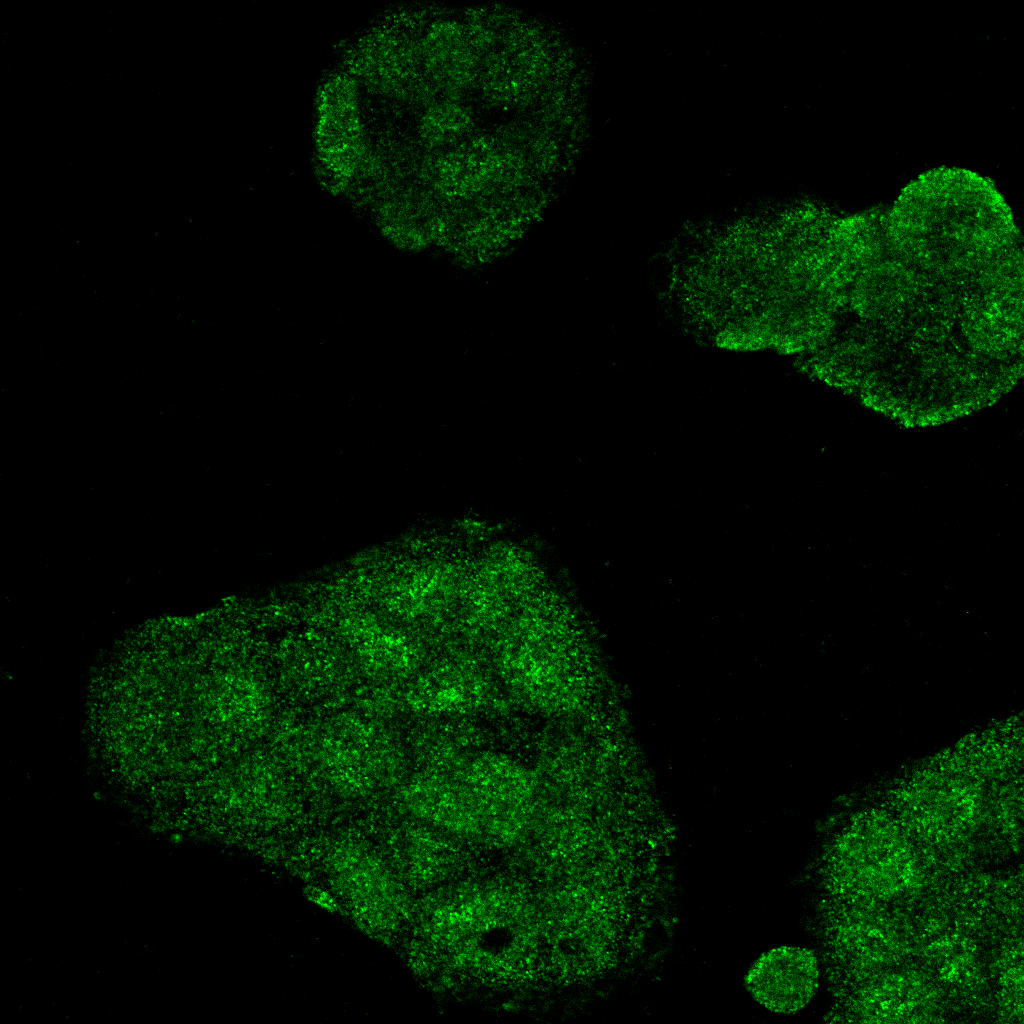

Supplement: Supplementary file 10 — Source data Fig. 8 [file 44321_2024_128_MOESM10_ESM.zip › EMM-2023-19008-V2-figure 8/figure 8A/ú¿2ú⌐ shNC+rhIL-25 duo594-SAT1488-20x3-3.tif (2).frames/shNC+IL-25duo594-SAT1488-20x3-1_C002T001.tif]

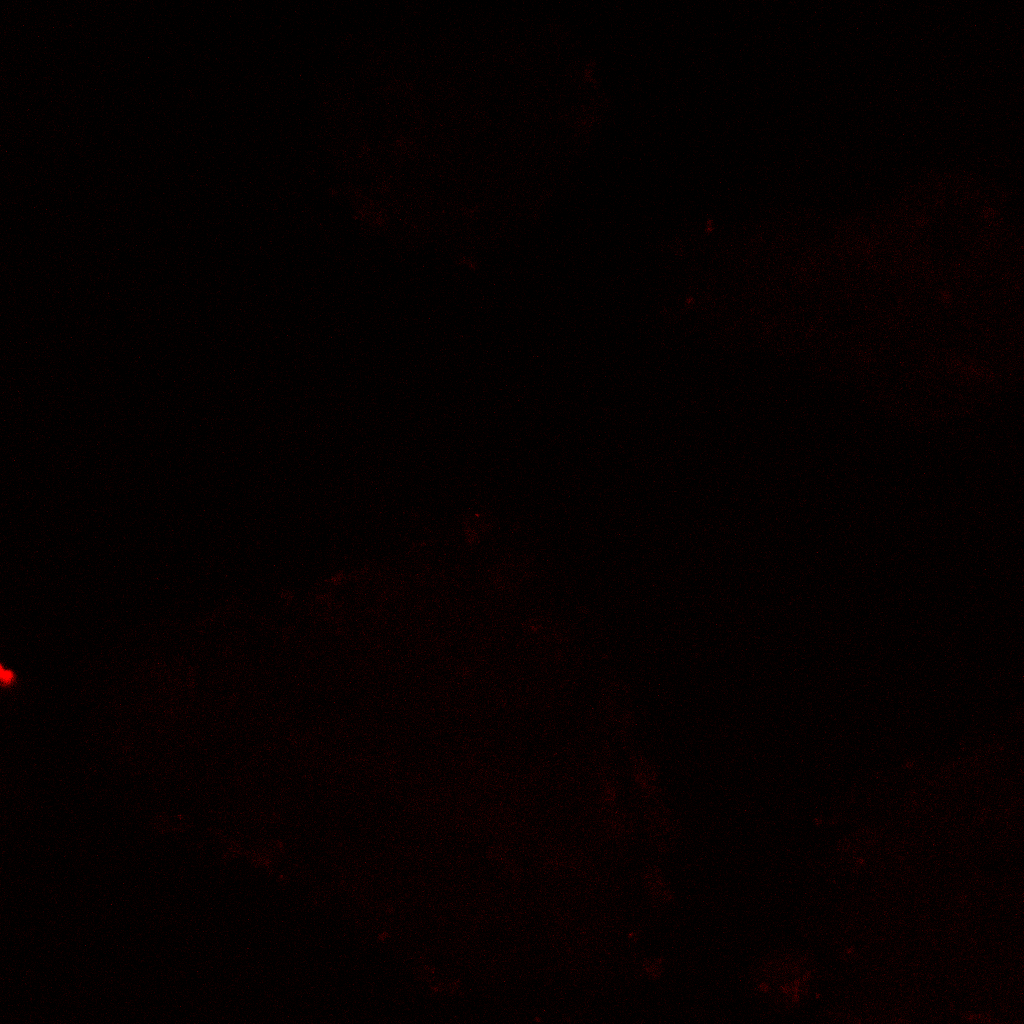

Supplement: Supplementary file 10 — Source data Fig. 8 [file 44321_2024_128_MOESM10_ESM.zip › EMM-2023-19008-V2-figure 8/figure 8A/ú¿2ú⌐ shNC+rhIL-25 duo594-SAT1488-20x3-3.tif (2).frames/shNC+IL-25duo594-SAT1488-20x3-1_C003T001.tif]

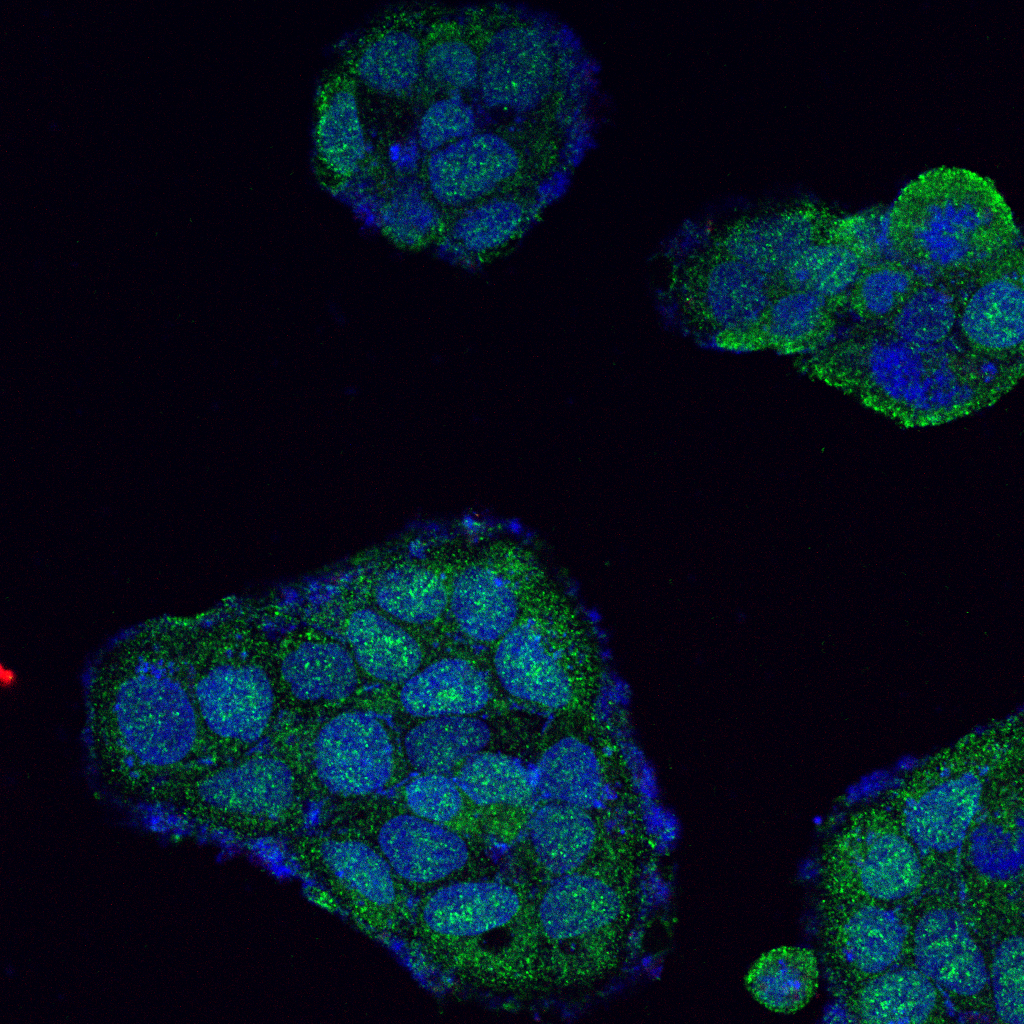

Supplement: Supplementary file 10 — Source data Fig. 8 [file 44321_2024_128_MOESM10_ESM.zip › EMM-2023-19008-V2-figure 8/figure 8A/ú¿2ú⌐ shNC+rhIL-25 duo594-SAT1488-20x3-3.tif (2).frames/shNC+IL-25duo594-SAT1488-20x3-1_T001.tif]

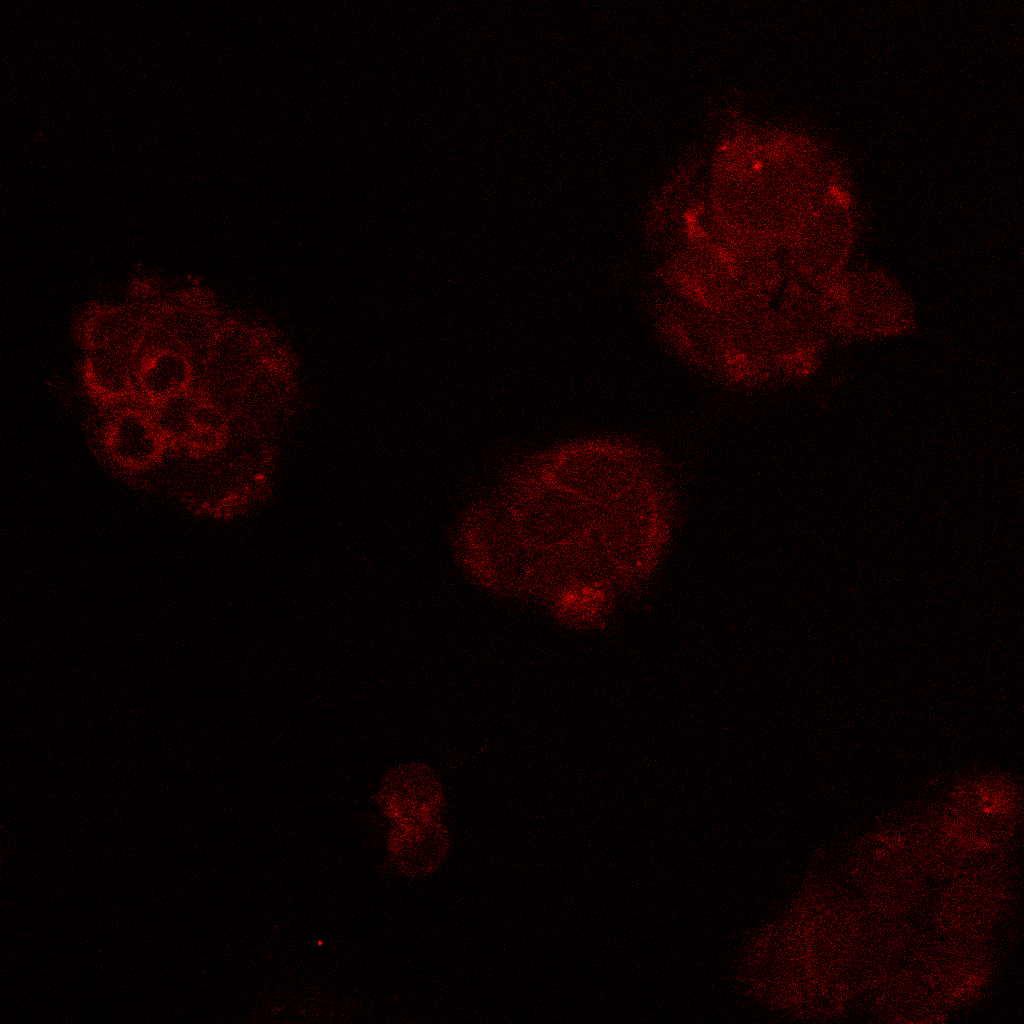

Supplement: Supplementary file 10 — Source data Fig. 8 [file 44321_2024_128_MOESM10_ESM.zip › EMM-2023-19008-V2-figure 8/figure 8A/ú¿3ú⌐ shSAT1#03 duo594-SAT1488-20x3-3.tif (3).frames/shSAT1#3 duo594-SAT1488-20x3-2_C003T001.tif]

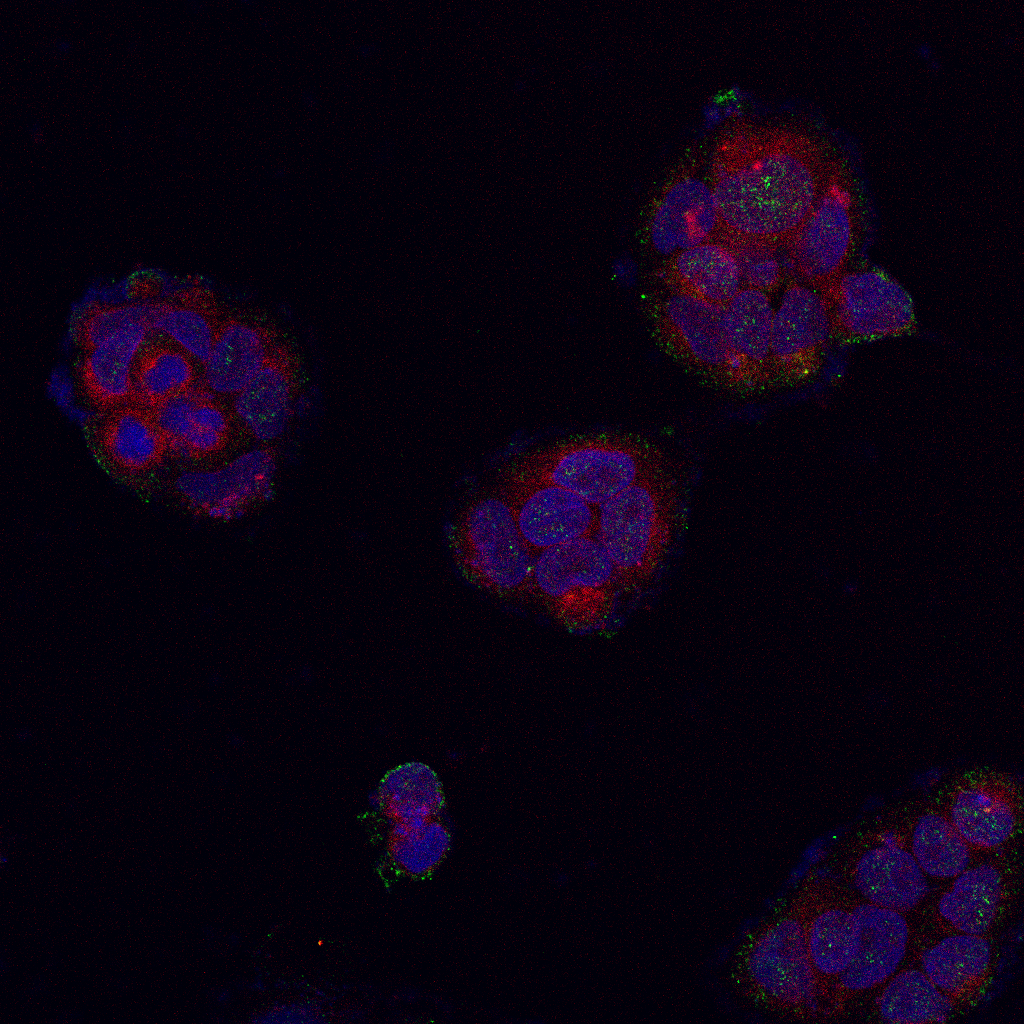

Supplement: Supplementary file 10 — Source data Fig. 8 [file 44321_2024_128_MOESM10_ESM.zip › EMM-2023-19008-V2-figure 8/figure 8A/ú¿3ú⌐ shSAT1#03 duo594-SAT1488-20x3-3.tif (3).frames/shSAT1#3 duo594-SAT1488-20x3-2_T001.tif]

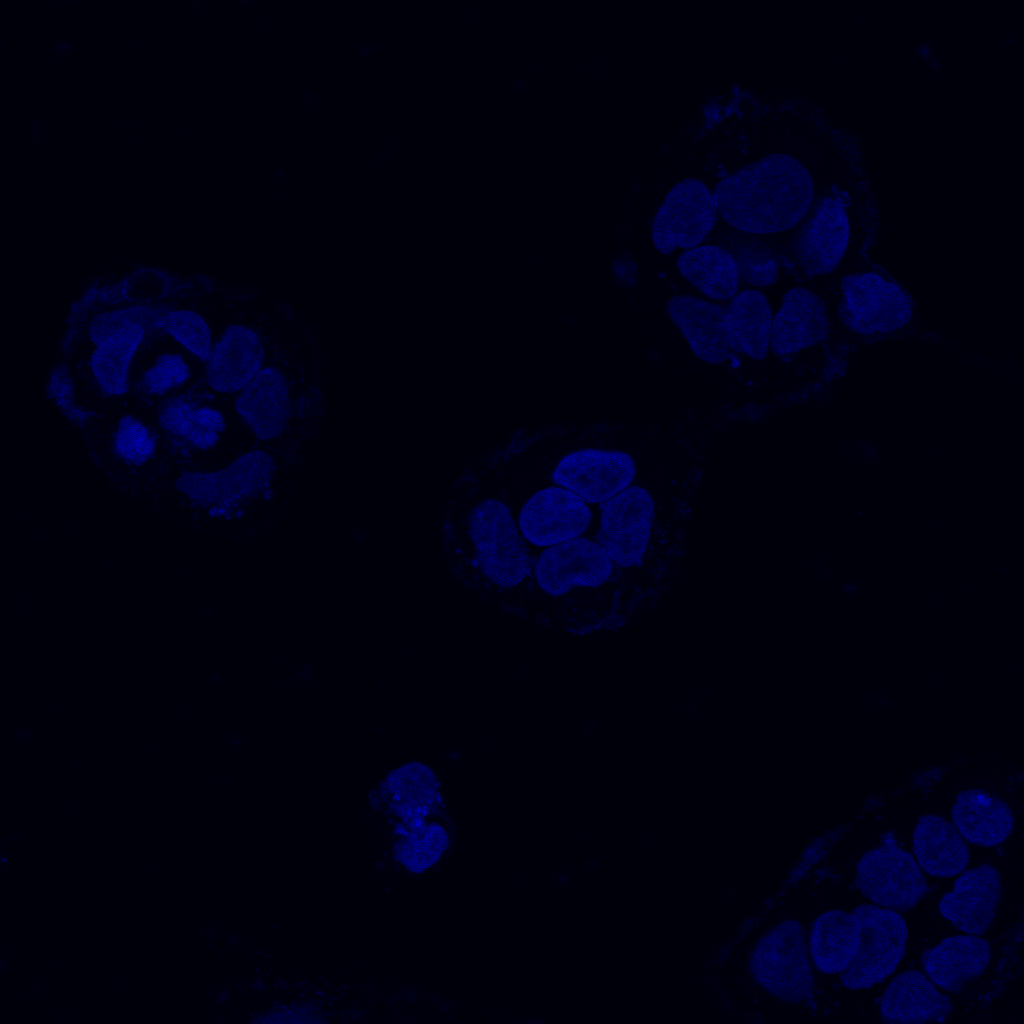

Supplement: Supplementary file 10 — Source data Fig. 8 [file 44321_2024_128_MOESM10_ESM.zip › EMM-2023-19008-V2-figure 8/figure 8A/ú¿3ú⌐ shSAT1#03 duo594-SAT1488-20x3-3.tif (3).frames/shSAT1#3duo594-SAT1488-20x3-2_C001T001.tif]

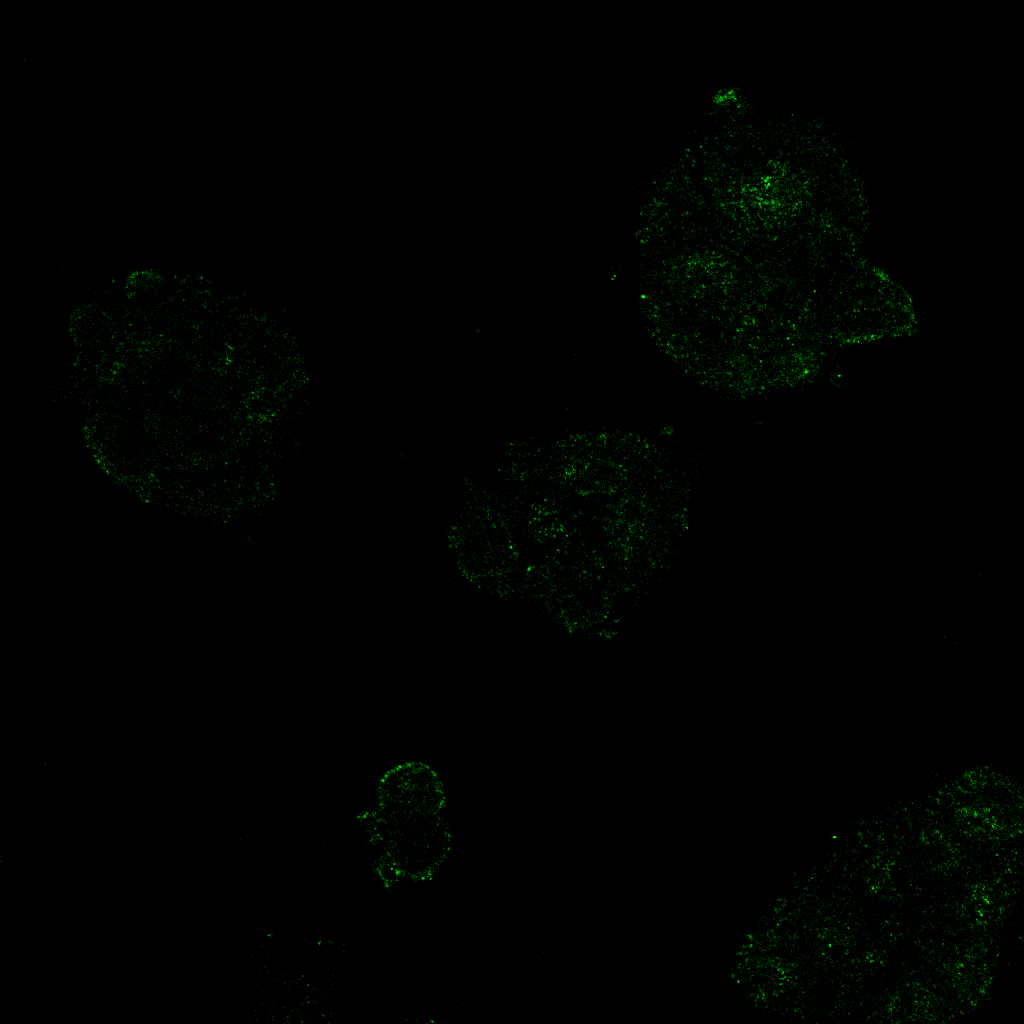

Supplement: Supplementary file 10 — Source data Fig. 8 [file 44321_2024_128_MOESM10_ESM.zip › EMM-2023-19008-V2-figure 8/figure 8A/ú¿3ú⌐ shSAT1#03 duo594-SAT1488-20x3-3.tif (3).frames/shSAT1#3duo594-SAT1488-20x3-2_C002T001.tif]

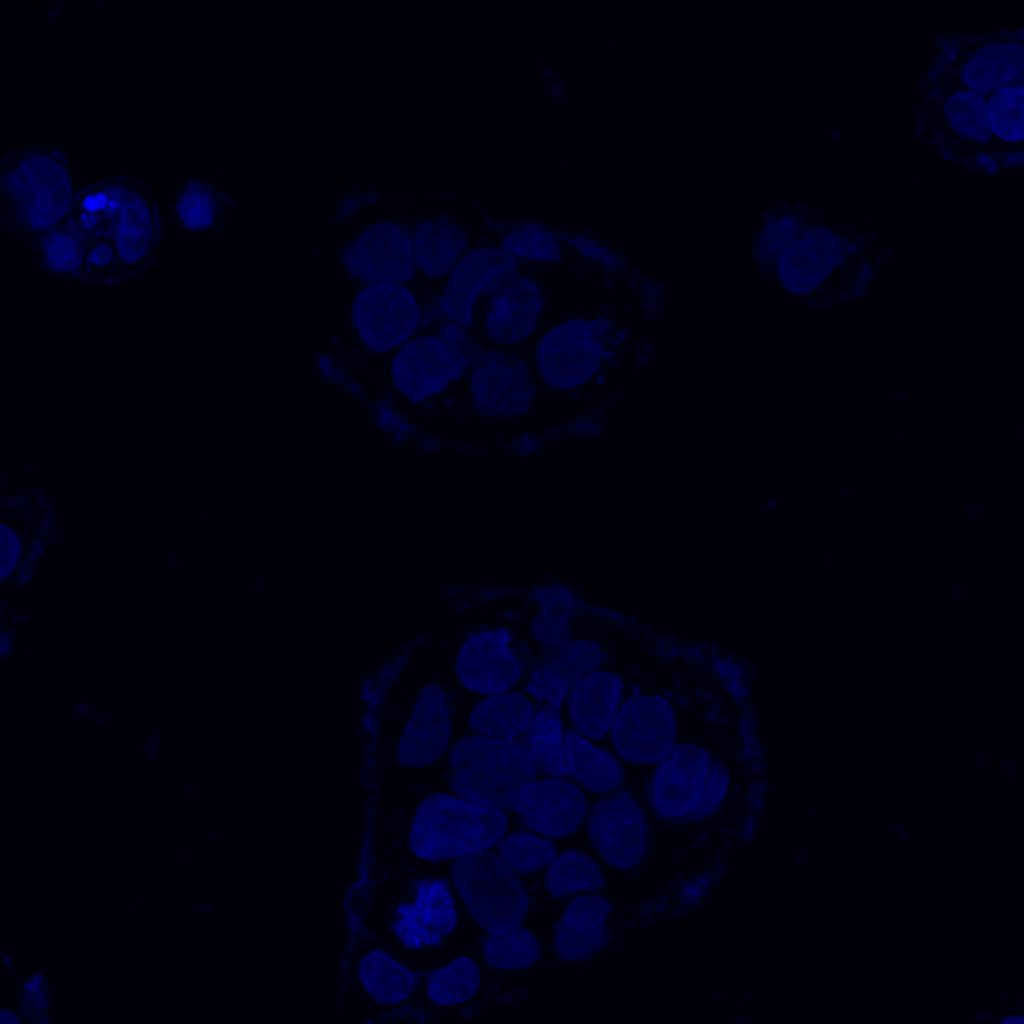

Supplement: Supplementary file 10 — Source data Fig. 8 [file 44321_2024_128_MOESM10_ESM.zip › EMM-2023-19008-V2-figure 8/figure 8A/ú¿4ú⌐ shSAT1#03 +IL-25 duo594-SAT1488-20x3-3.tif (4).frames/shSAT1#3+IL-25 duo594-SAT1488-20x3-1_C001T001.tif]

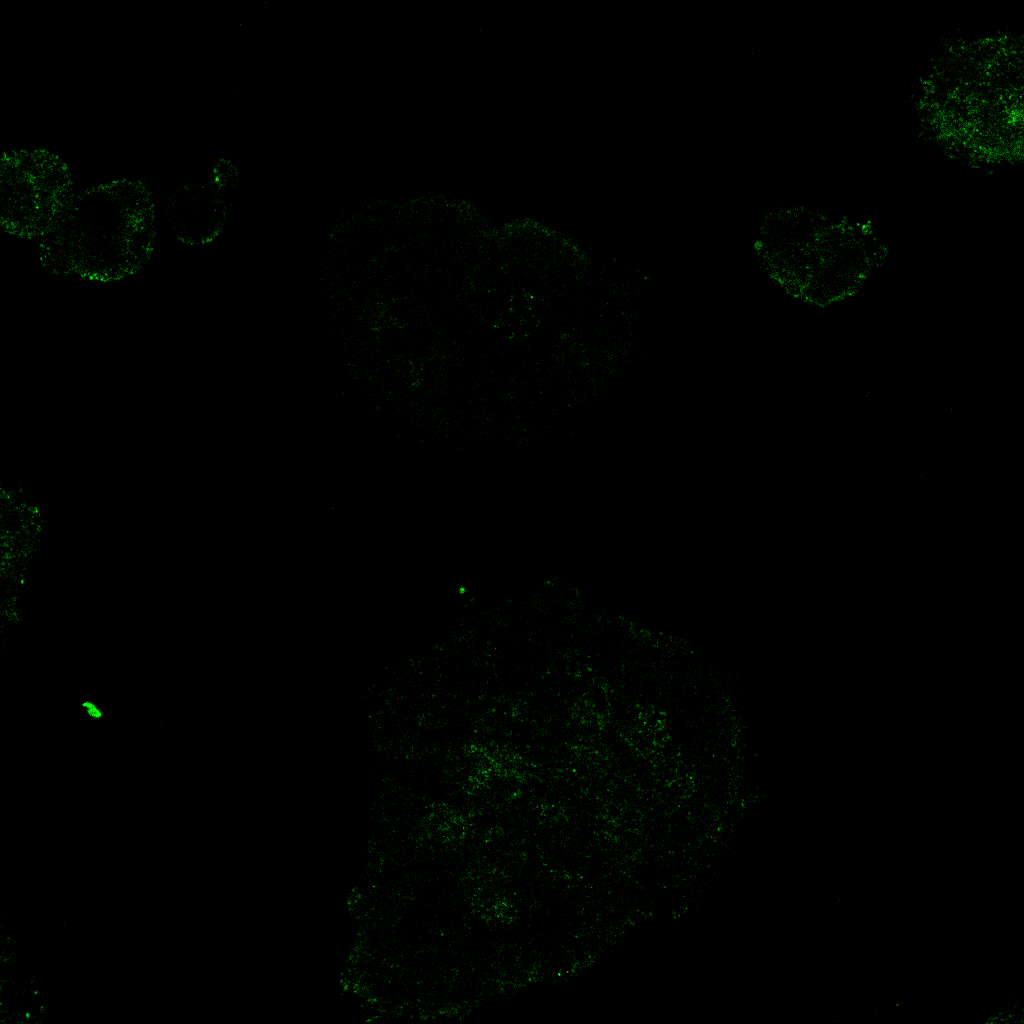

Supplement: Supplementary file 10 — Source data Fig. 8 [file 44321_2024_128_MOESM10_ESM.zip › EMM-2023-19008-V2-figure 8/figure 8A/ú¿4ú⌐ shSAT1#03 +IL-25 duo594-SAT1488-20x3-3.tif (4).frames/shSAT1#3+IL-25 duo594-SAT1488-20x3-1_C002T001.tif]

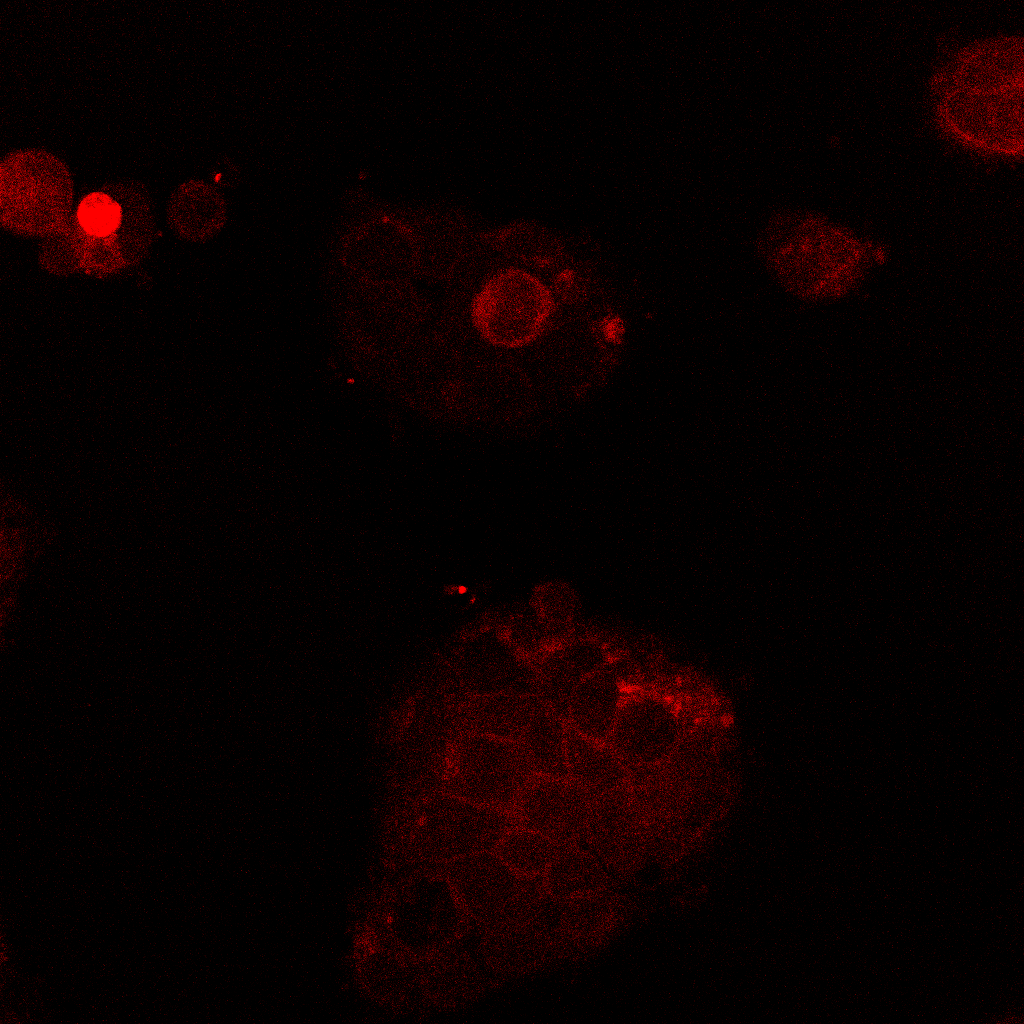

Supplement: Supplementary file 10 — Source data Fig. 8 [file 44321_2024_128_MOESM10_ESM.zip › EMM-2023-19008-V2-figure 8/figure 8A/ú¿4ú⌐ shSAT1#03 +IL-25 duo594-SAT1488-20x3-3.tif (4).frames/shSAT1#3+IL-25 duo594-SAT1488-20x3-1_C003T001.tif]

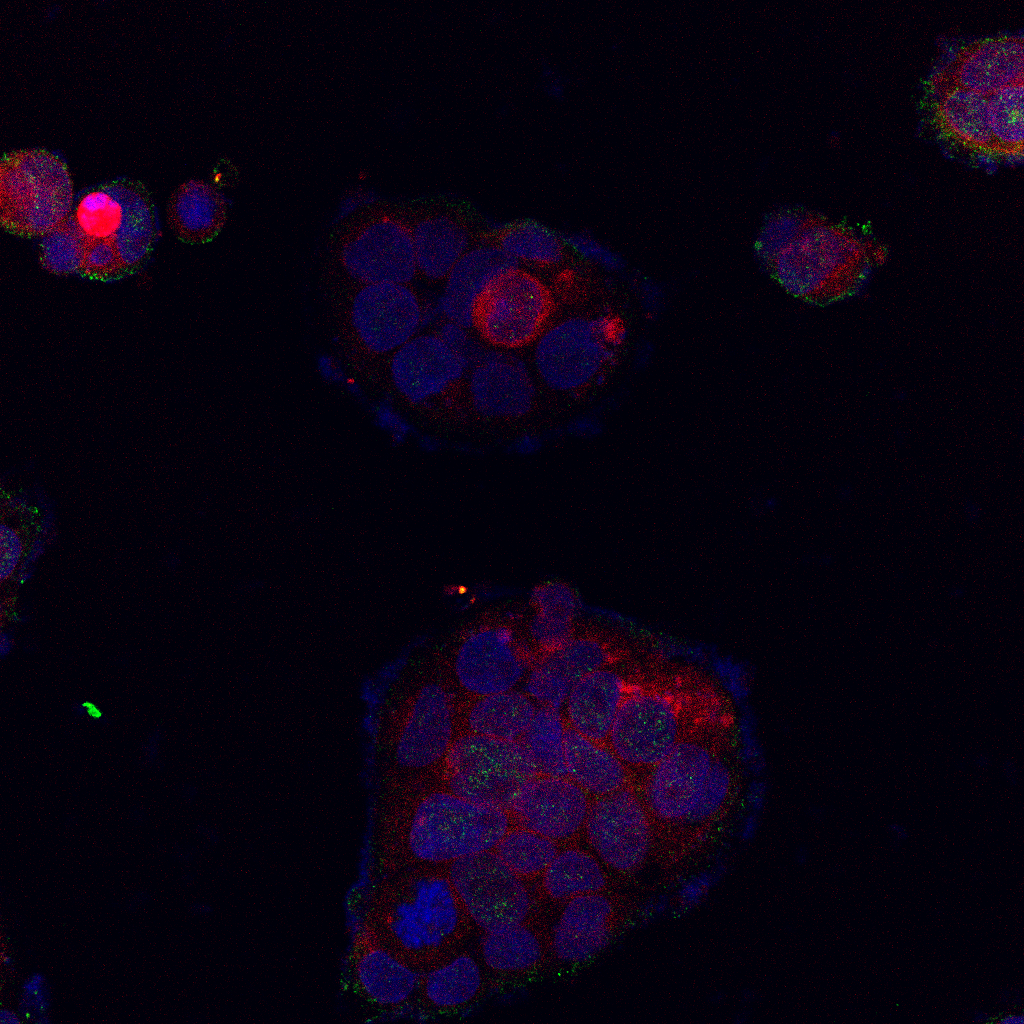

Supplement: Supplementary file 10 — Source data Fig. 8 [file 44321_2024_128_MOESM10_ESM.zip › EMM-2023-19008-V2-figure 8/figure 8A/ú¿4ú⌐ shSAT1#03 +IL-25 duo594-SAT1488-20x3-3.tif (4).frames/shSAT1#3+IL-25 duo594-SAT1488-20x3-1_T001.tif]
