# Supplementary material for: Trained immunity of intestinal tuft cells during infancy enhances host defense against enteroviral infections in mice
Source: EMBO Mol Med. 2024 Sep 11;16(10):2516–38. doi: 10.1038/s44321-024-00128-9 (PMC11479266; doi:10.1038/s44321-024-00128-9)
Supplement: Supplementary file 4 — Source data Fig. 2 [file 44321_2024_128_MOESM4_ESM.zip › EMM-2023-19008-V2-figure 2/figure 2D WB raw data/figure 2D.pptx]

## Slide 1
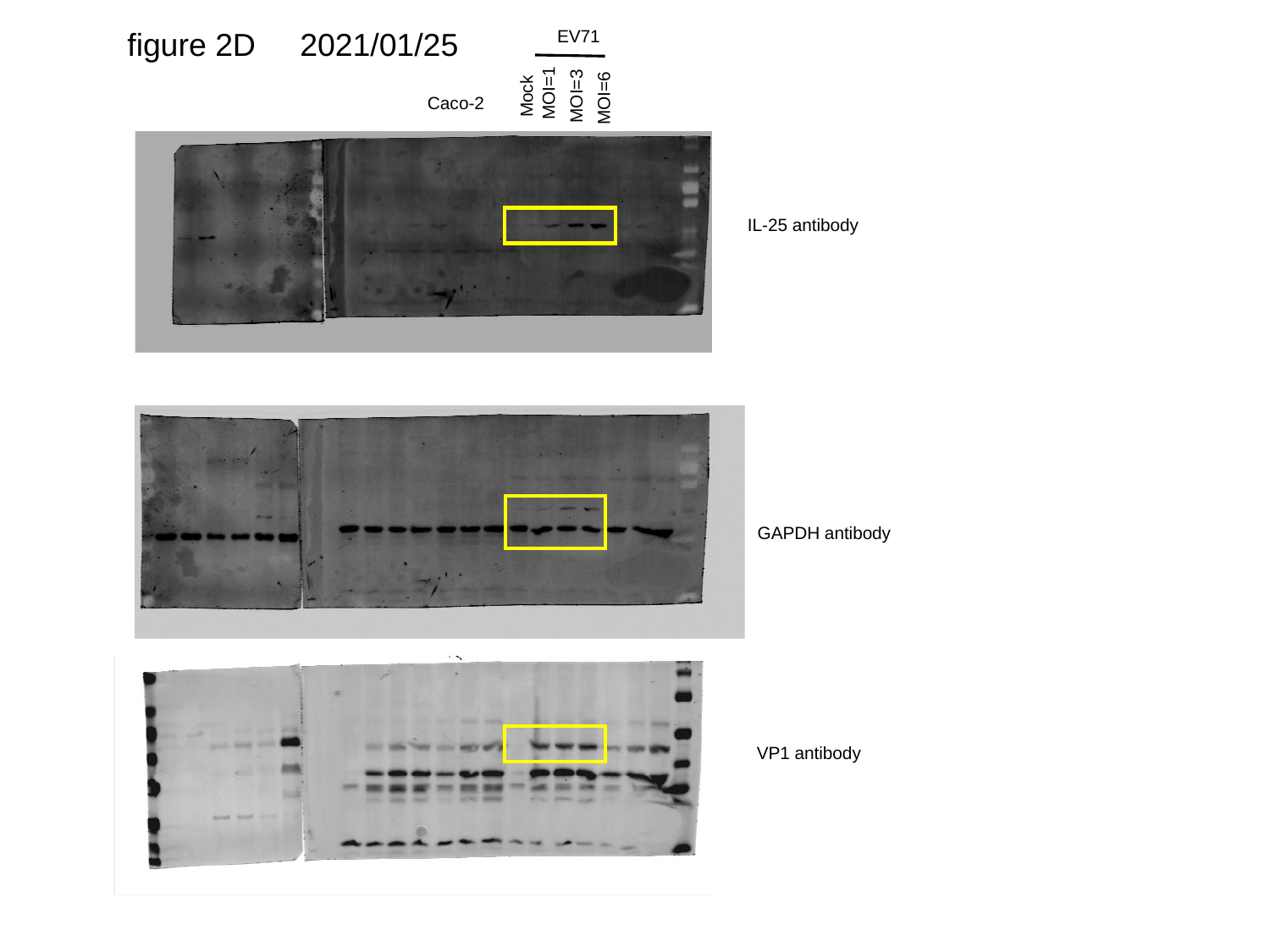

figure 2D 2021/01/25
EV71
Mock
MOI=1
MOI=3
MOI=6
Caco-2
IL-25 antibody
GAPDH antibody
VP1 antibody

## Slide 2
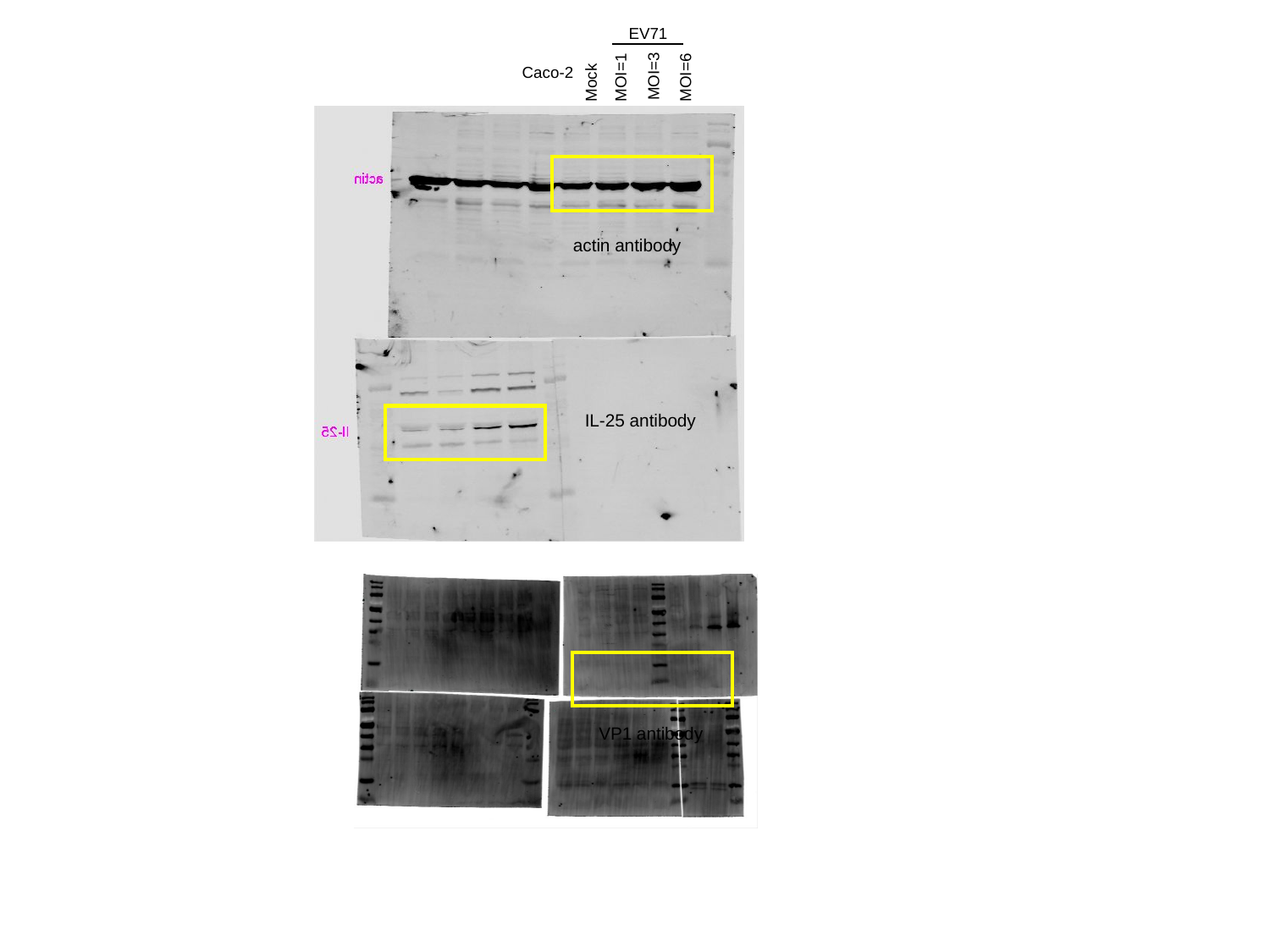

EV71
MOI=6
MOI=1
MOI=3
Caco-2
Mock
actin antibody
IL-25 antibody
VP1 antibody
