# Supplementary material for: Trained immunity of intestinal tuft cells during infancy enhances host defense against enteroviral infections in mice
Source: EMBO Mol Med. 2024 Sep 11;16(10):2516–38. doi: 10.1038/s44321-024-00128-9 (PMC11479266; doi:10.1038/s44321-024-00128-9)
Supplement: Supplementary file 12 — Expanded View Figures [file 44321_2024_128_MOESM12_ESM.pdf]

# Expanded View Figures

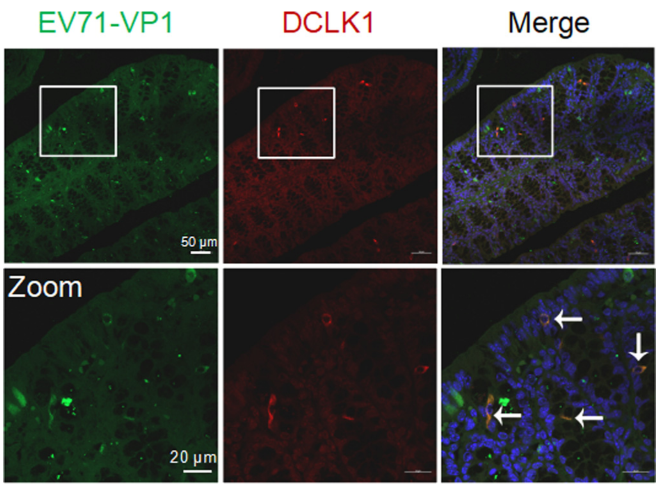


**Figure EV1. EV71 infected tuft cells.**

AG6 male mice were infected with EV71 for 7 dpi, and ileum tissues were collected. Immunofluorescence assays were used to detect EV71-viral protein VP1 expression (Green) and DCLK1 expression (Red). Scale bar: 50 µm. White arrows indicated the co-expression of VP1 and DCLK1. Areas in white squares were shown in threefold magnification on the bottom row. Scale bar: 20 µm. Source data are available online for this figure.

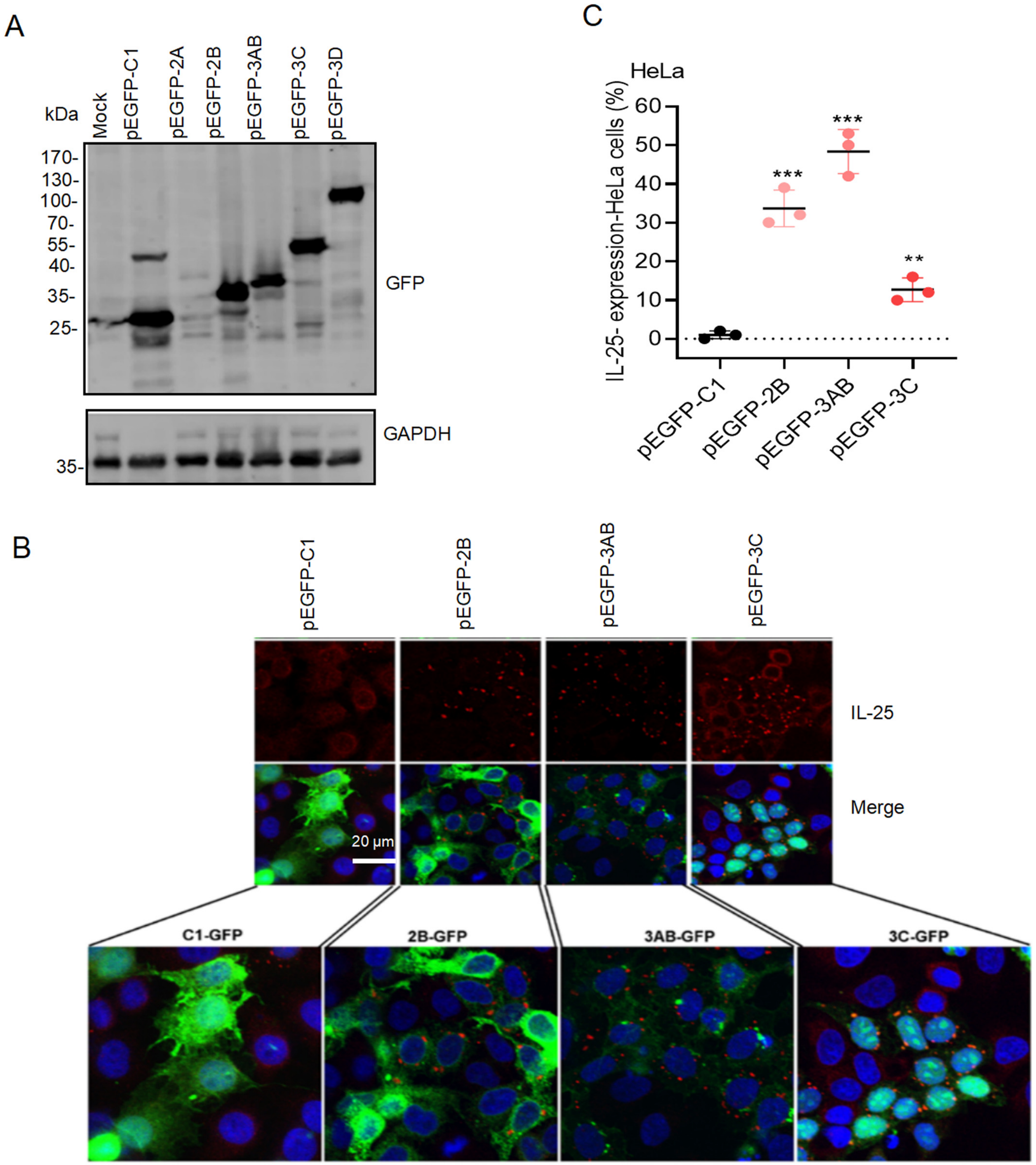


**Figure EV2. EV71 viral protein induced IL-25 production.**

(**A**) Western blotting detected GFP and GAPDH expression in the presence of EV71 2A, 2B, 3AB, 3C, and 3D in 293T cells for 24 h. (**B**) Immunofluorescence staining of IL-25-expressing HeLa cells in the presence of EV71 2B, 3AB, and 3C, respectively. Scale bar: 20 μm. (**C**) quantitative analyses of the number of IL-25-secreted-HeLa cells from (**B**) were performed. Data were presented as mean ± SD ($n = 3$ biological replicates). The major statistical procedures applied were: Shapiro-Wilk test and *F*-test (**B**, **C**), one-way ANOVA and SNK-q test (**B**, **C**). **$P < 0.01$,***$P < 0.001$ vs. the control cells (p-EGFP-C1-group). Source data are available online for this figure.

 © The Author(s)

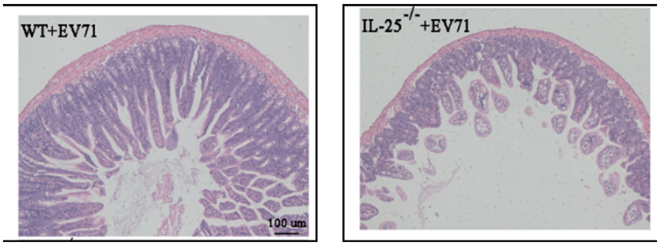


**Figure EV3. Ileum tissues from EV71-infected IL-25$^{-/-}$ mice and WT mice at 7 dpi were stained with H&E.**

Scale bar: 100 µm. Source data are available online for this figure.

© The Author(s)
